# Gene expression profiling before and after internode culture for adventitious shoot formation in ipecac

**DOI:** 10.1186/s12870-022-03756-w

**Published:** 2022-07-22

**Authors:** Karin Okazaki, Imari Koike, Sayuri Kera, Katushi Yamaguchi, Shuji Shigenobu, Koichiro Shimomura, Mikihisa Umehara

**Affiliations:** 1 Graduate School of Life Sciences, Toyo University, 1-1-1 Izumino, Itakura-machi, Ora-gun, Gunma 374-0193 Japan; 2 Trans-Scale Biology Center, National Institute for Basic Biology, 38 Nishigonaka, Myodaiji, Okazaki, Aichi 444-8585 Japan; 3 Department of Applied Biosciences, Toyo University, 1-1-1 Izumino, Itakura-machi, Ora-gun, Gunma 374-0193 Japan

**Keywords:** Adventitious shoot, *Carapichea ipecacuanha*, *De novo* transcriptome assembly, Internodal segment, RNA-seq, Transcriptional change

## Abstract

**Background:**

In ipecac (*Carapichea ipecacuanha* (Brot.) L. Andersson), adventitious shoots can be induced simply by placing internodal segments on phytohormone-free culture medium. The shoots form locally on the epidermis of the apical region of the segments, but not the basal region. Levels of endogenous auxin and cytokinin transiently increase in the segments after 1 week of culture.

**Results:**

Here, we conducted RNA-seq analysis to compare gene expression patterns in apical and basal regions of segments before culture and after 1 week of culture for adventitious shoot formation. The results revealed 8987 differentially expressed genes in a *de novo* assembly of 76,684 genes. Among them, 276 genes were upregulated in the apical region after 1 week of culture relative to before culture and the basal region after 1 week of culture. These genes include 18 phytohormone-response genes and shoot-formation-related genes. Validation of the gene expression by quantitative real-time PCR assay confirmed that the expression patterns were similar to those of the RNA-seq data.

**Conclusions:**

The transcriptome data show that expression of cytokinin biosynthesis genes is induced along with the acquisition of cellular pluripotency and the initiation of cell division by wounding in the apical region of internodal segments, that trigger adventitious shoot formation without callusing.

**Supplementary Information:**

The online version contains supplementary material available at 10.1186/s12870-022-03756-w.

## Background

In 1902, Gottlieb Haberlandt proposed that plant cells can divide and differentiate to form the whole plant body [1]. The concept is called ‘totipotency’. Since whole plants were regenerated from carrot tissue segments [2], plant tissue culture has been used as a fundamental technique to regenerate plantlets from plant cells, tissues, or organs isolated from mother plants, on culture media [3]. Nowadays, it is an essential technique for production of virus-free plants, rapid multiplication of rare plant genotypes, genetic transformation, and production of commercially valuable plant-derived secondary metabolites, as well as for basic and applied research in plant science [4]. The balance of exogenously applied auxin and cytokinin (CK) concentrations affects organogenesis: a high ratio of auxin to CK induces adventitious roots, a low ratio induces adventitious shoots, and a high concentration of both hormones induces callus [5]. In general, *de novo* shoot and root organogenesis require the addition of phytohormones, such as auxin and CK, into the culture media in plant tissue culture [6].

Understanding the molecular mechanism of *de novo* plant organogenesis is important because it addresses many fundamental questions in developmental biology. So far, the molecular mechanism of plant regeneration has been studied widely in a genetically tractable model plant, *Arabidopsis thaliana* L. [7]. In the Arabidopsis culture system, phytohormone treatment is critical to inducing plant regeneration via two steps: callus induction of root or hypocotyl segments on auxin-rich medium, and subsequent shoot regeneration from the callus on CK-rich medium. In the first step, auxin promotes callus formation via AUXIN RESPONSE FACTOR (ARF)-mediated activation of LATERAL ORGAN BOUNDARIES DOMAIN proteins (LBDs) [8]. Auxin also promotes the acquisition of cellular pluripotency via activation of LBD16, PLETHORA proteins (PLTs), and CUP-SHAPED COTYLEDON proteins (CUCs) [9, 10]. In the second step, CKs promote shoot meristem formation via ARABIDOPSIS RESPONSE REGULATOR (ARR)-mediated activation of WUSCHEL (WUS) expression [11–14]. Other transcription factors, SHOOT MERISTEMLESS (STM) and RAP2.6 L, also promote shoot meristem formation independent of CK signaling [15–17]. In addition, wounding can induce callus formation and shoot regeneration. Wounding promotes expression of CK biosynthesis genes such as *ISOPENTENYL TRANSFERASE 3* (*IPT3*) and *LONELY GUYs* (*LOGs*) [18], and AP2/ERF transcription factors such as *WOUND-INDUCED DEDIFFERENTATION 1* (*WIND1*) and its homologs [19] in callus formation at the wounding site. Expression of these genes activates CK signaling mediated by type-B ARRs, inducing cell division via upregulation of CYCLIN D3;1 (CYCD3;1). Wounding upregulates other AP2/ERF transcription factors, including PLTs [18]. In shoot regeneration, ENHANCER OF SHOOT REGENERATION​ 1 (ESR1) and ESR2 work downstream of WIND1 and CK signaling [20].

On the other hand, adventitious shoots can be induced by placing explants on culture media without phytohormones in some plant species, such as *Dianthus caryophyllus* L. [21], *Aegle marmelos* L. Corrêa [22], *Bacopa monnieri* (L.) Wettst [23]., *Celastrus paniculatus* Willd [24]., *Kalanchoë blossfeldiana* Poelln [25]., and *Carapichea ipecacuanha* (Brot.) L. Andersson (ipecac) [26]. Ipecac is a medicinal plant that contains alkaloids such as emetine and cephaeline, which are used in expectorants, emetics, and amebicides, in its roots [27, 28]. Adventitious shoot formation of ipecac can be observed 4 weeks after internodal segments are placed on phytohormone-free culture media [26]. This rare characteristic allows us to analyze the dynamics and effects of endogenous phytohormones during adventitious shoot formation, and to easily evaluate the direct effects of exogenously applied chemicals. Adventitious shoots are locally formed on the epidermis of the apical region of internodal segments, but not in basal region [29]. Endogenous levels of indole-3-acetic acid (IAA) are low in the apical region of internodal segments and high in the basal region [29]. These data indicate a negative relation between the position of adventitious shoots formed and IAA distribution in internodal segments. By using inhibitors of polar auxin transport, we found that endogenous IAA is transported from the apical to basal region in the internodal segments, maintaining the IAA levels in the apical region at a low concentration appropriate for adventitious shoot formation [30]. However, the molecular basis for how ipecac cells initiate shoot meristem formation is still undiscovered.

Since the adventitious shoots of ipecac are formed only in the apical region of internode segments, we expect that gene expression patterns differ between the apical and basal regions, so differential expression analysis should reveal the genes that control adventitious shoot formation in ipecac. We consider that excision of the internode section is the first trigger for adventitious shoot formation in ipecac, and cause dynamic gene reprogramming in the section. In addition, endogenous IAA and CKs transiently increase in the internodal segments after 1 week of culture [29]. Thus, we hypothesized that the expression of genes related to adventitious shoot formation is induced in the apical region of internodal segments after 1 week of culture. Here, we conducted RNA-seq analysis to compare gene expression patterns in apical and basal regions of internodal segments before and after 1 week of culture.

## Results

### Sequence generation by RNA-seq and *de novo* assembly of transcripts

**Fig. 1:**
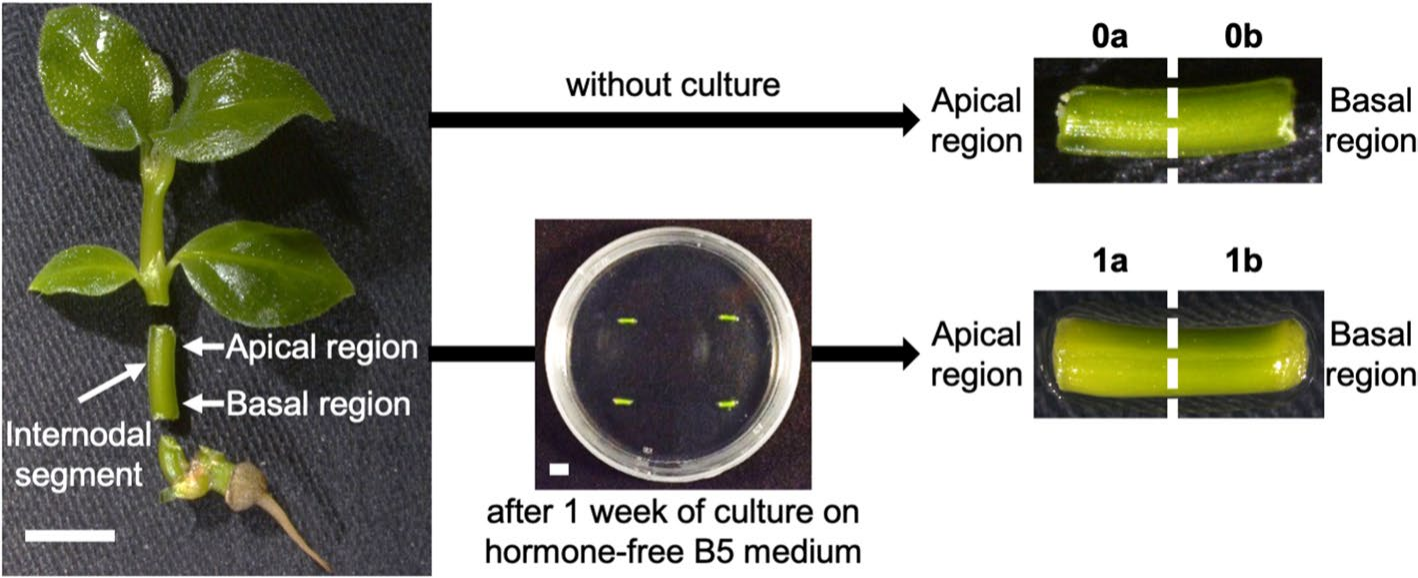
Sample preparation for RNA-seq analysis. Internodal segments (5 mm in length) were cut and divided into apical and basal regions before or after 1 week of culture on hormone-free B5 medium. 0a, apical region without culture; 0b, basal region without culture; 1a, apical region after 1 week of culture; 1b, basal region after 1 week of culture. *n* = 12 in 0a and 0b and 8 in 1a and 1b. Bar, 5 mm

**Fig. 2:**
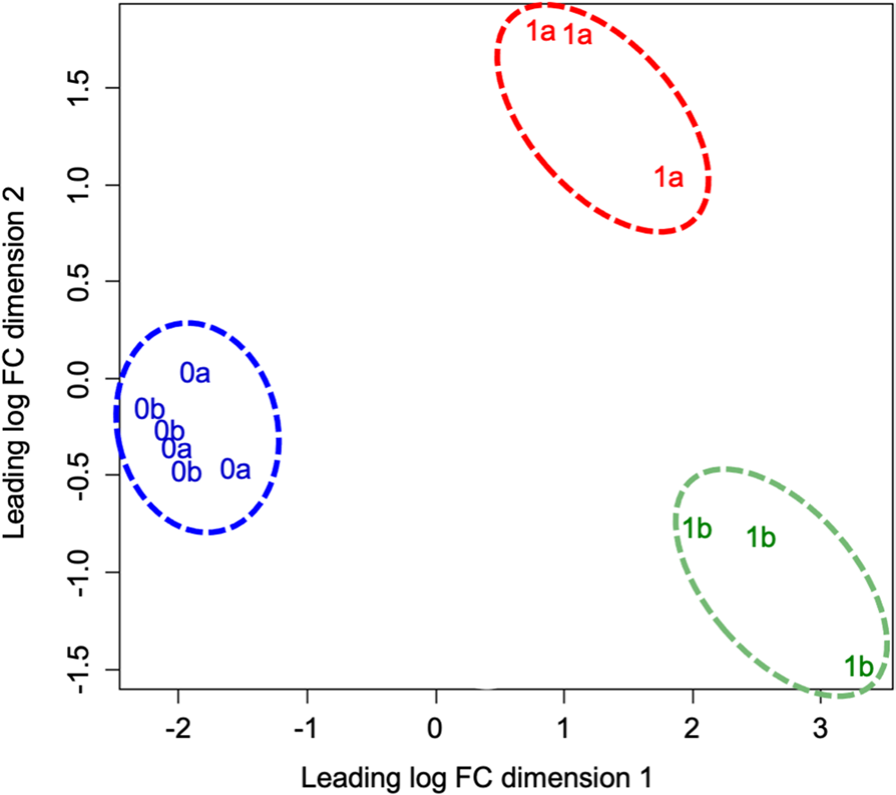
Multidimensional scaling (MDS) plot of gene expression in 0a, 0b, 1a, and 1b samples. 0a, apical region without culture, 0b, basal region without culture; 1a, apical region after 1 week of culture; 1b, basal region after 1 week of culture

To explore genes controlling adventitious shoot formation in ipecac, we analyzed the RNA-seq transcriptome of internodal segments. RNA was isolated from four sample types of internodal segments: apical region before culture (0a), basal region before culture (0b), apical region after 1 week of culture (1a), and basal region after 1 week of culture (1b) (Fig. 1). RNA of leaves and roots was used as reference. A total of 16 samples (3 each of 0a, 0b, 1a, and 1b; 2 each of leaves and roots) were sequenced by RNA-seq to generate comprehensive transcriptome sequences. Sequencing of the 16 libraries generated 113,423,893 paired-end reads with good quality (Table 1). Multi-dimensional scaling classified gene expression patterns in the segments into three clusters: 0a + 0b, 1a, and 1b (Fig. 2).

**Table 1:** RNA-Seq paired-read counts for all samples used for *de novo* assembly

sample name	read counts	mapped paired-read (%)	mapped paired-read (ORF^a^) (%)
leaf-1	7,638,583	98.9	65.9
leaf-2	6,666,534	98.8	65.2
root-1	8,239,930	99.0	67.2
root-2	6,675,989	98.9	67.8
0a-1	8,245,866	99.0	67.3
0a-2	6,816,171	99.0	65.8
0a-3	7,092,896	99.1	66.9
0b-1	7,597,324	98.9	66.5
0b-2	7,038,414	99.0	67.7
0b-3	6,752,651	99.0	67.2
1a-1	7,160,555	98.9	66.5
1a-2	6,522,547	99.0	67.0
1a-3	5,658,138	99.0	67.4
1b-1	7,993,239	99.0	68.4
1b-2	6,991,590	99.0	68.3
1b-3	6,333,466	99.0	68.8
total	113,423,893		

^a^*ORF* open reading frame

All RNA-seq reads were *de novo* assembled with Trinity software, resulting in 189,603 contigs that grouped into 76,684 isoform clusters (i.e., unigenes). From the transcriptome sequences, we extracted and selected 62,491 non-redundant (nr) open reading frames (ORFs) with N50 of 1122 bp. The completeness of the predicted ORFs were evaluated using BUSCOs. The ORF sets contained 92.1 and 86.3% of eukaryote and embryophyte BUSCOs (ver. odb9), respectively. All translated sequences of the ORF set were compared with the NCBI nr protein database using BLASTP. Among the ORFs, 35,298 (56.4%) hit protein sequences in the NCBI nr database. The most frequent BLASTP top-hit species was *Coffea canephora* (20,169, 32.3%), which belongs to the Rubiaceae along with ipecac.

### Profiling of differential gene expression in internodal segments

**Fig. 3:**
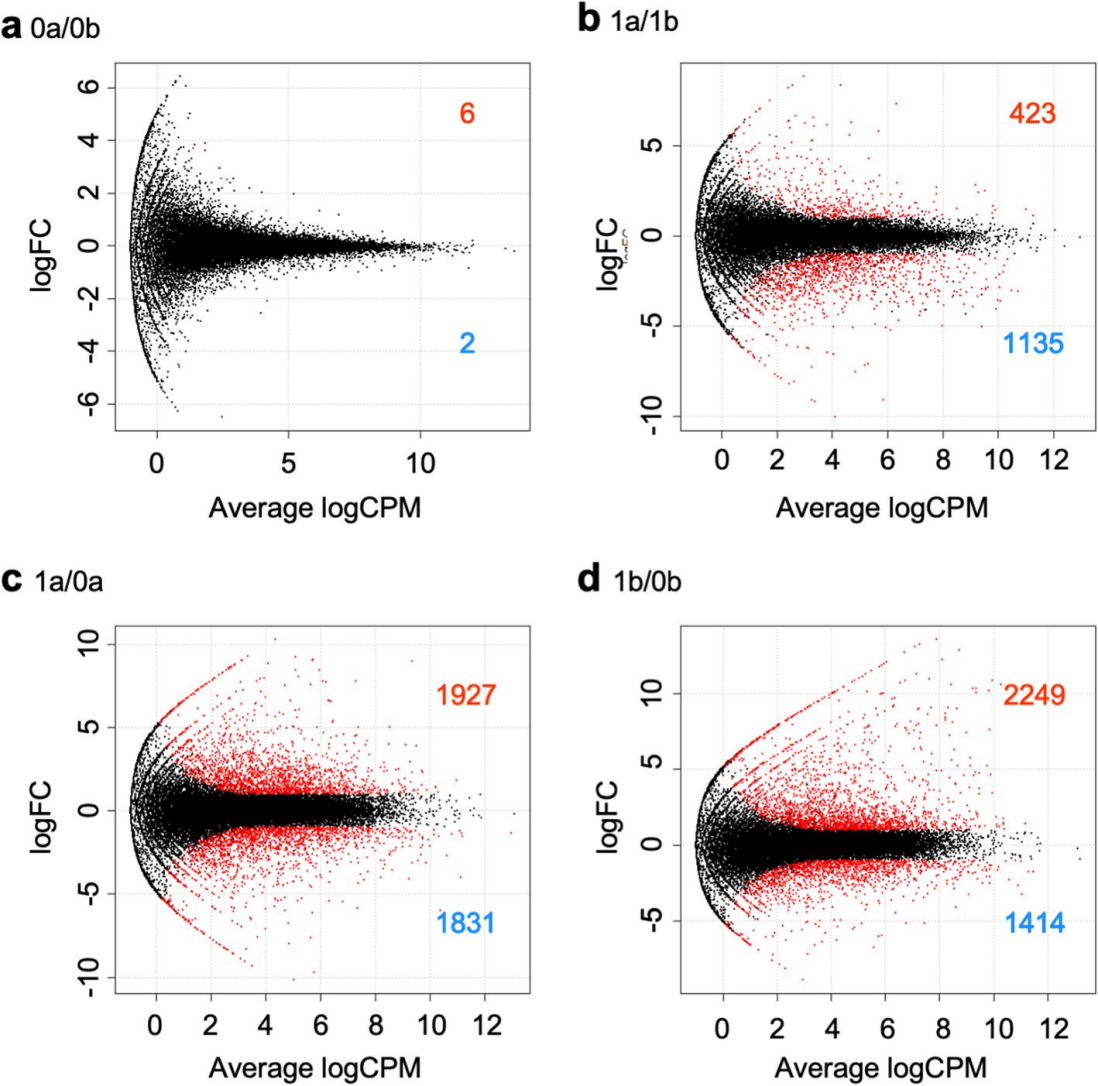
MA plots of differentially expressed genes (DEGs) between (**a**) 0a and 0b, (**b**) 1a and 1b, (**c**) 1a and 0a, and (**d**) 1a and 0a. 0a, apical region without culture; 0b, basal region without culture; 1a, apical region after 1 week of culture; 1b, basal region after 1 week of culture. Colored numbers indicate numbers of (red) upregulated and (blue) downregulated genes (log FC > |1|, FDR < 0.01)

**Fig. 4:**
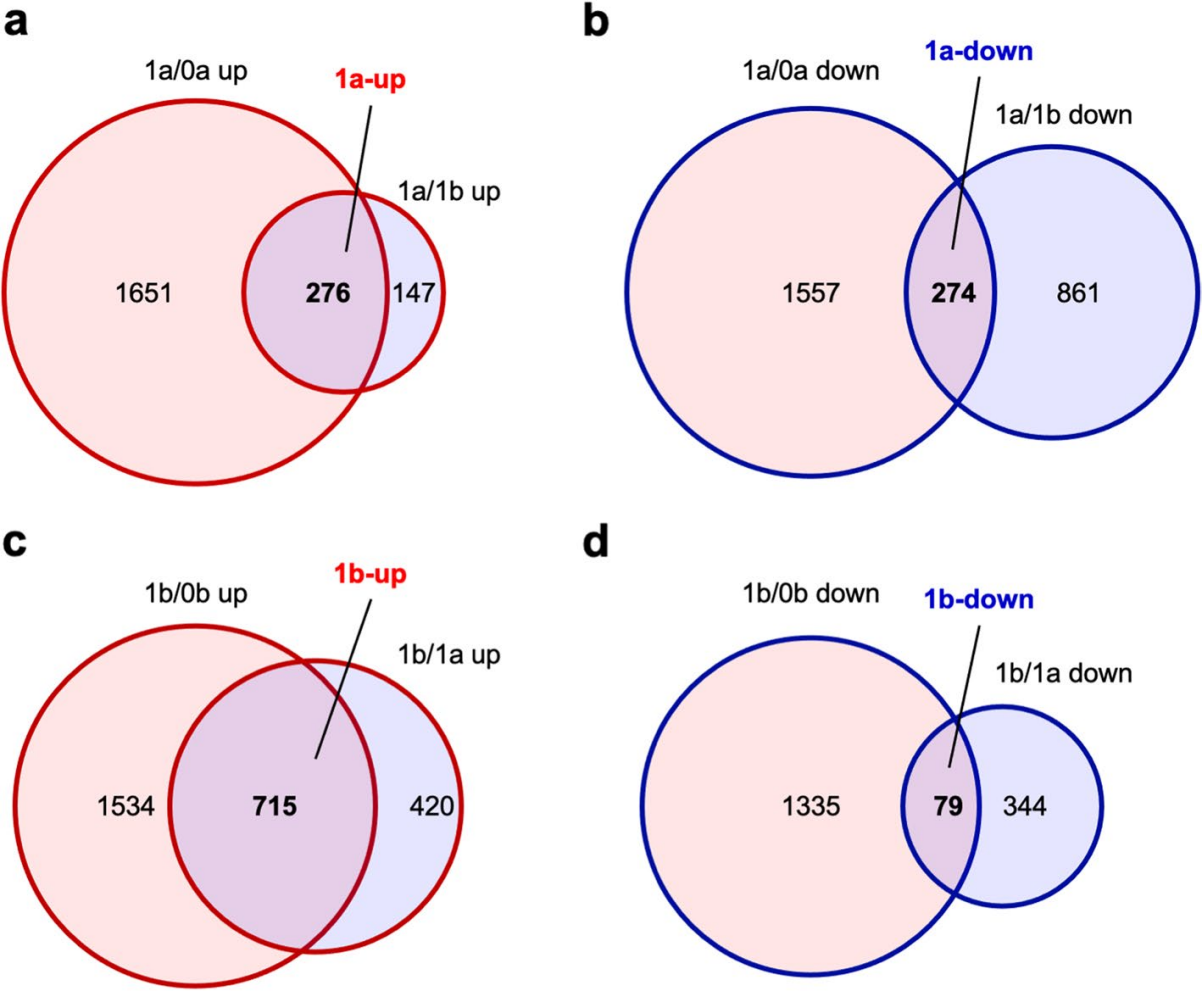
Venn diagrams of numbers of DEGs. **a** Upregulated in 1a relative to 0a and 1b (1a-up). **b** Downregulated in 1a relative to 0a and 1b (1a-down). **c** Upregulated in 1b relative to 0b and 1a (1b-up). **d** Downregulated in 1b relative to 0b and 1a (1b-down). 0b, basal region without culture; 1a, apical region after 1 week of culture; 1b, basal region after 1 week of culture (log FC > |1|, FDR < 0.01)

To compare spatiotemporal gene expression patterns in internodal segments, we analyzed abundances of differentially expressed genes (DEGs) mapped on the assembled transcriptome as reference sequences. We compared transcripts of apical and basal regions of internodal segments and transcripts before and after culture in the same internodal region. Among 8987 differentially expressed genes, we found 6 genes upregulated in 0a relative to 0b (0.0096%), 423 in 1a relative to 1b (0.68%), 1927 in 1a relative to 0a (3.1%), and 2249 in 1b relative to 0b (3.6%) (Fig. 3a, b). We found 2 genes downregulated in 0a relative to 0b (0.0032%), 1135 in 1a relative to 1b (1.8%), 1831 in 1a relative to 0a (2.9%), and 1414 in 1b relative to 0b (2.3%) (Fig. 3c, d). To find region-specific genes, we narrowed down the number of genes whose expression changed in 1a and 1b. In 1a, 276 genes were upregulated (defined as “1a-up”) and 274 genes were downregulated (“1a-down”) relative to both 0a and 1b (Fig. 4a, b; Tables S2, S3). In 1b, 715 genes were upregulated (“1b-up”) and 79 genes were downregulated (“1b-down”) relative to both 0b and 1a (Fig. 4c, d; Tables S4, S5).

### Enrichment analysis of gene ontology (GO)

**Fig. 5:**
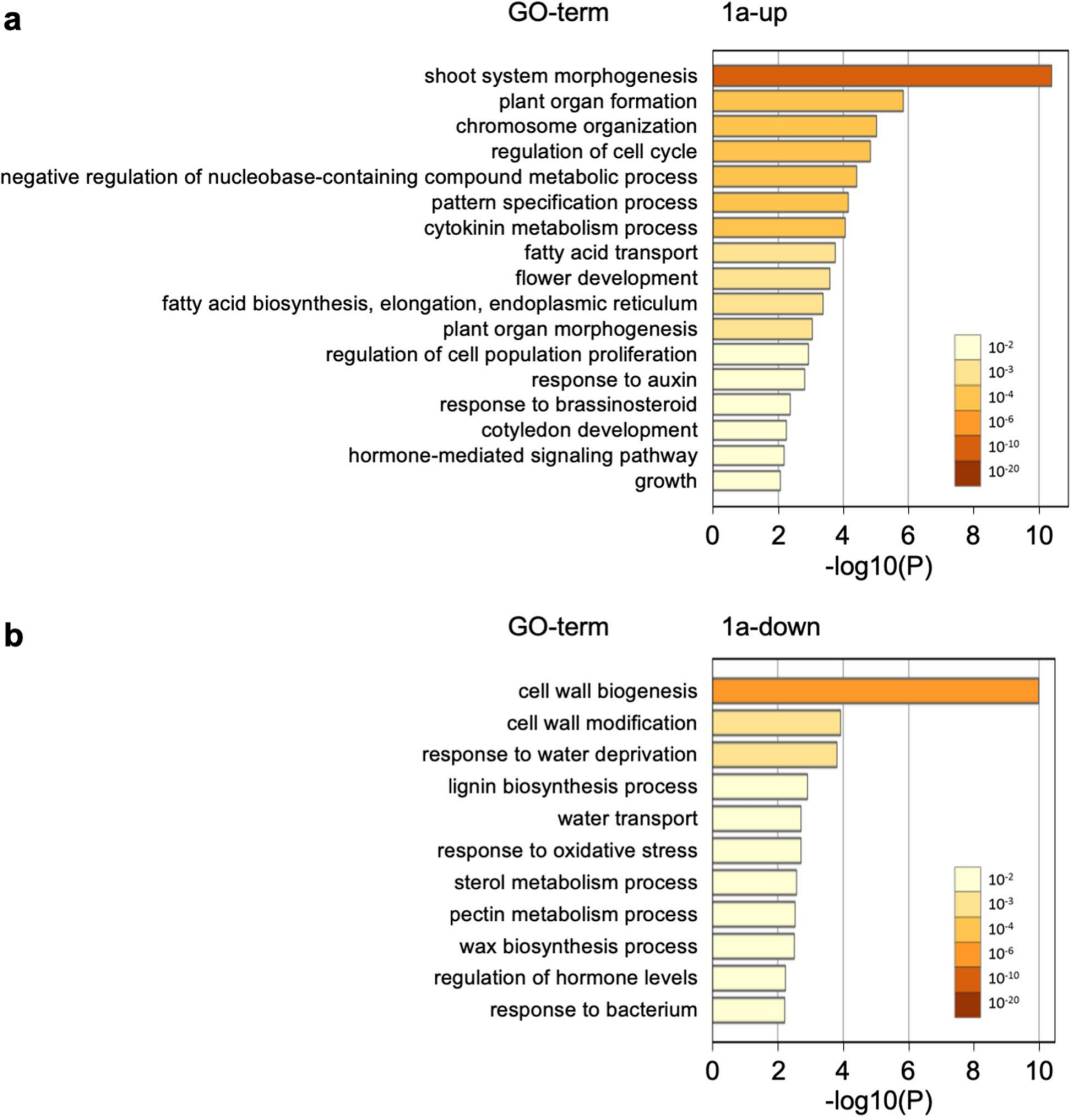
GO enrichment analysis of DEGs in apical region: GO terms enriched in **a** 1a-up and **b** 1a-down. 1a, apical region after 1 week of culture

**Fig. 6:**
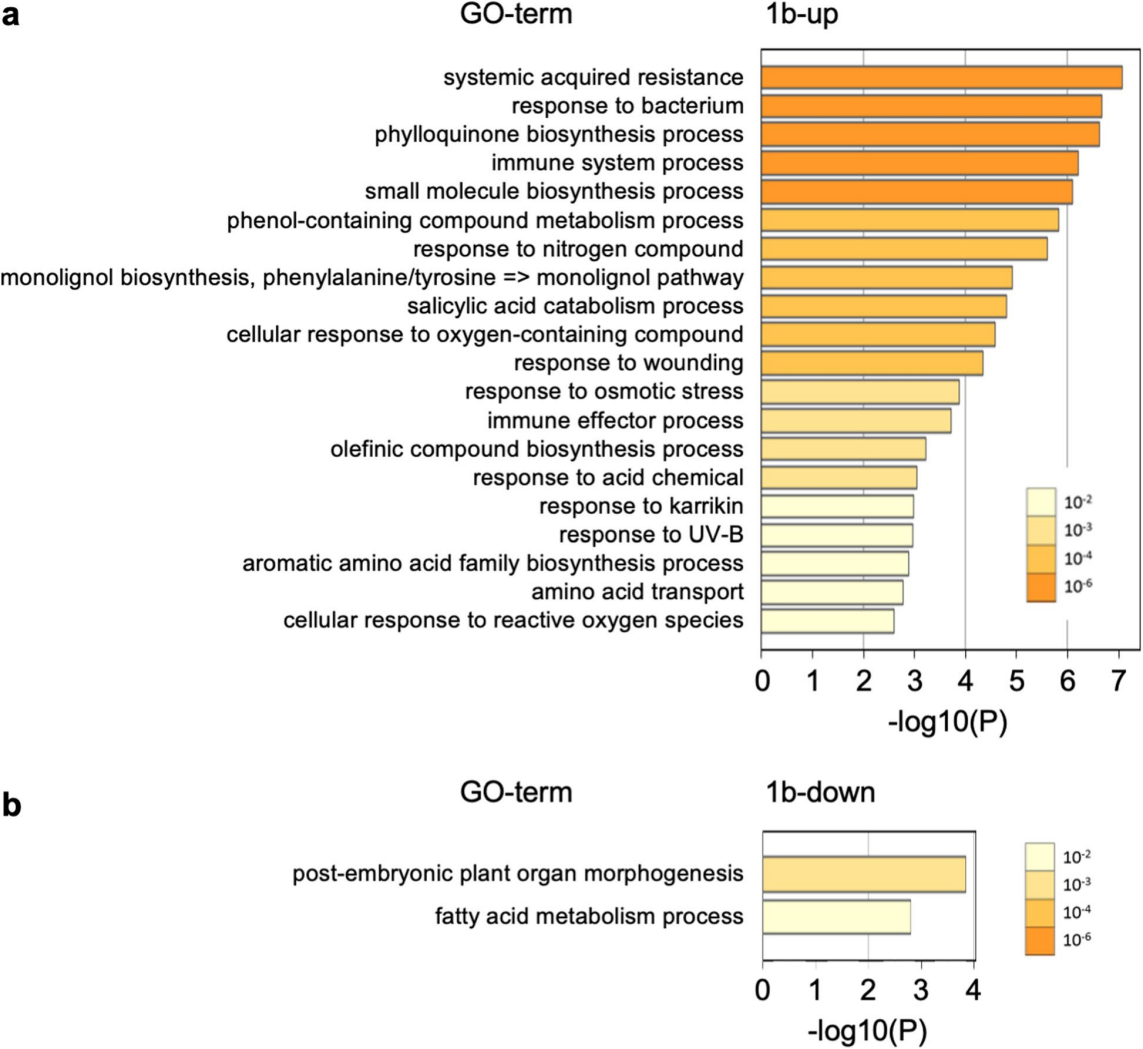
GO enrichment analysis of DEGs in basal region: GO terms enriched in **a** 1b-up and **b** 1b-down. 1b, basal region after 1 week of culture

Next, we investigated GO terms enriched in each of the 1a-up, 1a-down, 1b-up, and 1b-down groups (Figs. 5, 6). The most enriched GO term in 1a-up was shoot system morphogenesis (GO: 0010016; Fig. 5a). In addition, plant organ formation (GO: 1905393), chromosome organization (GO: 0051276), regulation of cell cycle (GO: 0051726), pattern specification process (GO: 0007389), plant organ morphogenesis (GO: 1905392), and regulation of cell population proliferation (GO: 0042127) were detected as terms associated with organogenesis and cell division. Group 1a-up also included terms related to phytohormone signaling: cytokinin metabolism process (GO: 0009690), response to auxin (GO: 0009733), response to brassinosteroid (GO: 0009741), and hormone-mediated signaling pathway (GO: 0009755). In 1a-down, GO terms related mainly to primary cell wall decomposition and secondary cell wall biosynthesis were enriched: cell wall biogenesis (GO: 0042546), cell wall modification (GO: 0042545), lignin biosynthetic process (GO: 0009809), sterol metabolic process (GO: 0016125), and pectin metabolic process (GO: 0045488) (Fig. 5b). In 1b-up, GO terms related mainly to plant immunity and wounding response were detected: systemic acquired resistance (GO: 0009627), response to bacterium (GO: 0009617), immune system process (GO: 0002376), response to wounding (GO: 0009611), and immune effector process (GO: 0002252) (Fig. 6a). In 1b-down, only two GO terms were detected: post-embryonic plant organ morphogenesis (GO: 0090697) and fatty acid metabolism process (GO: 0006631) (Fig. 6b). These GO terms were all categorized into biological process.

### Quantification of DEGs selected in 1a-up by qRT-PCR

**Fig. 7:**
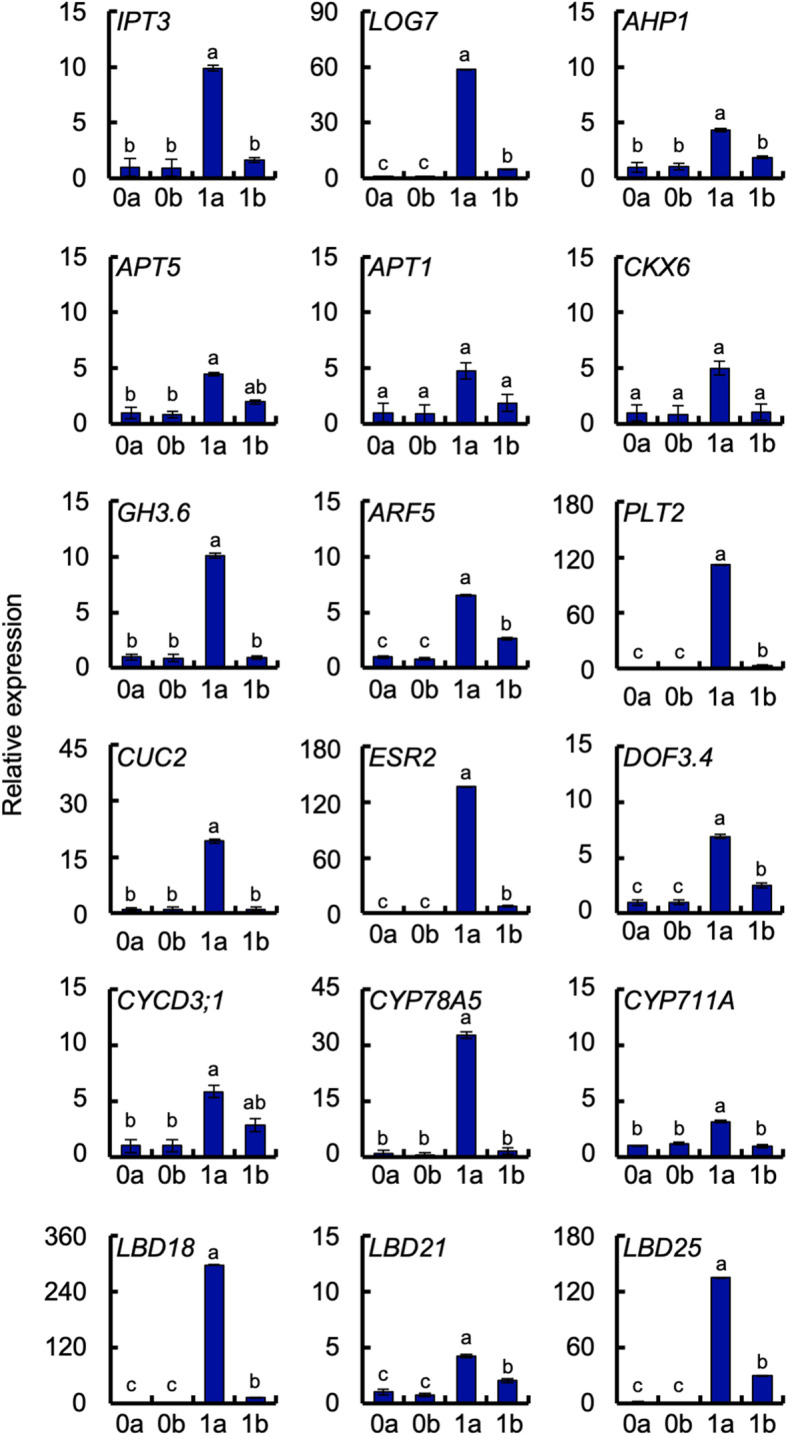
Relative expression of phytohormone-response genes and shoot-formation-related genes. Data are means ± SE (*n* = 4). Eight segments were used in each experiment. *EF-1* was used as an internal standard. 0a, apical region without culture; 0b, basal region without culture; 1a, apical region after 1 week of culture; 1b, basal region after 1 week of culture. Different letters indicate significant differences among segments (Tukey’s HSD, *P* < 0.05)

To validate the DEG data obtained by RNA-seq, we analyzed 18 genes selected from 1a-up: for auxin metabolism (*GRETCHEN HAGEN 3.6* (GH3.6)), auxin signaling (*ARF5*), CK biosynthesis (*IPT3*, *LOG7*), CK metabolism (*ADENINE PHOSPHORIBOSYLTRANSFERASE 1* (*APT1*), *APT5*, *CYTOKININE OXIDASE 6* (*CKX6*)), CK signaling (*Arabidopsis Thaliana HISTIDINE-CONTAINING PHOSPHOTRANSFER PROTEIN 1* (*AHP1*)), strigolactone (SL) biosynthesis (*CYP711A*), plastochron control (*CYP78A5*), cyclin (*CYCD3;1*), and organogenesis (*PLT2*, *CUC2*, *ESR2*, *DOF3.4*, *LBD18*, *LBD21*, *LBD25*) (Table 2). The expression of all of these genes was confirmed by qRT-PCR in 0a, 0b, 1a, and 1b of newly collected internodal segments that were different from RNA-seq samples. Expression of all genes was highest in 1a (Fig. 7). However, there was no significant difference among 0a, 0b, 1a, and 1b in *APT1* (*P* = 0.254) or *CKX6* (*P* = 0.268).

**Table 2:** Cytokinin, auxin, and organogenesis-related genes upregulated in apical region of internodal segments

	1a/0a		1a/1b			
	logFC^a^	FDR^b^	logFC^a^	FDR^b^	description	gene name
TRINITY_DN12309_c0_g1_i2.p1	3.5	9.3E-09	2.2	1.6E-03	adenylate isopentenyltransferase 3, chloroplastic	*IPT3*
TRINITY_DN11300_c0_g1_i1.p1	4.6	5.6E-28	5.3	1.7E-27	cytokinin riboside 5′-monophosphate phosphoribohydrolase LOG7	*LOG7*
TRINITY_DN17801_c3_g3_i4.p1	1.1	7.5E-06	1.3	2.9E-05	histidine-containing phosphotransfer protein 1-like	*AHP1*
TRINITY_DN19500_c0_g1_i1.p1	1.5	3.7E-09	2.0	2.2E-10	adenine phosphoribosyltransferase 1-like	*APT1*
TRINITY_DN14544_c0_g1_i5.p3	3.4	6.7E-20	1.2	1.2E-04	adenine phosphoribosyltransferase 5-like	*APT5*
TRINITY_DN16686_c0_g2_i2.p1	4.2	8.3E-35	3.4	2.1E-24	cytokinin dehydrogenase 6-like	*CKX6*
TRINITY_DN12412_c0_g1_i1.p1	3.4	6.0E-34	3.8	2.9E-32	indole-3-acetic acid-amido synthetase GH3.6-like	*GH3.6*
TRINITY_DN17258_c0_g1_i1.p1	2.6	1.9E-66	1.5	3.5E-14	auxin response factor 5	*ARF5*
TRINITY_DN16500_c2_g2_i2.p1	8.4	8.7E-19	8.3	1.9E-17	AP2-like ethylene-responsive transcription factor PLT2	*PLT2*
TRINITY_DN8909_c0_g1_i1.p1	7.3	1.8E-10	3.7	2.0E-06	protein CUP-SHAPED COTYLEDON 2-like	*CUC2*
TRINITY_DN18727_c1_g1_i2.p1	3.2	2.3E-05	3.3	1.6E-04	ethylene-responsive transcription factor ESR2-like	*ESR2*
TRINITY_DN19423_c2_g6_i1.p1	1.6	2.6E-10	1.8	1.6E-09	dof zinc finger protein DOF3.4-like	*DOF3.4*
TRINITY_DN12019_c0_g1_i1.p1	1.4	1.4E-08	1.1	1.7E-04	CYCLIN-D3–1-like	*CYCD3;1*
TRINITY_DN17398_c0_g7_i1.p1	6.5	4.6E-112	7.3	2.4E-96	cytochrome P450 78A5-like	*CYP78A5*
TRINITY_DN12516_c0_g1_i1.p1	2.0	2.2E-04	3.5	2.2E-07	cytochrome P450 711A1	*CYP711A*
TRINITY_DN21545_c1_g3_i3.p1	7.1	9.0E-51	6.1	1.8E-40	LOB domain-containing protein 18	*LBD18*
TRINITY_DN20611_c3_g4_i1.p1	2.5	2.2E-39	1.2	3.6E-07	LOB domain-containing protein 21-like	*LBD21*
TRINITY_DN17808_c9_g1_i1.p1	9.2	7.4E-87	2.9	3.2E-21	LOB domain-containing protein 25	*LBD25*

^a^*FC* fold change, ^b^*FDR* false discovery rate

## Discussion

**Fig. 8:**
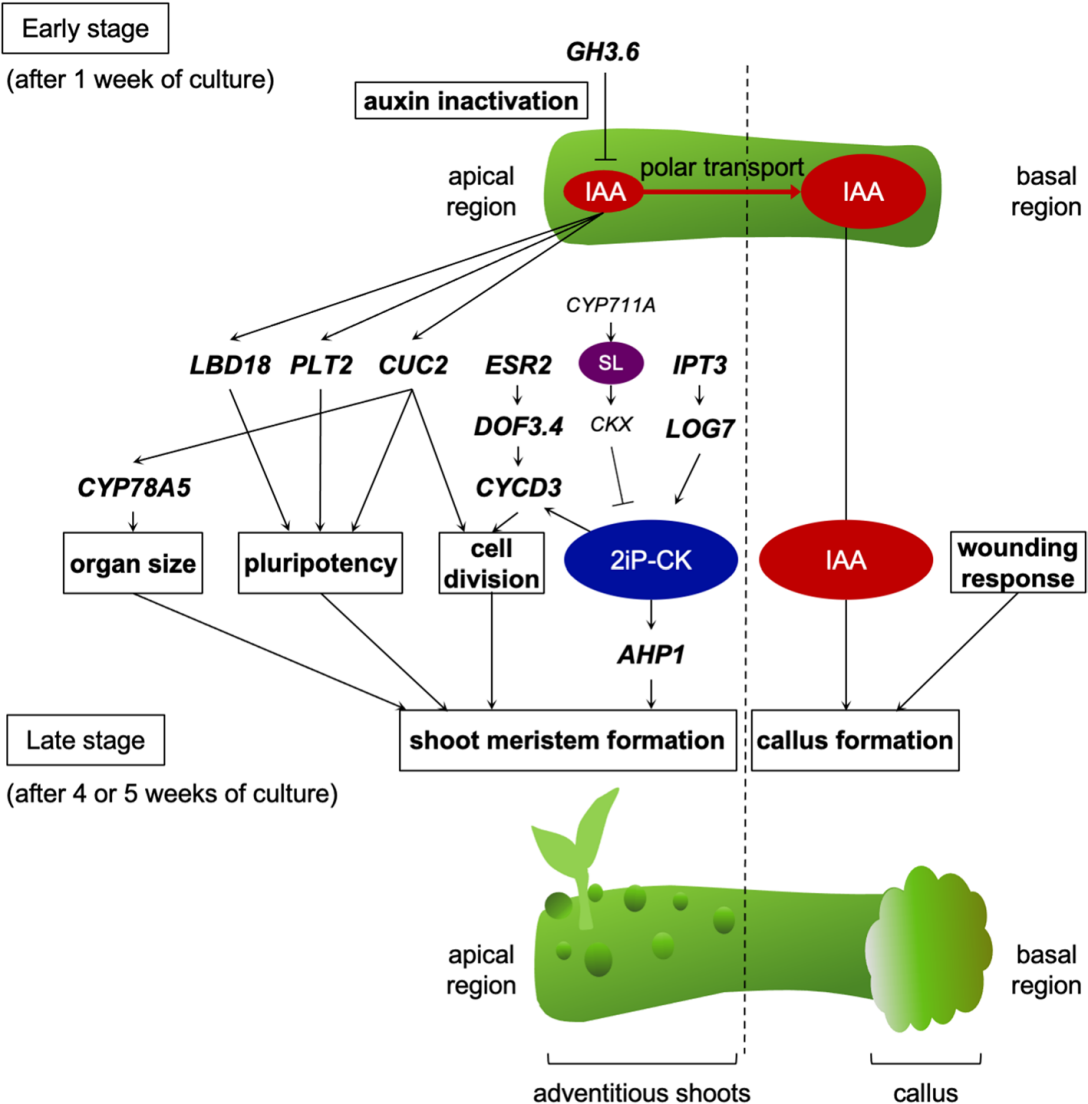
Hypothetical model of adventitious shoot formation in ipecac. In the early stage (after 1 week of culture), phytohormone biosynthesis genes, phytohormone-response genes, and shoot-formation-related genes are expressed in the apical region of internodal segments. The appropriate expression triggers the meristem formation of adventitious shoots in the late stage (after 4 or 5 weeks of culture)

We performed RNA-seq of the apical and basal regions of internodal segments of ipecac before and after 1 week of culture. Our results show that the expression of phytohormone- and organogenesis-related genes increased in the apical region of the segments after 1 week of culture (Fig. 5a), indicating that *de novo* meristem formation had already started. Here we provide a hypothetical model of gene expression at the early stage of adventitious shoot formation in ipecac based on our RNA-seq data (Fig. 8).

CK biosynthesis starts from binding of dimethyl allyl pyrophosphate and an adenine nucleotide (ATP or ADP) through IPT to form the *N*^6^-(∆^2^-isopentenyl)adenine (2iP) nucleotides [31, 32]. The isoprene side chain of 2iP nucleotides is hydroxylated by CYP735A, resulting in the formation of *trans*-zeatin (tZ) nucleotides [33]. The 2iP and tZ nucleotides become the bioactive 2iP and tZ through the action of 5′-monophosphate phosphoribohydrolase, encoded by *LOG* [34, 35]. In internode culture of ipecac in our previous work, amounts of tZ and *trans*-zeatin riboside greatly increased in the middle region of internodal segments after 1 week of culture, and tZ-type CKs were widely distributed throughout the segments [29]. Thus, we presumed that tZ-type CKs are mainly involved in the induction of ipecac adventitious shoots. However, we found CK biosynthesis genes *IPT3* and *LOG7* in the 1a-up group, but did not find *CYP735A* in our gene expression analysis (Table 2; Fig. 7), indicating that the change of 2iP-CK level, but not tZ-type CKs, has an important role in adventitious shoot formation of ipecac. For CK homeostasis, CK metabolism genes such as *APT1*, *APT5*, and *CKX6* were also induced, but their expression was relatively weak compared with that of CK biosynthesis genes (Table 2; Fig. 7), indicating the activation of CK biosynthesis, rather than CK degradation.

The 1a-up group also included an SL biosynthesis gene, *CYP711A*, which encodes an enzyme catalyzing oxidation of an SL precursor, carlactone (Table 2; Fig. 7) [36]. Elevation of *CYP711A* expression in rice produced SLs, which induced CK degradation by CKX9 [37]. Our recent study showed that exogenously applied SL suppressed adventitious shoot formation of ipecac, whereas application of SL biosynthesis inhibitors (TIS108 and KK5) or an antagonist (KK094) stimulated it by inducing biosynthesis of 2iP-type CKs [38]. Because TIS108 and KK5 both inhibited enzymatic activity of CYP711A [39, 40], reduction of SL levels might downregulate *CKX* expression, allowing endogenous CK levels to increase and thus stimulating adventitious shoot formation. This explanation also supports an idea that a change of 2iP-type CKs levels directly affects adventitious shoot formation of ipecac.

*ESR2*, *DNA-BINDING PROTEIN WITH ONE FACTOR3.4* (*DOF3.4*), and *CYCD3;1* were included in the 1a-up group (Table 2; Fig. 7). Arabidopsis has two *ESR* genes (*ESR1* and *ESR2*) encoding transcription factors belonging to the AP2/ERF family, and *ESR2* mutation decreased adventitious shoot formation more strongly than *ESR1* mutation, indicating that ESR2 has a more important role than ESR1 in regulating shoot regeneration [41]. DOF proteins are a family of plant-specific transcription factors with a conserved zinc finger DNA-binding domain. Among the DOF proteins, DOF3.4 (also named OBP1) is induced by ESR2 and can directly bind to the promoter of a D-type cyclin CYCD3;3 (probably also CYCD3;1) during G1/S transition and the action of DOF2.3 in S-phase [42, 43]. CYCD3;1 dominantly promotes the G1/S transition of the cell cycle, and expression of its gene is also regulated by CK signals in the shoot apical meristem [44, 45]. Arabidopsis has three CYCD3 members; CYCD3 is required for normal shoot meristem formation, because Arabidopsis *cycd3* triple mutants cannot form adventitious shoots on callus derived from hypocotyls cultured on CK-rich medium [46].

IAA is a major endogenous auxin and is biosynthesized by the conversion of l-Trp to indole-3-pyruvic acid by aminotransferase encoded by *TRYPTOPHAN AMINOTRANSFERASE OF ARABIDOPSIS 1 / TRYPTOPHAN AMINOTRANSFERASE RELATED*, and the conversion of indole-3-pyruvic acid to IAA through flavin monooxygenase encoded by *YUCCA* [47, 48]. In ipecac, IAA is transiently produced in the early stage (after 1 week of culture) of adventitious shoot formation [29] and induces expression of *LBD* genes, *PLT2*, and *CUC2* (Table 2; Fig. 7). Among the 43 Arabidopsis LBDs [49], *LBD18* (in particular), *LBD21*, and *LBD25* were upregulated in 1a (Table 2; Fig. 7). Both *LBD18* and *PLT2* act as regulators of root stem cells to establish pluripotency [50, 51]. However, the pluripotent cells start *de novo* shoot formation by actions of shoot-formation-promoting factors such as CUC2 and ARF5 (also named MONOPTEROS) [9, 10, 52]. *CYP78A5* (also named *KLUH*) regulates leaf initiation rate and organ size [53, 54]. Activation of CYP78A requires *CUC1* and *CUC2* expression and counteracts STM in the promotion of meristem activity [55]. *STM* and *RAP2.6 L* are also important genes activated in *de novo* shoot formation, but they are not present in 1a-up. ESR is also thought to regulate the commitment of root cells to shoot differentiation in Arabidopsis, in that ESR2 changes root stem cells induced by PLT2 into shoot meristem cells [56]. In internodal segments of ipecac, IAA is immediately transported from the apical to the basal region through PIN proteins for the homeostasis of endogenous auxin levels [30]. IAA is also inactivated by GH3, which is an acyl amidosynthetase that conjugates amino acids to IAA [57], in the apical region of the segments (Table 2; Fig. 7). Maintenance of low auxin levels in the apical region produces relatively high CK conditions, which might help adventitious shoot formation.

Furthermore, the GO analysis indicates that the biosynthesis of primary and secondary cell walls is suppressed in the apical region of internodal segments after 1 week of culture (Fig. 5b). Cellulose levels and transcripts of secondary cell wall biosynthesis genes were low in the shoot apical meristem [58]. It would be beneficial to reduce cellulose and lignin levels for adventitious meristem formation. In 1b-up, GO terms involved in immune response and wounding response were included (Fig. 6a). When plants are injured physically, they rapidly activate defense responses to protect themselves from pathogenic infections, and they repair the wound site through callus formation [18]. In ipecac, callus is formed at the wound site of the basal region of internodal segments because IAA is accumulated in the basal region [29]. Thus, detection of these GO terms in 1b-up is reasonable, but they were not found in 1a-up (Fig. 6a). In 1b-down, terms for post-embryonic plant organ morphogenesis were detected (Fig. 6b), indicating conditions in which it is difficult to form adventitious shoots.

In ipecac, adventitious shoot formation is observed in apical region of internodal segments but not in basal region. In the apical region, endogenous CK levels are increased by the expression of CK biosynthesis genes such as *IPT3* and *LOG7,* IAA inactivation is induced by *GH3* expression, and IAA is transported to basal region by polar auxin transport (Koike et al. 2020), inducing the conditions with a low auxin-to-CK ratio in the apical region. It probably triggers the expression of genes associated with shoot system morphogenesis and plant organ formation. In contrast, highly accumulated IAA suppresses CK biosynthesis and embryonic plant organ morphogenesis in the basal region, resulting in suppression of adventitious shoot formation.

## Conclusion

Ipecac can form adventitious shoots only in the apical region of internodal segments without callusing on phytohormone-free medium. We performed RNA-seq analysis to understand gene expression patterns in the early stage of *de novo* shoot regeneration. Expression patterns during adventitious shoot formation of ipecac were similar to those of shoot regeneration on callus in Arabidopsis. However, not all events showed the same patterns. We propose that CK biosynthesis, acquisition of cellular pluripotency, and initiation of cell division spontaneously start at almost the same time following wounding, and elevated endogenous CKs induce adventitious shoot formation without callusing on internodal segments. When we compare endogenous phytohormone levels in some plant species, auxin levels are lower and CK levels are higher in ipecac than in Arabidopsis, rapeseed, and tomato, which cannot form adventitious shoots without phytohormone treatment [18, 38, 59, 60]. Thus, endogenous CKs (especially 2iP-type CK) is a key regulator of adventitious shoot formation of ipecac. Although transcription of *IPT* and *LOG* is well known to be regulated in response to nitrate ion levels [61], wounding-inducible transcription factors are still unknown. In the future, it will be important to elucidate the mechanism of transcription of CK biosynthesis genes induced by wounding in ipecac.

## Materials and methods

### Plant materials and culture condition

We used the ipecac culture system established by Yoshimatsu and Shimomura [26]. Sterile plants propagated from shoot tips, nodes, and internodes were maintained at Toyo University. The explants were placed on phytohormone-free B5 culture medium (25 mL) [62] solidified with 0.2% Gelrite in a Petri dish (90 mm × 20 mm) and cultured at 24 °C under a 14-h light / 10-h dark photoperiod (10–20 μmol photons m^− 2^ s^− 1^). Internodal segments (5 mm length) were divided into two sections (apical and basal regions, 2.5 mm each) before culture (12 samples/set) or after 1 week of culture (8 samples/set) on hormone-free B5 medium for RNA-seq analysis (Fig. 1). Leaves and roots were also collected from sterile plants as reference samples. All experiments were carried out in a completely randomized design.

### RNA isolation and transcriptome sequencing

The samples for RNA-seq were frozen in liquid nitrogen and crushed with a mortar and pestle. Total RNA was isolated by using an RNeasy Plant Mini Kit (Qiagen, Hilden, Germany) following the supplied instructions. RNA quantity was measured with a Nanodrop spectrophotometer (Thermo Fisher Scientific, Waltham, MA, USA), a Qubit fluorometer (Thermo Fisher Scientific), and a 2100 Bioanalyzer (Agilent Technologies Inc., Santa Clara, CA, USA), and an RNA integrity number was determined with the 2100 Bioanalyzer. cDNA libraries were prepared according to the Low Sample Protocol of the *Illumina TruSeq Stranded mRNA Sample Preparation Guide* (Illumina, San Diego, CA, USA). RNA was diluted with RNase-free ultrapure water to 40 ng μL^− 1^. The quality of purified cDNA libraries was confirmed on the 2100 Bioanalyzer. The quantity of purified cDNA was determined by quantitative real-time PCR (qRT-PCR) according to the Kapa Library Quantification Kit platform (Illumina) using a 7500 Real-Time PCR System (Thermo Fisher Scientific). Paired-end reads (106 bp length) were sequenced on a HighSeq 1500 system (Illumina) in rapid-run mode. Sequenced reads and assembled sequences are registered with the DNA Data Bank of Japan (DDBJ).

### Analysis of differential gene expression and gene ontology (GO)

By using Cutadapt v. 2.3 software [63], low-quality sequences (quality value < 30) and adapter sequences were trimmed and reads shorter than 25 bp were filtered out. The quality of trimmed reads was evaluated with FastQC v. 0.11.9 software (http://www.bioinformatics.babraham.ac.uk/projects/fastqc/). To build a reference transcript, we performed *de novo* assembly by using Trinity v. 2.6.5 software [64, 65]. Open reading frames (ORFs) on *de novo* assembly sequences were predicted by TransDecoder. The resulting assembly was evaluated in Benchmarking Universal Single-Copy Ortholog (BUSCO) v. 3 software using the eukaryota_odb9 and embryophyta_odb9 datasets [66]. The *de novo* assembly of the transcripts is available in supplemental information. The trimmed reads were mapped to the reference transcripts with Bowtie2 v. 2.3.5.1 software [67], and the abundance was estimated with eXpress v. 1.5.1 software [68]. Differential expression was analyzed with the edgeR v. 3.26.8 package [69] in R v. 3.6.1 software using the “trimmed mean of *M* values” method [70] to calculate normalization values. We defined DEGs with log fold-change (FC) > |1| and false discovery rate (FDR) < 0.01.

GO is a unification tool used to describe the properties of an organism’s genes and their products, categorized into biological processes, cellular components, and molecular functions [71]. Sequences were functionally annotated in OmicsBox v. 1.4 Blast2GO software (BioBam, Spain, Valencia) [72]. GO enrichment analysis was performed in Metascape v. 3.5 software [73] using Arabidopsis gene IDs obtained from the Uniprot database (https://www.uniprot.org/).

### Validation for RNA-seq using quantitative real-time reverse-transcription PCR

Internodal segments (8 samples/set; Fig. 1) were placed in a 2.0-mL tube with a zirconia bead (diameter, 5 mm), frozen in liquid nitrogen, and crushed in a TissueLyser II (Qiagen). Total RNA was extracted with an RNeasy Plant Mini Kit (Qiagen) following the supplied instructions. cDNA was synthesized from 100 ng of total RNA using ReverTra Ace qPCR RT Master Mix (Toyobo, Osaka, Japan). Transcript levels were determined by qPCR using a Thunderbird NEXT SYBR qPCR Mix kit (Toyobo). qRT-PCR was performed with a StepOnePlus Real-Time PCR System (Thermo Fisher Scientific). The expression of *ELONGATION FACTOR1* (*EF1*) was used as a reference. The primer sets used are listed in Table S1. Statistical analysis of relative expression was carried out in IBM SPSS Statistics 26.0 software (IBM SPSS Inc., Armonk, NY, USA). Following assessment of the equality of variances by ANOVA, multiple comparison was conducted by Tukey’s honestly significant difference (Tukey’s HSD). *P* values less than 0.05 were considered statistically significant.

## Supplementary Information

**Additional file 1: Table S1.** Primer list for qRT-PCR. **Table S2.** Genes upregulated in apical region of internodal segments. **Table S3.** Genes downregulated in apical region of internodal segments. **Table S4.** Genes upregulated in basal region of internodal segments. **Table S5.** Genes downregulated in basal region of internodal segments.

## Data Availability

New illumina sequence reads generated during the current study are available from DDBJ Sequence Read Archive (DRA) under accession number DRA013731 (https://ddbj.nig.ac.jp/resource/sra-submission/DRA013731). Other data are included in this published article and its supplementary information files.

## Abbreviations

2iP: *N* ^6^-(∆^2^-isopentenyl)adenine
AHP: *Arabidopsis thaliana* histidine-containing phosphotransfer protein
ARF: Auxin response factor
APT: Adenine phosphoribosyltransferase
ARR: Arabidopsis response regulator
CK: Cytokinin
CKX6: Cytokinine oxidase 6
CUC: Cup-shaped cotyledon
CYCD3;1: Cyclin D3;1
DEG: Differentially expressed gene
DOF: DNA-binding protein with one factor
EF1: Elongation factor 1
ESR1: Enhancer of shoot regeneration 1
FC: Fold-change
GH3: Gretchen hagen 3
GO: Gene ontology
IAA: Indole-3-acetic acid
IPT3: Isopentenyl transferase 3
LBD: Lateral organ boundaries domain
LOG: Lonely guy
nr: Non-redundant
ORF: Open reading frame
PLT: Plethora
qRT-PCR: Quantitative real-time PCR
WUS: Wuschel
STM: Shoot meristemless
SL: Strigolactone
tZ: *Trans*-zeatin
WIND1: Wound-induced dedifferentation 1
