# Supplementary material for: Gene expression profiling before and after internode culture for adventitious shoot formation in ipecac
Source: BMC Plant Biol. 2022 Jul 22;22:361. doi: 10.1186/s12870-022-03756-w (PMC9308184; doi:10.1186/s12870-022-03756-w)
Supplement: Supplementary file 1 — Additional file 1: Table S1. Primer list for qRT-PCR. Table S2. Genes upregulated in apical region of internodal segments. Table S3. Genes downregulated in apical region of internodal segments. Table S4. Genes upregulated in basal region of internodal segments. Table S5. Genes downregulated in basal region of internodal segments. [file 12870_2022_3756_MOESM1_ESM.docx]

| **Table S1** Primer list for qRT-PCR | | |
| --- | --- | --- |
| primer name | | 5'-Sequence-3' |
| ipecac_EF1 | F* | ACGGAGCAACCAAGAGAAGA |
|  | R** | GTGGCTTCACCGATCAAGTT |
| ipecac_IPT3 | F | TCGGGGTAAGCTTCCAATCATC |
|  | R | GAATTGAAACGTCCACCCACAG |
| ipecac_LOG7 | F | TGGGTCTGATGGGTCTTGTTTC |
|  | R | ACCGGTTATCTCTCTTGGCATG |
| ipecac_AHP1 | F | GTTGAAGGGAAGCAGTTCCA |
|  | R | TCTCAAGCTTGGTCCTCACC |
| ipecac_APT1 | F | ATCCACGTATCCATGCCATT |
|  | R | GTTTTTGCCCACGTAACGAT |
| ipecac_APT5 | F | AGCCGAAACTGCAACTGATT |
|  | R | CTGGCTTTGGAAAATGAGGA |
| ipecac_CKX6 | F | CGGGTAAAGGAGAGGTGGTTAC |
|  | R | TTGTGGGAGCTGGTTCAAGAG |
| ipecac_GH3.6 | F | TATCTCCGTGCCCAAATCTC |
|  | R | AATTGAGGGGCCTTGTTCTT |
| ipecac_ARF5 | F | GGGAAATCGAAACACCTGAA |
|  | R | TGACACCGAGGGACACATAA |
| ipecac_PLT2 | F | GCTCGAGTGGGAGTTACAGC |
|  | R | GCTTGGATTTGATCCCTGAA |
| ipecac_CUC2 | F | TCCAGGGTTCCGTTTTCATCC |
|  | R | CCATGGCTCGCATTTGTTGAG |
| ipecac_ESR2 | F | AATTCGGTCAGCATGCTTCT |
|  | R | CAGAAGATTCATCCGGGAAA |
| ipecac_DOF3.4 | F | GAAAGCAGTGAACGGAAAGG |
|  | R | GGTTCCACCATGAGTCCAGT |
| ipecac_CYCD3;1 | F | AGGCTGTAGGGTGGATGTTG |
|  | R | AGGCTACCGCAGCTAATTGA |
| ipecac_CYP78A5 | F | TTCTCGCTGTTTTTGCCTTT |
|  | R | GAGTGAGGGAGCCAGTGAAG |
| ipecac_CYP711A | F | CAAAACAACCAAGCCCCTTCC |
|  | R | GCACCAAACAAGTCCGTCATC |
| ipecac_LBD18 | F | TGCATCAATCCATCACCACCTC |
|  | R | TCGCCGTTGTTGTCTTCCAG |
| ipecac_LBD21 | F | TGAAGAGCCAAGAACCTCGT |
|  | R | TTACTCGCTCCGAACACCTT |
| ipecac_LBD25 | F | GCACGCTTGAAAGATCCAGT |
|  | R | TCCCGGTTGGAAATGTATGT |
| *F: forward primer, **R: reverse primer | | |

| **Table S2** Genes upregulated in apical region of internodal segments. | | | | | |
| --- | --- | --- | --- | --- | --- |
|  | 1a/0a | | 1a/1b | |  |
|  | logFC* | FDR** | logFC* | FDR** | description |
| TRINITY_DN15503_c0_g3_i3.p1 | 1.8 | 4.5E-16 | 1.2 | 2.3E-06 | 17.8 kDa class I heat shock protein-like |
| TRINITY_DN12895_c0_g1_i1.p1 | 1.8 | 1.1E-21 | 1.0 | 3.3E-05 | 21 kDa protein-like |
| TRINITY_DN9774_c0_g1_i1.p1 | 6.7 | 2.4E-23 | 6.6 | 3.5E-20 | 3-ketoacyl-CoA synthase 11 |
| TRINITY_DN15561_c0_g9_i1.p1 | 4.1 | 5.4E-22 | 4.0 | 3.0E-22 | 3-ketoacyl-CoA synthase 11-like |
| TRINITY_DN18804_c2_g2_i2.p1 | 6.9 | 1.2E-08 | 6.9 | 5.7E-08 | 3-ketoacyl-coa synthase 6 |
| TRINITY_DN16996_c0_g6_i1.p1 | 2.8 | 7.5E-11 | 2.1 | 4.4E-07 | ABC transporter B family member 15-like |
| TRINITY_DN12560_c0_g1_i4.p1 | 4.9 | 1.6E-19 | 5.2 | 1.4E-17 | ABC transporter G family member 11-like |
| TRINITY_DN19465_c0_g2_i1.p1 | 2.5 | 5.8E-24 | 1.5 | 1.8E-08 | abnormal spindle-like microcephaly-associated protein homolog |
| TRINITY_DN14546_c0_g1_i2.p1 | 2.4 | 9.9E-12 | 1.8 | 1.8E-07 | abscisic acid 8'-hydroxylase 4-like |
| TRINITY_DN18512_c1_g7_i2.p1 | 4.5 | 9.1E-05 | 3.2 | 7.0E-03 | abscisic stress-ripening protein 1-like |
| TRINITY_DN11239_c0_g1_i1.p1 | 3.7 | 6.5E-06 | 4.9 | 1.4E-06 | acidic leucine-rich nuclear phosphoprotein 32 family member B-like |
| TRINITY_DN15073_c0_g1_i5.p2 | 3.8 | 9.8E-05 | 3.4 | 1.1E-03 | ACT domain-containing protein ACR12 isoform X1 |
| TRINITY_DN19500_c0_g1_i1.p1 | 1.5 | 3.7E-09 | 2.0 | 2.2E-10 | adenine phosphoribosyltransferase 1-like |
| TRINITY_DN14544_c0_g1_i5.p3 | 3.4 | 6.7E-20 | 1.2 | 1.2E-04 | adenine phosphoribosyltransferase 5-like |
| TRINITY_DN19677_c0_g2_i16.p1 | 2.8 | 6.2E-55 | 1.5 | 1.6E-13 | adenosine kinase |
| TRINITY_DN19677_c0_g2_i1.p1 | 2.9 | 1.1E-65 | 1.3 | 3.8E-11 | adenosine kinase |
| TRINITY_DN12309_c0_g1_i2.p1 | 3.5 | 9.3E-09 | 2.2 | 1.6E-03 | adenylate isopentenyltransferase 3, chloroplastic |
| TRINITY_DN18022_c1_g2_i19.p1 | 1.3 | 5.7E-05 | 1.0 | 7.1E-03 | allantoinase AllB |
| TRINITY_DN12731_c0_g1_i1.p1 | 1.8 | 4.5E-36 | 1.5 | 3.7E-09 | allene oxide synthase 3-like |
| TRINITY_DN19604_c0_g2_i1.p1 | 2.4 | 5.0E-40 | 1.8 | 8.1E-10 | *α*-mannosidase |
| TRINITY_DN13953_c0_g1_i1.p1 | 4.2 | 1.7E-20 | 4.3 | 4.0E-19 | ammonium transporter 1 member 1 |
| TRINITY_DN19261_c0_g2_i9.p1 | 2.3 | 1.9E-05 | 1.5 | 8.5E-03 | anaphase-promoting complex subunit 1 |
| TRINITY_DN16500_c2_g2_i2.p1 | 8.4 | 8.7E-19 | 8.3 | 1.9E-17 | AP2-like ethylene-responsive transcription factor PLT2 |
| TRINITY_DN16807_c0_g1_i1.p1 | 1.8 | 2.7E-20 | 2.3 | 2.7E-15 | aquaporin TIP1-3 |
| TRINITY_DN11001_c0_g1_i1.p2 | 1.4 | 9.9E-09 | 1.3 | 1.4E-06 | ARID/BRIGHT DNA-binding domain-containing protein |
| TRINITY_DN17961_c0_g1_i3.p1 | 1.1 | 3.3E-07 | 1.1 | 1.4E-05 | aspartic proteinase-like protein 1 |
| TRINITY_DN16820_c1_g1_i1.p1 | 2.8 | 3.3E-12 | 2.0 | 3.8E-06 | ATP-dependent DNA helicase |
| TRINITY_DN20577_c3_g1_i4.p1 | 1.5 | 3.3E-19 | 1.0 | 1.9E-06 | ATP-dependent DNA helicase DDM1 |
| TRINITY_DN17258_c0_g1_i1.p1 | 2.6 | 1.9E-66 | 1.5 | 3.5E-14 | auxin response factor 5 |
| TRINITY_DN17672_c4_g4_i1.p1 | 2.0 | 4.2E-06 | 1.5 | 7.9E-03 | auxin-responsive protein SAUR50-like |
| TRINITY_DN12615_c0_g1_i2.p1 | 3.5 | 8.2E-24 | 1.4 | 2.5E-04 | B3 domain-containing protein At3g19184-like |
| TRINITY_DN306_c0_g1_i1.p1 | 4.2 | 8.1E-04 | 5.9 | 4.5E-04 | B3 domain-containing protein Os04g0386900-like |
| TRINITY_DN16561_c0_g6_i2.p1 | 3.0 | 7.4E-03 | 6.0 | 4.0E-04 | B3 domain-containing transcription factor NGA1-like |
| TRINITY_DN10628_c0_g1_i1.p1 | 2.6 | 4.2E-06 | 7.5 | 1.2E-11 | B3 domain-containing transcription factor VRN1-like |
| TRINITY_DN19660_c0_g8_i2.p1 | 5.7 | 5.9E-04 | 5.6 | 2.4E-03 | benzyl alcohol *O*-benzoyltransferase-like |
| TRINITY_DN18916_c0_g1_i4.p1 | 5.9 | 4.6E-04 | 4.0 | 9.4E-03 | *β*-amyrin 28-oxidase-like |
| TRINITY_DN18282_c0_g1_i1.p1 | 1.6 | 5.1E-07 | 1.5 | 1.0E-05 | calcineurin-like metallo-phosphoesterase superfamily protein |
| TRINITY_DN10366_c0_g1_i1.p1 | 3.2 | 1.1E-13 | 1.3 | 5.8E-04 | carboxy-terminal domain RNA polymerase II polypeptide A small phosphatase 1-like |
| TRINITY_DN10586_c0_g1_i1.p1 | 4.4 | 5.4E-09 | 5.6 | 7.5E-10 | casein kinase 1-like protein HD16 |
| TRINITY_DN11235_c0_g1_i2.p1 | 1.1 | 6.3E-04 | 2.4 | 4.3E-05 | CASP-like protein 2A1 |
| TRINITY_DN17203_c1_g8_i2.p1 | 2.6 | 4.6E-23 | 1.9 | 1.5E-12 | cationic amino acid transporter 5 |
| TRINITY_DN19393_c1_g2_i5.p1 | 3.0 | 6.7E-21 | 1.6 | 3.3E-08 | cell division cycle-associated 7-like protein |
| TRINITY_DN15192_c0_g1_i1.p1 | 2.1 | 1.0E-17 | 1.0 | 3.6E-04 | cell division cycle-associated 7-like protein |
| TRINITY_DN12664_c0_g1_i2.p1 | 4.1 | 9.1E-16 | 3.5 | 8.2E-11 | chromatin remodeling protein EBS-like |
| TRINITY_DN11818_c0_g1_i2.p1 | 3.5 | 2.8E-06 | 2.7 | 1.7E-04 | COBRA-like protein 6 |
| TRINITY_DN19435_c0_g1_i5.p1 | 2.2 | 1.7E-11 | 1.0 | 1.8E-03 | cyclin-A1-1-like |
| TRINITY_DN12019_c0_g1_i1.p1 | 1.4 | 1.4E-08 | 1.1 | 1.7E-04 | cyclin-D3-1-like |
| TRINITY_DN20910_c3_g2_i1.p1 | 2.9 | 4.7E-10 | 1.2 | 2.9E-03 | cyclin-dependent kinase B2-1 |
| TRINITY_DN12516_c0_g1_i1.p1 | 2.0 | 2.2E-04 | 3.5 | 2.2E-07 | cytochrome P450 711A1 |
| TRINITY_DN18895_c1_g1_i2.p1 | 6.2 | 2.5E-05 | 5.6 | 1.3E-04 | cytochrome P450 71D10-like |
| TRINITY_DN17615_c2_g2_i2.p1 | 4.9 | 3.7E-60 | 6.3 | 4.5E-58 | cytochrome P450 724B1 |
| TRINITY_DN19822_c1_g2_i2.p1 | 2.1 | 1.6E-05 | 2.1 | 1.2E-05 | cytochrome P450 734A1-like |
| TRINITY_DN17398_c0_g7_i1.p1 | 6.5 | 4.6E-112 | 7.3 | 2.4E-96 | cytochrome P450 78A5-like |
| TRINITY_DN11770_c0_g2_i7.p1 | 1.2 | 8.8E-07 | 1.4 | 1.1E-04 | cytochrome P450 85A |
| TRINITY_DN14715_c0_g1_i1.p1 | 4.3 | 2.6E-27 | 1.7 | 2.2E-04 | cytochrome P450 94A2-like |
| TRINITY_DN15540_c1_g2_i3.p1 | 1.5 | 4.8E-17 | 1.0 | 4.5E-06 | cytochrome P450 CYP72A219-like |
| TRINITY_DN17174_c1_g2_i4.p1 | 1.9 | 4.2E-17 | 1.1 | 1.7E-03 | cytochrome P450 CYP736A12-like |
| TRINITY_DN16686_c0_g2_i2.p1 | 4.2 | 8.3E-35 | 3.4 | 2.1E-24 | cytokinin dehydrogenase 6-like |
| TRINITY_DN11300_c0_g1_i1.p1 | 4.6 | 5.6E-28 | 5.3 | 1.7E-27 | cytokinin riboside 5'-monophosphate phosphoribohydrolase LOG7 |
| TRINITY_DN13814_c0_g1_i6.p1 | 2.2 | 6.3E-15 | 1.5 | 9.9E-07 | DEAD-box ATP-dependent RNA helicase 37-like |
| TRINITY_DN13791_c0_g1_i5.p1 | 3.9 | 8.2E-25 | 1.5 | 1.2E-05 | denticleless protein homolog |
| TRINITY_DN20241_c1_g2_i6.p1 | 1.8 | 2.3E-13 | 1.2 | 1.3E-04 | dentin sialophosphoprotein isoform X2 |
| TRINITY_DN14871_c0_g1_i3.p1 | 1.4 | 1.3E-03 | 2.2 | 3.2E-06 | dicarboxylate transporter 2.1, chloroplastic-like |
| TRINITY_DN14680_c0_g1_i1.p1 | 7.5 | 5.4E-80 | 1.8 | 1.1E-05 | disease resistance response protein 206-like |
| TRINITY_DN17606_c2_g2_i7.p1 | 2.8 | 2.1E-30 | 1.1 | 1.3E-06 | DNA (cytosine-5)-methyltransferase CMT3 |
| TRINITY_DN17522_c0_g2_i3.p1 | 1.3 | 1.8E-07 | 1.0 | 2.7E-04 | DNA mismatch repair protein MSH2 |
| TRINITY_DN16189_c2_g1_i4.p1 | 2.5 | 1.9E-13 | 1.0 | 7.0E-03 | DNA repair protein RAD51 homolog 1 |
| TRINITY_DN19423_c2_g6_i1.p1 | 1.6 | 2.6E-10 | 1.8 | 1.6E-09 | dof zinc finger protein DOF3.4-like |
| TRINITY_DN19486_c2_g3_i3.p1 | 2.2 | 1.4E-09 | 1.3 | 1.7E-04 | dynamin-related protein 1E |
| TRINITY_DN15786_c0_g2_i1.p1 | 2.7 | 6.2E-29 | 1.0 | 1.4E-03 | dynamin-related protein 5A |
| TRINITY_DN14761_c0_g3_i2.p1 | 1.2 | 1.6E-03 | 2.5 | 2.0E-14 | endoglucanase 17 |
| TRINITY_DN19070_c0_g1_i3.p1 | 6.6 | 5.4E-07 | 6.5 | 3.4E-06 | EPIDERMAL PATTERNING FACTOR-like protein 3 |
| TRINITY_DN14195_c0_g1_i1.p1 | 5.7 | 1.2E-46 | 3.6 | 4.8E-27 | EPIDERMAL PATTERNING FACTOR-like protein 8 |
| TRINITY_DN12796_c0_g1_i1.p1 | 3.6 | 7.8E-09 | 3.5 | 7.4E-08 | EPIDERMAL PATTERNING FACTOR-like protein 9 |
| TRINITY_DN19474_c1_g6_i1.p1 | 1.6 | 2.1E-03 | 2.0 | 7.7E-04 | epoxide hydrolase 4-like |
| TRINITY_DN12847_c0_g1_i2.p1 | 1.8 | 8.1E-10 | 5.6 | 3.3E-39 | equilibrative nucleotide transporter 1 |
| TRINITY_DN12847_c0_g1_i4.p1 | 2.6 | 6.3E-04 | 4.3 | 1.9E-07 | equilibrative nucleotide transporter 1 |
| TRINITY_DN18727_c1_g1_i2.p1 | 3.2 | 2.3E-05 | 3.3 | 1.6E-04 | ethylene-responsive transcription factor ESR2-like |
| TRINITY_DN16852_c1_g10_i1.p1 | 1.6 | 4.0E-03 | 1.8 | 5.5E-03 | eukaryotic translation initiation factor 3 subunit G-like |
| TRINITY_DN18300_c3_g1_i4.p1 | 3.5 | 3.3E-21 | 1.6 | 2.4E-06 | exonuclease 1 |
| TRINITY_DN13481_c0_g1_i3.p1 | 1.4 | 1.3E-08 | 1.3 | 5.0E-05 | expansin-A1 |
| TRINITY_DN14671_c0_g1_i2.p1 | 1.1 | 6.1E-07 | 1.0 | 1.5E-04 | F-box family protein |
| TRINITY_DN14208_c0_g1_i4.p1 | 1.9 | 5.5E-26 | 1.2 | 1.4E-04 | ferritin-3, chloroplastic-like |
| TRINITY_DN15868_c0_g2_i1.p1 | 6.4 | 1.1E-40 | 1.7 | 1.5E-08 | feruloyl CoA ortho-hydroxylase 1-like |
| TRINITY_DN14116_c0_g1_i3.p1 | 3.5 | 1.1E-20 | 4.2 | 6.9E-23 | FK506-binding protein 5-like isoform X1 |
| TRINITY_DN15852_c3_g4_i2.p1 | 2.3 | 2.7E-29 | 1.2 | 1.1E-08 | formamidase |
| TRINITY_DN12340_c0_g1_i1.p1 | 3.3 | 6.7E-17 | 4.4 | 1.9E-19 | FT-interacting protein 1 |
| TRINITY_DN21036_c1_g1_i5.p1 | 2.9 | 2.8E-25 | 1.1 | 5.3E-04 | G2/mitotic-specific cyclin S13-7-like |
| TRINITY_DN19900_c0_g2_i1.p1 | 1.8 | 1.5E-06 | 1.2 | 6.6E-04 | G2/mitotic-specific cyclin S13-7-like |
| TRINITY_DN11526_c0_g1_i1.p1 | 2.1 | 1.2E-05 | 1.7 | 2.7E-03 | gallate 1-*β*-glucosyltransferase-like |
| TRINITY_DN15499_c0_g1_i1.p1 | 1.5 | 6.8E-05 | 1.5 | 4.4E-04 | *γ*-glutamyltranspeptidase 1-like |
| TRINITY_DN20585_c0_g2_i1.p1 | 4.1 | 7.1E-06 | 4.7 | 3.4E-05 | GDSL esterase/lipase EXL3-like |
| TRINITY_DN21885_c1_g1_i13.p1 | 2.0 | 1.6E-07 | 1.8 | 3.8E-09 | geraniol 8-hydroxylase-like |
| TRINITY_DN19254_c0_g2_i3.p1 | 9.0 | 2.6E-189 | 2.5 | 1.4E-12 | germin-like protein subfamily T member 2 |
| TRINITY_DN10865_c0_g1_i2.p1 | 1.3 | 8.8E-08 | 1.5 | 3.8E-09 | glucan endo-1,3-*β*-glucosidase |
| TRINITY_DN20953_c0_g1_i1.p1 | 3.3 | 1.9E-39 | 1.2 | 8.7E-06 | glucan endo-1,3-*β*-glucosidase 8-like |
| TRINITY_DN8759_c0_g1_i1.p1 | 1.5 | 2.4E-05 | 1.2 | 4.5E-03 | glutaredoxin domain-containing cysteine-rich protein 1 |
| TRINITY_DN11573_c0_g1_i2.p1 | 1.9 | 9.4E-03 | 2.6 | 2.9E-03 | glycerol-3-phosphate dehydrogenase [NAD(+)] |
| TRINITY_DN17954_c0_g5_i2.p1 | 2.5 | 7.5E-24 | 3.5 | 1.3E-29 | glycine-rich protein 23-like |
| TRINITY_DN17774_c0_g2_i3.p2 | 4.5 | 3.7E-15 | 6.4 | 2.6E-16 | glycosyl hydrolase family 35 protein |
| TRINITY_DN21803_c0_g1_i10.p1 | 4.1 | 1.2E-42 | 1.2 | 2.1E-05 | hevamine-A-like |
| TRINITY_DN14795_c0_g2_i2.p1 | 1.7 | 8.3E-11 | 1.1 | 9.8E-04 | high mobility group B protein 6-like |
| TRINITY_DN17801_c3_g3_i4.p1 | 1.1 | 7.5E-06 | 1.3 | 2.9E-05 | histidine-containing phosphotransfer protein 1-like |
| TRINITY_DN16453_c0_g1_i1.p1 | 2.9 | 3.9E-37 | 1.2 | 3.7E-05 | histone H2AX-like |
| TRINITY_DN21638_c2_g5_i1.p2 | 2.4 | 2.5E-22 | 1.1 | 1.6E-04 | histone H3 |
| TRINITY_DN20184_c0_g1_i3.p2 | 2.6 | 1.1E-25 | 1.1 | 1.2E-03 | histone H3 |
| TRINITY_DN15383_c2_g3_i1.p1 | 2.3 | 7.9E-26 | 1.1 | 1.7E-04 | homeobox protein knotted-1-like 3 isoform X2 |
| TRINITY_DN11062_c0_g1_i1.p1 | 3.3 | 2.5E-12 | 1.4 | 1.7E-03 | HTH-type transcriptional regulator |
| TRINITY_DN18127_c0_g7_i1.p1 | 3.1 | 1.2E-11 | 1.2 | 6.3E-03 | hypothetical protein G4B88_000326 |
| TRINITY_DN21536_c0_g1_i1.p1 | 1.5 | 2.4E-09 | 1.3 | 3.1E-06 | inactive LRR receptor-like serine/threonine-protein kinase BIR2 |
| TRINITY_DN13494_c0_g1_i3.p1 | 1.9 | 5.0E-09 | 1.1 | 6.3E-03 | inactive rhomboid protein |
| TRINITY_DN12412_c0_g1_i1.p1 | 3.4 | 6.0E-34 | 3.8 | 2.9E-32 | indole-3-acetic acid-amido synthetase GH3.6-like |
| TRINITY_DN19157_c2_g1_i1.p1 | 2.3 | 8.5E-28 | 1.6 | 9.3E-14 | inositol-3-phosphate synthase |
| TRINITY_DN16034_c0_g8_i1.p1 | 1.9 | 7.9E-18 | 1.2 | 3.2E-05 | kinesin-like protein KIN-10A |
| TRINITY_DN18998_c1_g1_i4.p1 | 2.2 | 1.7E-11 | 1.1 | 3.9E-04 | kinesin-like protein KIN-10B |
| TRINITY_DN19330_c4_g1_i8.p1 | 2.6 | 3.2E-10 | 1.1 | 2.8E-04 | kinesin-like protein KIN-10C isoform X2 |
| TRINITY_DN19764_c0_g1_i6.p1 | 2.5 | 9.4E-34 | 1.1 | 9.0E-06 | kinesin-like protein KIN-12E |
| TRINITY_DN12747_c0_g1_i2.p1 | 1.4 | 5.5E-10 | 1.2 | 4.1E-05 | kinesin-like protein KIN-14R |
| TRINITY_DN19732_c0_g2_i7.p1 | 2.1 | 2.7E-20 | 1.1 | 4.3E-04 | kinesin-like protein KIN-5C |
| TRINITY_DN20904_c1_g1_i1.p1 | 2.4 | 4.1E-14 | 1.1 | 4.2E-04 | kinesin-like protein KIN-6 isoform X1 |
| TRINITY_DN17951_c0_g2_i1.p1 | 1.6 | 1.0E-08 | 1.0 | 6.9E-03 | kinesin-like protein NACK1 |
| TRINITY_DN16558_c5_g1_i9.p1 | 2.1 | 2.9E-11 | 1.0 | 5.0E-03 | kinetochore-associated protein KNL-2 homolog |
| TRINITY_DN21135_c0_g1_i12.p1 | 2.2 | 3.5E-05 | 2.1 | 2.9E-06 | kirola-like |
| TRINITY_DN15067_c0_g2_i1.p1 | 9.0 | 1.7E-31 | 1.2 | 2.8E-03 | kiwellin-like |
| TRINITY_DN11055_c0_g1_i1.p1 | 6.2 | 5.7E-17 | 2.6 | 1.4E-07 | lanC-like protein GCL2 |
| TRINITY_DN18444_c0_g1_i4.p1 | 1.7 | 2.1E-05 | 1.8 | 3.2E-05 | late embryogenesis abundant protein, LEA-14 |
| TRINITY_DN21003_c0_g3_i3.p1 | 2.1 | 1.4E-17 | 1.1 | 3.0E-04 | leucine-rich repeat-containing protein 50 |
| TRINITY_DN21545_c1_g3_i3.p1 | 7.1 | 9.0E-51 | 6.1 | 1.8E-40 | LOB domain-containing protein 18 |
| TRINITY_DN20611_c3_g4_i1.p1 | 2.5 | 2.2E-39 | 1.2 | 3.6E-07 | LOB domain-containing protein 21-like |
| TRINITY_DN17808_c9_g1_i1.p1 | 9.2 | 7.4E-87 | 2.9 | 3.2E-21 | LOB domain-containing protein 25 |
| TRINITY_DN19516_c3_g2_i1.p1 | 2.4 | 1.4E-30 | 2.7 | 8.8E-29 | LRR receptor-like serine/threonine-protein kinase ERL1 |
| TRINITY_DN19516_c3_g2_i9.p1 | 1.6 | 9.9E-05 | 2.8 | 7.7E-04 | LRR receptor-like serine/threonine-protein kinase ERL1 |
| TRINITY_DN18790_c1_g1_i1.p1 | 2.9 | 5.7E-28 | 1.6 | 1.9E-07 | lysine-specific demethylase JMJ25-like |
| TRINITY_DN12169_c0_g1_i1.p1 | 2.4 | 1.1E-09 | 3.0 | 7.6E-06 | metallothionein-like protein type 3 |
| TRINITY_DN17698_c0_g7_i1.p1 | 1.7 | 2.5E-13 | 1.4 | 1.5E-07 | micronuclear linker histone polyprotein-like protein |
| TRINITY_DN18318_c0_g2_i1.p1 | 2.4 | 1.7E-20 | 1.2 | 1.3E-06 | microtubule-associated protein TORTIFOLIA1 |
| TRINITY_DN11116_c0_g1_i1.p1 | 6.7 | 3.0E-26 | 1.5 | 2.8E-04 | miraculin-like |
| TRINITY_DN10614_c0_g1_i2.p1 | 1.0 | 3.8E-04 | 3.0 | 6.8E-22 | MLP-like protein 423 |
| TRINITY_DN15352_c0_g2_i1.p1 | 5.2 | 3.1E-13 | 4.3 | 3.6E-11 | ---NA***--- |
| TRINITY_DN16665_c0_g4_i1.p1 | 1.3 | 6.7E-06 | 1.7 | 5.7E-10 | ---NA--- |
| TRINITY_DN21963_c1_g3_i1.p1 | 1.7 | 1.5E-15 | 1.5 | 1.7E-09 | ---NA--- |
| TRINITY_DN18003_c1_g1_i2.p1 | 8.8 | 3.3E-45 | 1.7 | 7.0E-07 | ---NA--- |
| TRINITY_DN21545_c1_g3_i2.p1 | 6.7 | 2.8E-07 | 4.8 | 1.1E-05 | ---NA--- |
| TRINITY_DN20444_c0_g5_i4.p1 | 1.8 | 6.4E-07 | 1.5 | 2.6E-05 | ---NA--- |
| TRINITY_DN10341_c0_g1_i1.p2 | 1.9 | 5.1E-04 | 2.6 | 3.7E-05 | ---NA--- |
| TRINITY_DN20048_c1_g1_i4.p1 | 1.8 | 2.1E-04 | 2.0 | 3.0E-04 | ---NA--- |
| TRINITY_DN17599_c3_g4_i2.p1 | 3.0 | 1.0E-03 | 3.8 | 1.0E-03 | ---NA--- |
| TRINITY_DN20444_c0_g5_i1.p1 | 2.4 | 5.1E-05 | 1.8 | 4.9E-03 | ---NA--- |
| TRINITY_DN4657_c0_g1_i1.p1 | 4.3 | 2.1E-03 | 4.2 | 6.4E-03 | ---NA--- |
| TRINITY_DN18415_c0_g3_i3.p1 | 1.7 | 5.7E-08 | 1.2 | 4.9E-05 | nascent polypeptide-associated complex subunit *α*, muscle-specific form-like |
| TRINITY_DN19630_c3_g3_i7.p1 | 1.3 | 1.9E-04 | 2.4 | 1.2E-12 | nitrate regulatory gene2 protein-like |
| TRINITY_DN16955_c0_g4_i2.p1 | 2.3 | 1.2E-26 | 2.4 | 4.5E-21 | non-specific lipid-transfer protein 1-like |
| TRINITY_DN16955_c0_g4_i7.p1 | 2.4 | 4.0E-28 | 2.3 | 1.0E-10 | non-specific lipid-transfer protein 1-like |
| TRINITY_DN19693_c1_g3_i12.p1 | 1.7 | 2.3E-14 | 1.1 | 2.9E-06 | non-specific lipid-transfer protein-like protein At5g64080 |
| TRINITY_DN12134_c0_g1_i1.p1 | 2.6 | 3.5E-34 | 1.3 | 1.0E-07 | *ω*-hydroxypalmitate *O*-feruloyl transferase |
| TRINITY_DN21195_c0_g2_i17.p1 | 1.2 | 1.9E-04 | 2.8 | 2.9E-26 | patatin-like protein 2 |
| TRINITY_DN9270_c0_g1_i1.p1 | 2.3 | 8.6E-04 | 2.2 | 6.0E-03 | patellin-4 |
| TRINITY_DN21417_c0_g2_i2.p1 | 6.0 | 1.4E-35 | 3.6 | 9.0E-16 | peroxidase 10-like |
| TRINITY_DN12229_c0_g1_i1.p1 | 5.3 | 5.0E-08 | 7.0 | 2.7E-08 | peroxidase 46 |
| TRINITY_DN17205_c2_g1_i12.p1 | 2.1 | 3.8E-18 | 1.5 | 2.9E-08 | phosphatidylinositol 3,4,5-trisphosphate 3-phosphatase and protein-tyrosine-phosphatase PTEN2A-like |
| TRINITY_DN13294_c0_g1_i1.p1 | 2.3 | 6.3E-24 | 1.0 | 1.8E-05 | phosphatidylinositol 4-kinase *γ* 4-like |
| TRINITY_DN17676_c0_g1_i1.p1 | 1.3 | 9.3E-06 | 1.0 | 1.8E-03 | phosphatidylinositol/phosphatidylcholine transfer protein SFH11 |
| TRINITY_DN17978_c1_g4_i2.p1 | 1.8 | 4.7E-20 | 2.3 | 2.3E-23 | phospholipase A2-*α* |
| TRINITY_DN15352_c0_g1_i3.p1 | 9.0 | 4.3E-112 | 5.8 | 4.8E-66 | phylloplanin-like |
| TRINITY_DN20363_c1_g1_i2.p1 | 2.5 | 6.0E-11 | 1.6 | 2.9E-04 | PLASMODESMATA CALLOSE-BINDING PROTEIN 3-like |
| TRINITY_DN12441_c0_g1_i4.p1 | 4.6 | 3.5E-17 | 5.9 | 1.3E-17 | PLATZ transcription factor family protein |
| TRINITY_DN10924_c0_g1_i3.p1 | 4.9 | 6.8E-10 | 1.7 | 5.4E-03 | PLATZ transcription factor family protein |
| TRINITY_DN14809_c0_g2_i1.p1 | 1.3 | 1.4E-03 | 1.6 | 1.1E-03 | polyamine oxidase 1 |
| TRINITY_DN6024_c0_g1_i1.p1 | 3.7 | 9.3E-05 | 4.1 | 7.8E-05 | polygalacturonase At1g48100-like |
| TRINITY_DN10446_c0_g1_i1.p1 | 10.3 | 6.6E-67 | 8.3 | 2.7E-57 | polyvinylalcohol dehydrogenase-like |
| TRINITY_DN15132_c0_g1_i1.p1 | 4.3 | 4.8E-09 | 4.2 | 2.0E-08 | probable acyl-activating enzyme 6 |
| TRINITY_DN21203_c0_g2_i2.p1 | 1.3 | 6.2E-05 | 1.3 | 1.7E-04 | probable alkaline/neutral invertase B |
| TRINITY_DN12087_c0_g1_i2.p1 | 2.5 | 6.3E-36 | 1.6 | 1.1E-13 | probable carboxylesterase 12 |
| TRINITY_DN18646_c1_g1_i2.p1 | 2.4 | 1.2E-19 | 1.0 | 6.2E-04 | probable DNA primase large subunit |
| TRINITY_DN17601_c0_g2_i5.p1 | 6.9 | 1.1E-18 | 3.5 | 4.4E-11 | probable LRR receptor-like serine/threonine-protein kinase At3g47570 |
| TRINITY_DN21629_c1_g1_i11.p1 | 1.3 | 2.4E-08 | 1.3 | 5.2E-05 | probable LRR receptor-like serine/threonine-protein kinase At3g47570 |
| TRINITY_DN13878_c0_g1_i1.p1 | 2.3 | 4.9E-11 | 1.9 | 2.4E-06 | probable LRR receptor-like serine/threonine-protein kinase At4g36180 |
| TRINITY_DN20990_c1_g3_i1.p1 | 3.0 | 3.2E-12 | 1.9 | 9.2E-06 | probable membrane-associated kinase regulator 2 |
| TRINITY_DN17980_c0_g3_i4.p1 | 1.9 | 1.5E-06 | 1.8 | 1.8E-04 | probable methyltransferase PMT27 |
| TRINITY_DN17493_c1_g6_i2.p1 | 2.8 | 7.0E-09 | 4.9 | 1.3E-13 | probable serine/threonine-protein kinase PBL7 |
| TRINITY_DN13881_c0_g1_i1.p1 | 1.3 | 6.9E-06 | 1.1 | 5.7E-03 | probable transcription factor PosF21 |
| TRINITY_DN14965_c0_g1_i1.p1 | 1.7 | 1.1E-04 | 1.6 | 1.7E-07 | probable WRKY transcription factor 71 |
| TRINITY_DN15044_c0_g1_i1.p1 | 2.1 | 1.8E-04 | 1.2 | 6.8E-04 | probably inactive leucine-rich repeat receptor-like protein kinase IMK2 |
| TRINITY_DN21306_c1_g1_i1.p1 | 3.4 | 2.6E-18 | 1.6 | 5.5E-07 | proliferating cell nuclear antigen |
| TRINITY_DN15843_c0_g1_i4.p1 | 1.4 | 1.6E-09 | 1.1 | 9.4E-07 | proline dehydrogenase 2, mitochondrial-like |
| TRINITY_DN21811_c0_g6_i1.p1 | 2.0 | 6.1E-11 | 1.5 | 2.6E-08 | protein CHUP1, chloroplastic |
| TRINITY_DN8909_c0_g1_i1.p1 | 7.3 | 1.8E-10 | 3.7 | 2.0E-06 | protein CUP-SHAPED COTYLEDON 2-like |
| TRINITY_DN14244_c0_g1_i1.p1 | 2.5 | 3.7E-20 | 1.1 | 5.8E-05 | protein CYPRO4 |
| TRINITY_DN16750_c0_g1_i8.p1 | 1.7 | 8.1E-05 | 1.1 | 2.9E-03 | protein DETOXIFICATION 29-like |
| TRINITY_DN13102_c0_g2_i1.p1 | 1.5 | 3.7E-04 | 3.2 | 1.1E-09 | protein DETOXIFICATION 48-like |
| TRINITY_DN19915_c1_g4_i1.p1 | 2.9 | 1.2E-10 | 6.0 | 1.3E-31 | protein ECERIFERUM 1-like |
| TRINITY_DN11937_c0_g1_i1.p1 | 7.9 | 1.0E-17 | 1.4 | 5.0E-03 | protein ECERIFERUM 1-like |
| TRINITY_DN9739_c0_g1_i1.p1 | 3.4 | 9.5E-20 | 1.7 | 1.6E-06 | protein FANTASTIC FOUR 3-like |
| TRINITY_DN19168_c1_g1_i1.p1 | 3.6 | 9.5E-22 | 1.5 | 3.0E-05 | protein IQ-DOMAIN 1-like |
| TRINITY_DN16562_c0_g5_i3.p1 | 2.5 | 4.3E-05 | 3.7 | 3.1E-06 | protein JASON-like |
| TRINITY_DN18981_c0_g4_i3.p1 | 2.2 | 1.6E-14 | 1.4 | 6.6E-06 | protein neuralized |
| TRINITY_DN10321_c0_g1_i1.p1 | 1.8 | 8.0E-12 | 1.4 | 3.5E-06 | protein NUCLEAR FUSION DEFECTIVE 4-like |
| TRINITY_DN14213_c0_g1_i4.p1 | 1.8 | 5.0E-16 | 4.3 | 1.4E-41 | protein RICE SALT SENSITIVE 3-like |
| TRINITY_DN20124_c0_g5_i1.p1 | 5.7 | 4.9E-13 | 5.6 | 1.2E-10 | protein SHI RELATED SEQUENCE 1-like |
| TRINITY_DN16892_c0_g5_i1.p1 | 1.9 | 2.9E-06 | 1.7 | 3.1E-09 | protein SHI RELATED SEQUENCE 1-like |
| TRINITY_DN20284_c0_g3_i3.p1 | 2.3 | 3.0E-14 | 5.3 | 8.6E-29 | protein STICHEL-like 2 |
| TRINITY_DN17004_c1_g2_i1.p1 | 4.0 | 2.3E-11 | 4.6 | 6.8E-11 | protein STICHEL-like 2 |
| TRINITY_DN18014_c2_g1_i2.p1 | 5.1 | 2.2E-30 | 4.8 | 1.6E-26 | protein terminal ear1 homolog |
| TRINITY_DN4694_c0_g1_i1.p1 | 6.0 | 8.5E-05 | 3.3 | 1.0E-02 | protein terminal ear1 homolog |
| TRINITY_DN16404_c1_g2_i14.p1 | 3.1 | 3.7E-22 | 1.2 | 1.6E-03 | protein TPX2-like isoform X1 |
| TRINITY_DN15615_c0_g1_i4.p1 | 2.4 | 2.2E-12 | 1.2 | 4.5E-05 | putative B3 domain-containing protein At5g58280 |
| TRINITY_DN17651_c1_g2_i1.p1 | 1.4 | 2.4E-05 | 1.1 | 5.2E-04 | putative cell division cycle ATPase |
| TRINITY_DN14948_c0_g1_i2.p1 | 6.6 | 1.1E-125 | 2.3 | 8.2E-23 | putative DNAJ domain-containing protein |
| TRINITY_DN16211_c0_g2_i2.p1 | 1.2 | 1.2E-09 | 1.4 | 4.3E-10 | putative E3 ubiquitin-protein ligase LIN-1 |
| TRINITY_DN17327_c0_g3_i1.p1 | 4.9 | 2.5E-25 | 2.6 | 2.9E-11 | putative F-box/LRR-repeat protein At5g54820 |
| TRINITY_DN12665_c0_g1_i2.p1 | 3.3 | 1.9E-06 | 3.9 | 1.8E-06 | putative galactose-binding domain-containing protein |
| TRINITY_DN19944_c1_g1_i4.p1 | 5.0 | 3.8E-12 | 2.0 | 3.2E-04 | putative germin-like protein 2-1 |
| TRINITY_DN20463_c0_g3_i1.p1 | 2.3 | 3.4E-20 | 1.0 | 5.8E-04 | putative glycosyltransferase 7 |
| TRINITY_DN19182_c0_g1_i8.p1 | 3.0 | 3.5E-24 | 2.2 | 1.3E-11 | putative hydroxyproline-rich glycofamily protein |
| TRINITY_DN17564_c0_g2_i2.p1 | 3.4 | 4.2E-44 | 1.1 | 2.4E-07 | putative SWI/SNF-related matrix-associated actin-dependent regulator of chromatin subfamily A member 3-like 3 |
| TRINITY_DN14425_c0_g1_i2.p1 | 3.1 | 4.2E-17 | 5.3 | 2.3E-27 | receptor-like protein kinase 2 |
| TRINITY_DN20223_c1_g6_i2.p1 | 1.4 | 5.9E-06 | 2.0 | 2.3E-10 | reverse transcriptase |
| TRINITY_DN20541_c6_g2_i1.p1 | 1.2 | 1.1E-03 | 2.2 | 1.6E-18 | ribonuclease H |
| TRINITY_DN21785_c2_g2_i4.p1 | 1.2 | 8.1E-06 | 1.2 | 9.3E-05 | ribosomal protein L34Ae |
| TRINITY_DN9771_c0_g1_i1.p1 | 2.7 | 3.9E-17 | 1.2 | 5.7E-04 | RNA polymerase II elongation factor |
| TRINITY_DN21191_c0_g2_i1.p1 | 2.4 | 1.3E-09 | 1.9 | 4.8E-08 | *S*-adenosylmethionine decarboxylase proenzyme-like |
| TRINITY_DN10798_c0_g1_i4.p1 | 2.5 | 5.8E-24 | 1.3 | 6.9E-07 | secoisolariciresinol dehydrogenase-like |
| TRINITY_DN16542_c0_g2_i1.p1 | 2.5 | 3.1E-12 | 1.5 | 2.5E-06 | serine carboxypeptidase-like 25 |
| TRINITY_DN20560_c0_g5_i2.p1 | 2.1 | 1.1E-13 | 1.0 | 3.3E-04 | serine/arginine repetitive matrix protein 1 |
| TRINITY_DN13782_c0_g1_i1.p1 | 1.8 | 5.4E-08 | 1.5 | 7.3E-04 | serine/arginine repetitive matrix protein 1-like |
| TRINITY_DN18058_c0_g2_i1.p1 | 2.2 | 2.7E-12 | 1.0 | 2.9E-03 | serine/arginine repetitive matrix protein 2-like |
| TRINITY_DN13674_c0_g1_i2.p1 | 2.5 | 1.2E-12 | 1.0 | 2.0E-03 | serine/threonine-protein kinase aurora-1 |
| TRINITY_DN16080_c0_g1_i2.p1 | 3.5 | 1.1E-12 | 4.4 | 1.4E-15 | serine/threonine-protein kinase TAO3 |
| TRINITY_DN20810_c0_g1_i1.p1 | 3.2 | 8.1E-11 | 2.8 | 2.3E-08 | serine/threonine-protein kinase-like protein ACR4 |
| TRINITY_DN13071_c0_g1_i1.p1 | 8.2 | 6.4E-21 | 6.3 | 7.1E-18 | small ubiquitin-related modifier 1-like |
| TRINITY_DN17017_c2_g3_i1.p1 | 1.4 | 1.6E-06 | 1.4 | 2.9E-06 | sodium/hydrogen exchanger 2-like |
| TRINITY_DN15769_c0_g1_i4.p1 | 6.0 | 9.5E-61 | 1.4 | 4.3E-05 | S-type anion channel SLAH2-like |
| TRINITY_DN21938_c2_g5_i1.p1 | 4.3 | 1.5E-05 | 3.3 | 5.5E-04 | subtilisin-like protease SBT1.9 |
| TRINITY_DN14480_c0_g2_i2.p1 | 2.4 | 6.0E-17 | 2.3 | 1.9E-15 | subtilisin-like protease SBT5.3 |
| TRINITY_DN13692_c0_g1_i2.p1 | 2.7 | 1.6E-08 | 1.4 | 5.8E-06 | sulfite exporter TauE/SafE family protein 3-like |
| TRINITY_DN18109_c2_g3_i2.p1 | 1.2 | 4.6E-03 | 1.4 | 2.9E-05 | tetratricopeptide-like helical |
| TRINITY_DN13877_c0_g1_i1.p1 | 1.4 | 6.6E-03 | 3.0 | 3.7E-08 | TIP41-like protein |
| TRINITY_DN14456_c0_g5_i1.p1 | 1.5 | 9.5E-10 | 1.2 | 1.2E-06 | titin homolog |
| TRINITY_DN9030_c0_g1_i1.p1 | 3.6 | 1.9E-21 | 4.2 | 1.2E-22 | titin-like isoform X2 |
| TRINITY_DN18988_c1_g5_i1.p1 | 2.6 | 3.8E-04 | 3.4 | 3.4E-05 | transcription factor bHLH18-like |
| TRINITY_DN18988_c1_g2_i1.p1 | 1.1 | 8.3E-04 | 1.3 | 1.5E-04 | transcription factor bHLH18-like |
| TRINITY_DN17410_c0_g1_i2.p1 | 2.3 | 2.6E-17 | 1.3 | 1.3E-06 | transcription factor BIM1 isoform X1 |
| TRINITY_DN21327_c1_g1_i1.p1 | 6.5 | 1.3E-06 | 4.6 | 5.0E-05 | transcription factor CSA-like |
| TRINITY_DN20497_c0_g2_i1.p1 | 2.1 | 3.4E-10 | 2.1 | 2.2E-08 | transcription factor HBP-1b(c38)-like isoform X1 |
| TRINITY_DN11518_c0_g1_i1.p1 | 1.6 | 1.3E-04 | 1.5 | 1.3E-03 | transcription factor IBH1-like |
| TRINITY_DN10342_c0_g1_i1.p1 | 5.0 | 7.2E-16 | 2.7 | 7.3E-08 | transcription factor TCP12-like |
| TRINITY_DN20952_c1_g2_i1.p1 | 3.6 | 5.6E-17 | 2.6 | 5.9E-11 | transcription factor TCP4-like |
| TRINITY_DN17568_c1_g6_i1.p1 | 2.5 | 2.9E-07 | 3.1 | 2.9E-09 | transcription repressor OFP12-like |
| TRINITY_DN16478_c1_g3_i1.p1 | 2.7 | 9.2E-07 | 3.4 | 1.5E-07 | transcription repressor OFP6-like |
| TRINITY_DN2300_c0_g1_i1.p1 | 1.6 | 1.6E-05 | 1.6 | 8.0E-05 | transcription repressor OFP7-like |
| TRINITY_DN13765_c0_g1_i2.p1 | 2.9 | 1.0E-21 | 1.1 | 4.2E-04 | transcriptional adapter ADA2b |
| TRINITY_DN12660_c0_g1_i4.p1 | 2.3 | 1.1E-26 | 1.0 | 8.3E-05 | transmembrane protein |
| TRINITY_DN20184_c0_g5_i1.p1 | 3.2 | 4.8E-42 | 1.0 | 1.2E-03 | transmembrane protein |
| TRINITY_DN20572_c1_g2_i1.p1 | 2.3 | 2.3E-07 | 3.3 | 3.5E-41 | trans-resveratrol di-*O*-methyltransferase-like |
| TRINITY_DN17164_c0_g1_i1.p1 | 3.6 | 3.4E-24 | 3.0 | 8.5E-16 | trans-resveratrol di-*O*-methyltransferase-like |
| TRINITY_DN18477_c0_g4_i2.p1 | 3.8 | 2.5E-19 | 2.9 | 1.7E-06 | trans-resveratrol di-*O*-methyltransferase-like |
| TRINITY_DN14618_c0_g1_i2.p1 | 1.8 | 1.4E-12 | 1.6 | 3.3E-08 | trihelix transcription factor PTL-like |
| TRINITY_DN13961_c0_g2_i1.p1 | 1.4 | 6.4E-04 | 2.7 | 1.5E-07 | two-component response regulator-like APRR5 |
| TRINITY_DN20803_c0_g9_i1.p1 | 2.0 | 6.1E-13 | 2.5 | 1.1E-13 | UDP-glycosyltransferase 87A1-like |
| TRINITY_DN14932_c0_g1_i4.p1 | 1.9 | 6.8E-03 | 2.5 | 2.3E-03 | uncharacterized LOC101253060 |
| TRINITY_DN21697_c1_g1_i2.p2 | 2.6 | 1.1E-11 | 1.5 | 2.9E-04 | uncharacterized protein LOC113715625 |
| TRINITY_DN19763_c0_g2_i1.p1 | 2.2 | 2.6E-14 | 2.3 | 1.3E-11 | uncharacterized serine-rich protein C215.13-like |
| TRINITY_DN10606_c0_g1_i2.p1 | 3.1 | 6.2E-16 | 2.8 | 1.1E-11 | unnamed protein product |
| TRINITY_DN10391_c0_g1_i1.p1 | 2.1 | 8.7E-08 | 1.5 | 2.4E-03 | UvrABC system protein C |
| TRINITY_DN18286_c0_g2_i6.p1 | 2.9 | 1.0E-10 | 2.3 | 1.6E-12 | vacuolar cation/proton exchanger 3-like |
| TRINITY_DN18850_c2_g1_i12.p1 | 2.0 | 3.7E-23 | 1.6 | 2.1E-11 | VAN3-binding protein |
| TRINITY_DN16860_c1_g7_i1.p1 | 2.0 | 1.0E-14 | 1.8 | 2.3E-08 | VAN3-binding protein-like |
| TRINITY_DN15010_c0_g1_i1.p1 | 1.1 | 3.4E-04 | 1.2 | 5.9E-03 | VIN3-like protein 2 |
| TRINITY_DN20351_c0_g4_i4.p1 | 2.4 | 4.8E-05 | 8.2 | 9.2E-17 | vinorine synthase-like |
| TRINITY_DN10721_c0_g1_i2.p1 | 3.1 | 5.1E-14 | 6.2 | 4.6E-23 | VOC family protein |
| TRINITY_DN15829_c0_g1_i4.p1 | 2.6 | 3.5E-23 | 1.0 | 3.7E-03 | wee1-like protein kinase |
| TRINITY_DN16854_c0_g1_i1.p1 | 3.0 | 1.4E-20 | 1.1 | 5.9E-04 | zinc finger CCCH domain-containing protein 62-like |
| TRINITY_DN13154_c0_g1_i2.p1 | 2.5 | 5.2E-10 | 3.4 | 2.6E-12 | zinc finger protein 6-like |
| TRINITY_DN9325_c0_g1_i1.p1 | 6.8 | 4.1E-08 | 2.9 | 4.3E-04 | zinc finger protein WIP2-like |
| TRINITY_DN17743_c1_g2_i1.p1 | 1.8 | 4.2E-10 | 1.8 | 4.3E-08 | zinc-finger homeodomain protein 6-like |
| TRINITY_DN21706_c2_g1_i1.p1 | 3.3 | 1.1E-32 | 2.1 | 1.0E-12 | zinc-finger homeodomain protein 9-like |

*FC: fold change, **FDR: false discovery rate, ***NA: not available

| **Table S3** Genes downregulated in apical region of internodal segments. | | | | | |
| --- | --- | --- | --- | --- | --- |
|  | 1a/0a | | 1a/1b | |  |
|  | logFC* | FDR** | logFC* | FDR** | description |
| TRINITY_DN19246_c2_g4_i2.p1 | -3.8 | 3.5E-40 | -2.9 | 1.3E-17 | (*R*,*S*)-reticuline 7-O-methyltransferase-like |
| TRINITY_DN12500_c0_g1_i1.p1 | -1.8 | 4.8E-09 | -1.1 | 7.1E-03 | 17.3 kDa class I heat shock protein-like |
| TRINITY_DN13476_c0_g1_i1.p1 | -1.9 | 5.5E-03 | -2.4 | 1.9E-05 | 1-aminocyclopropane-1-carboxylate oxidase |
| TRINITY_DN4762_c0_g1_i1.p1 | -1.6 | 2.8E-05 | -1.2 | 3.7E-04 | 21 kDa protein-like |
| TRINITY_DN15720_c3_g4_i2.p1 | -2.7 | 2.0E-34 | -2.1 | 4.9E-11 | 3-*epi*-6-deoxocathasterone 23-monooxygenase |
| TRINITY_DN21788_c1_g2_i1.p1 | -1.5 | 4.6E-05 | -1.4 | 1.1E-03 | 3-ketoacyl-CoA synthase 1 |
| TRINITY_DN13905_c0_g1_i3.p1 | -2.2 | 3.8E-09 | -2.1 | 6.9E-08 | 65-kDa microtubule-associated protein 8 |
| TRINITY_DN19402_c2_g2_i4.p1 | -1.2 | 9.2E-04 | -2.0 | 8.7E-14 | AAA-ATPase ASD, mitochondrial-like |
| TRINITY_DN19402_c2_g2_i1.p1 | -1.9 | 4.5E-05 | -2.4 | 1.6E-10 | AAA-ATPase ASD, mitochondrial-like |
| TRINITY_DN15309_c0_g1_i2.p1 | -1.1 | 1.3E-05 | -1.2 | 1.0E-04 | AAA-ATPase At3g50940-like |
| TRINITY_DN16478_c1_g2_i4.p1 | -6.8 | 2.7E-43 | -4.3 | 1.2E-12 | abscisic acid 8'-hydroxylase 3-like |
| TRINITY_DN17096_c3_g3_i1.p1 | -3.2 | 1.9E-55 | -1.3 | 2.3E-04 | abscisic stress-ripening protein 2-like |
| TRINITY_DN10936_c0_g1_i1.p1 | -3.3 | 8.6E-12 | -2.1 | 6.4E-03 | activity-dependent neuroprotector homeobox protein |
| TRINITY_DN16711_c0_g2_i2.p1 | -1.5 | 2.6E-06 | -1.7 | 4.1E-06 | acyl transferase 4 |
| TRINITY_DN19789_c0_g2_i4.p1 | -1.4 | 6.7E-06 | -1.1 | 2.8E-03 | alcohol dehydrogenase-like 7 |
| TRINITY_DN21146_c0_g4_i1.p1 | -3.2 | 5.4E-44 | -1.3 | 1.4E-04 | alkane hydroxylase MAH1-like |
| TRINITY_DN14299_c0_g1_i2.p1 | -1.5 | 4.0E-07 | -1.3 | 4.5E-03 | *α*-L-fucosidase 1 |
| TRINITY_DN17329_c0_g1_i3.p1 | -1.1 | 5.4E-07 | -1.3 | 1.7E-06 | amino acid permease 6-like |
| TRINITY_DN19039_c1_g8_i1.p1 | -1.4 | 2.2E-07 | -1.3 | 2.7E-05 | anthocyanidin 3-*O*-glucosyltransferase 2-like |
| TRINITY_DN9625_c0_g1_i1.p1 | -1.6 | 5.3E-09 | -1.2 | 1.2E-03 | anthocyanidin 3-*O*-glucosyltransferase 2-like |
| TRINITY_DN17538_c0_g1_i1.p1 | -1.3 | 4.3E-05 | -1.2 | 1.5E-03 | anthocyanidin 3-*O*-glucosyltransferase 2-like |
| TRINITY_DN18673_c0_g6_i1.p1 | -2.6 | 1.1E-20 | -2.0 | 2.3E-11 | aquaporin PIP1-3 |
| TRINITY_DN10804_c0_g1_i1.p1 | -2.5 | 9.1E-25 | -1.4 | 1.4E-04 | aquaporin TIP2-1 |
| TRINITY_DN14766_c0_g1_i1.p1 | -2.7 | 4.6E-05 | -3.1 | 5.8E-06 | AT1G76250-like protein |
| TRINITY_DN18158_c0_g2_i1.p1 | -3.5 | 2.3E-34 | -3.7 | 6.1E-32 | auxin-responsive protein IAA13-like |
| TRINITY_DN15760_c4_g1_i1.p1 | -1.0 | 1.9E-05 | -1.9 | 4.1E-11 | auxin-responsive protein IAA13-like |
| TRINITY_DN19561_c1_g1_i4.p1 | -2.2 | 1.6E-24 | -1.3 | 1.7E-04 | auxin-responsive protein IAA14-like |
| TRINITY_DN20699_c0_g2_i1.p3 | -3.9 | 2.0E-26 | -2.7 | 1.2E-03 | BAS94545.1 Os05g0477666 |
| TRINITY_DN21904_c2_g1_i1.p1 | -2.0 | 2.8E-05 | -2.0 | 4.3E-04 | basic blue protein-like |
| TRINITY_DN7357_c0_g1_i1.p1 | -4.5 | 1.2E-39 | -2.4 | 8.8E-05 | b-box domain protein 30 |
| TRINITY_DN17526_c1_g1_i12.p1 | -1.1 | 7.0E-10 | -1.0 | 1.6E-06 | BEL1-like homeodomain protein 1 |
| TRINITY_DN11541_c0_g1_i2.p1 | -1.3 | 4.6E-04 | -1.4 | 4.9E-05 | benzyl alcohol *O*-benzoyltransferase-like |
| TRINITY_DN10421_c0_g1_i1.p1 | -2.4 | 8.4E-19 | -2.4 | 2.5E-17 | berberine bridge enzyme-like 13 |
| TRINITY_DN13114_c0_g1_i1.p1 | -2.4 | 1.7E-05 | -2.8 | 4.4E-08 | berberine bridge enzyme-like 13 |
| TRINITY_DN21484_c3_g5_i1.p1 | -1.1 | 4.3E-03 | -1.5 | 9.1E-05 | berberine bridge enzyme-like 21 |
| TRINITY_DN16962_c1_g1_i1.p1 | -1.2 | 9.3E-03 | -1.8 | 9.8E-05 | berberine bridge enzyme-like 21 |
| TRINITY_DN15000_c1_g1_i2.p1 | -1.6 | 2.4E-10 | -1.6 | 1.8E-10 | *β*-glucosidase 12-like |
| TRINITY_DN17307_c0_g5_i1.p1 | -5.1 | 9.5E-03 | -5.8 | 3.9E-08 | *β*-glucosidase 12-like |
| TRINITY_DN20361_c2_g1_i7.p1 | -1.4 | 1.2E-06 | -1.4 | 2.1E-05 | *β*-glucosidase 12-like |
| TRINITY_DN20361_c2_g4_i1.p1 | -1.2 | 8.5E-03 | -1.4 | 9.0E-04 | *β*-glucosidase 12-like |
| TRINITY_DN16967_c0_g2_i2.p1 | -1.8 | 1.4E-13 | -1.5 | 5.2E-07 | bifunctional epoxide hydrolase 2-like |
| TRINITY_DN16934_c0_g2_i7.p1 | -1.6 | 1.7E-06 | -1.5 | 8.7E-03 | BTB/POZ domain-containing protein At1g03010-like isoform X1 |
| TRINITY_DN3781_c0_g1_i1.p1 | -3.6 | 1.3E-09 | -2.9 | 1.1E-05 | butyrate-CoA ligase AAE11, peroxisomal-like |
| TRINITY_DN12410_c0_g1_i1.p1 | -2.3 | 8.6E-08 | -1.3 | 2.3E-03 | calcium-binding protein PBP1-like |
| TRINITY_DN20979_c0_g5_i1.p1 | -3.7 | 5.2E-03 | -4.5 | 5.7E-04 | calcium-transporting ATPase 4, endoplasmic reticulum-type-like |
| TRINITY_DN11012_c0_g1_i2.p1 | -1.1 | 3.4E-03 | -2.0 | 1.5E-03 | carboxylesterase 1-like |
| TRINITY_DN18837_c2_g1_i6.p1 | -2.6 | 6.7E-27 | -2.6 | 8.2E-23 | cellulose synthase A catalytic subunit 4 [UDP-forming] |
| TRINITY_DN17207_c0_g1_i2.p1 | -2.9 | 1.1E-22 | -2.7 | 4.3E-18 | cellulose synthase A catalytic subunit 7 [UDP-forming] |
| TRINITY_DN17207_c0_g3_i3.p1 | -1.8 | 5.3E-15 | -1.7 | 4.5E-11 | cellulose synthase A catalytic subunit 8 [UDP-forming] |
| TRINITY_DN16863_c1_g2_i3.p1 | -1.3 | 4.5E-04 | -1.8 | 3.5E-06 | cinnamoyl-CoA reductase 1-like |
| TRINITY_DN19465_c0_g3_i1.p1 | -2.0 | 3.5E-10 | -2.3 | 3.3E-12 | class I SAM-dependent methyltransferase |
| TRINITY_DN17126_c0_g1_i1.p1 | -2.3 | 2.1E-13 | -2.1 | 7.2E-09 | COBRA-like protein 4 |
| TRINITY_DN8670_c0_g1_i1.p1 | -3.1 | 8.1E-50 | -2.5 | 5.0E-27 | cysteine protease XCP1-like |
| TRINITY_DN17220_c0_g1_i10.p1 | -3.5 | 4.8E-24 | -1.5 | 1.3E-03 | cysteine-rich receptor-like protein kinase 10 |
| TRINITY_DN3828_c0_g1_i1.p1 | -2.9 | 1.9E-14 | -2.4 | 2.4E-10 | cytochrome b561 and DOMON domain-containing protein At2g04850 |
| TRINITY_DN18670_c2_g2_i2.p1 | -3.6 | 3.6E-06 | -3.2 | 2.3E-03 | cytochrome b561 and DOMON domain-containing protein At3g61750 |
| TRINITY_DN18809_c1_g2_i1.p1 | -2.5 | 6.2E-13 | -1.7 | 1.0E-03 | cytochrome b561 domain-containing protein At4g18260-like |
| TRINITY_DN20980_c2_g1_i2.p1 | -2.7 | 2.2E-18 | -1.6 | 5.5E-09 | cytochrome P450 71A1-like |
| TRINITY_DN16953_c0_g4_i1.p1 | -8.2 | 1.8E-08 | -9.2 | 7.3E-25 | dCTP pyrophosphatase 1-like |
| TRINITY_DN12480_c0_g1_i1.p1 | -4.6 | 3.9E-53 | -2.1 | 6.5E-08 | dirigent protein 24-like |
| TRINITY_DN8819_c0_g1_i1.p1 | -8.0 | 1.8E-61 | -4.3 | 2.6E-03 | dirigent protein 25-like |
| TRINITY_DN20765_c3_g6_i1.p1 | -3.8 | 7.0E-20 | -2.1 | 2.9E-04 | disease resistance RPP13-like protein 4 |
| TRINITY_DN20765_c1_g1_i1.p1 | -3.2 | 1.2E-06 | -2.4 | 2.6E-03 | disease resistance RPP13-like protein 4 |
| TRINITY_DN17856_c0_g1_i2.p1 | -1.1 | 7.9E-07 | -1.1 | 1.0E-05 | DNA damage-binding protein 2 |
| TRINITY_DN15723_c0_g5_i1.p1 | -4.3 | 6.5E-40 | -3.2 | 1.6E-15 | DNA damage-repair/toleration protein DRT100-like |
| TRINITY_DN19514_c2_g4_i1.p1 | -1.3 | 1.6E-03 | -1.3 | 2.7E-03 | dof zinc finger protein DOF3.4-like |
| TRINITY_DN17998_c2_g1_i1.p1 | -2.7 | 3.0E-20 | -1.5 | 6.1E-03 | dolichyl-phosphate beta-glucosyltransferase-like isoform X1 |
| TRINITY_DN18950_c0_g2_i1.p1 | -2.3 | 1.0E-03 | -2.9 | 9.5E-10 | dual specificity protein kinase |
| TRINITY_DN11010_c0_g1_i1.p1 | -1.4 | 2.9E-03 | -1.4 | 8.6E-03 | DUF3511 domain-containing protein |
| TRINITY_DN14711_c0_g1_i1.p1 | -3.9 | 2.8E-18 | -2.3 | 5.8E-03 | DUF4228 domain protein |
| TRINITY_DN12690_c0_g1_i4.p1 | -2.5 | 3.8E-11 | -1.6 | 6.0E-03 | DUF936 domain-containing protein |
| TRINITY_DN12548_c0_g1_i1.p1 | -1.2 | 4.1E-05 | -1.1 | 2.2E-03 | E3 ubiquitin-protein ligase RMA1H1-like |
| TRINITY_DN16669_c1_g2_i4.p1 | -4.5 | 3.2E-17 | -2.6 | 4.9E-03 | ent-kaurenoic acid oxidase 1-like |
| TRINITY_DN13531_c0_g1_i1.p1 | -1.8 | 1.6E-04 | -3.4 | 4.0E-08 | epidermis-specific secreted glycoprotein EP1-like |
| TRINITY_DN15662_c0_g1_i1.p1 | -1.6 | 4.7E-10 | -1.2 | 1.9E-05 | ethylene-responsive transcription factor 5-like |
| TRINITY_DN12725_c0_g1_i1.p1 | -1.2 | 5.5E-04 | -1.3 | 3.5E-04 | ethylene-responsive transcription factor TINY-like |
| TRINITY_DN20699_c0_g3_i1.p1 | -4.0 | 2.4E-29 | -3.0 | 8.1E-05 | expansin-A8-like |
| TRINITY_DN13598_c0_g1_i1.p1 | -5.4 | 4.0E-50 | -5.2 | 1.3E-80 | expansin-like B1 |
| TRINITY_DN22200_c4_g1_i1.p1 | -2.2 | 2.2E-09 | -2.3 | 1.8E-08 | fasciclin-like arabinogalactan protein 11 |
| TRINITY_DN20564_c0_g1_i12.p1 | -4.3 | 1.1E-03 | -4.2 | 4.7E-03 | F-box/kelch-repeat protein At3g23880-like |
| TRINITY_DN19204_c0_g4_i2.p1 | -1.4 | 9.1E-06 | -1.3 | 1.8E-03 | F-box/LRR-repeat protein At3g26922-like |
| TRINITY_DN15844_c0_g1_i1.p1 | -4.1 | 3.4E-14 | -2.6 | 2.4E-03 | flavin-containing monooxygenase FMO GS-OX-like 9 |
| TRINITY_DN19622_c3_g2_i1.p1 | -2.9 | 1.3E-20 | -1.2 | 5.8E-03 | flavonol sulfotransferase-like |
| TRINITY_DN14247_c0_g3_i1.p1 | -2.8 | 1.4E-20 | -1.6 | 6.7E-04 | *γ*-tocopherol methyltransferase |
| TRINITY_DN17672_c4_g2_i2.p1 | -2.2 | 1.7E-13 | -1.4 | 2.7E-03 | GATA transcription factor 12-like |
| TRINITY_DN17624_c3_g1_i2.p1 | -3.3 | 4.3E-03 | -3.8 | 1.4E-04 | gibberellin-regulated protein 1-like |
| TRINITY_DN18029_c3_g2_i2.p1 | -1.9 | 3.2E-12 | -1.1 | 3.8E-03 | glucan endo-1,3-*β*-glucosidase 1 |
| TRINITY_DN18136_c0_g6_i2.p1 | -2.6 | 1.3E-23 | -1.6 | 7.5E-06 | glucomannan 4-*β*-mannosyltransferase 9-like |
| TRINITY_DN16753_c1_g3_i2.p1 | -1.8 | 6.1E-03 | -2.3 | 1.7E-04 | glucuronoxylan 4-*O*-methyltransferase 1 |
| TRINITY_DN15852_c3_g7_i1.p1 | -1.8 | 2.1E-08 | -1.5 | 1.1E-03 | glutamate decarboxylase |
| TRINITY_DN18075_c1_g6_i1.p1 | -1.2 | 7.4E-05 | -1.2 | 1.8E-04 | glutaredoxin |
| TRINITY_DN19142_c2_g1_i3.p1 | -4.2 | 3.0E-17 | -2.7 | 1.5E-07 | glycine-rich protein-like |
| TRINITY_DN13537_c0_g1_i1.p1 | -2.0 | 1.6E-11 | -2.1 | 8.1E-09 | G-type lectin S-receptor-like serine/threonine-protein kinase LECRK1 |
| TRINITY_DN21167_c0_g4_i2.p1 | -1.0 | 7.9E-04 | -1.0 | 6.0E-03 | G-type lectin S-receptor-like serine/threonine-protein kinase LECRK3 |
| TRINITY_DN20053_c1_g2_i3.p1 | -3.5 | 3.3E-43 | -2.0 | 1.5E-11 | heat shock cognate 70 kDa protein 2-like |
| TRINITY_DN16490_c2_g1_i1.p1 | -1.7 | 8.5E-14 | -1.1 | 1.3E-04 | heavy metal-associated isoprenylated plant protein 35-like |
| TRINITY_DN20021_c0_g2_i1.p1 | -1.4 | 1.8E-03 | -2.3 | 3.9E-06 | heavy metal-associated isoprenylated plant protein 47-like |
| TRINITY_DN11446_c0_g1_i2.p1 | -2.6 | 2.4E-25 | -1.8 | 7.0E-06 | homeobox-leucine zipper protein ATHB-52-like |
| TRINITY_DN14860_c0_g2_i4.p1 | -1.9 | 2.3E-14 | -1.1 | 8.5E-04 | homeobox-leucine zipper protein HAT4-like |
| TRINITY_DN16912_c0_g1_i1.p1 | -1.9 | 1.3E-10 | -1.2 | 4.6E-03 | inactive protein RESTRICTED TEV MOVEMENT 2-like |
| TRINITY_DN17541_c1_g2_i1.p1 | -1.5 | 1.3E-05 | -2.0 | 2.9E-11 | isoflavone reductase homolog |
| TRINITY_DN18180_c0_g1_i3.p1 | -1.4 | 9.1E-05 | -1.7 | 5.0E-07 | isoflavone reductase-like protein |
| TRINITY_DN13217_c0_g1_i7.p1 | -1.7 | 7.6E-12 | -1.1 | 2.0E-03 | IST1-like protein |
| TRINITY_DN18176_c1_g5_i1.p2 | -2.7 | 4.8E-18 | -1.1 | 6.4E-04 | kirola-like |
| TRINITY_DN18857_c1_g3_i2.p1 | -2.8 | 1.6E-12 | -2.9 | 9.4E-10 | laccase-11-like |
| TRINITY_DN12716_c1_g1_i1.p1 | -1.7 | 3.2E-11 | -1.8 | 1.3E-09 | laccase-11-like |
| TRINITY_DN13892_c0_g1_i1.p1 | -2.8 | 2.3E-10 | -2.6 | 5.2E-07 | laccase-4-like |
| TRINITY_DN20142_c3_g3_i3.p1 | -2.7 | 5.0E-09 | -2.6 | 1.4E-06 | laccase-4-like |
| TRINITY_DN19024_c0_g1_i1.p1 | -3.8 | 1.4E-07 | -2.9 | 2.7E-03 | laccase-4-like |
| TRINITY_DN12072_c0_g1_i3.p1 | -2.1 | 4.1E-07 | -1.7 | 2.2E-03 | L-ascorbate peroxidase 3 |
| TRINITY_DN12385_c0_g1_i1.p1 | -1.9 | 3.1E-14 | -1.0 | 3.8E-03 | leucine-rich repeat extensin-like protein 3 |
| TRINITY_DN13921_c0_g1_i1.p1 | -2.6 | 2.6E-06 | -2.4 | 1.4E-06 | L-gulonolactone oxidase 3 |
| TRINITY_DN21468_c0_g1_i12.p1 | -5.5 | 9.0E-03 | -6.4 | 4.1E-03 | low affinity sulfate transporter 3-like |
| TRINITY_DN17112_c3_g4_i10.p1 | -3.3 | 3.1E-25 | -2.0 | 1.4E-04 | LRR protein |
| TRINITY_DN14424_c0_g1_i1.p1 | -3.3 | 6.8E-31 | -2.0 | 5.3E-08 | LRR receptor-like serine/threonine-protein kinase GSO1 |
| TRINITY_DN16500_c0_g1_i1.p1 | -5.3 | 1.5E-16 | -5.0 | 4.8E-27 | lysine histidine transporter-like 8 |
| TRINITY_DN15752_c0_g1_i3.p1 | -3.2 | 7.0E-11 | -3.0 | 3.9E-08 | lysM domain receptor-like kinase 3-like |
| TRINITY_DN15961_c0_g8_i4.p1 | -4.0 | 6.6E-49 | -2.4 | 1.2E-21 | mannan endo-1,4-beta-mannosidase 7 |
| TRINITY_DN13426_c0_g1_i1.p1 | -2.9 | 1.9E-20 | -2.4 | 2.0E-11 | mavicyanin |
| TRINITY_DN14700_c0_g1_i2.p1 | -3.1 | 1.6E-13 | -2.2 | 8.0E-04 | MDIS1-interacting receptor like kinase 1 |
| TRINITY_DN20551_c3_g2_i7.p1 | -6.6 | 9.0E-09 | -6.8 | 1.8E-09 | MDIS1-interacting receptor like kinase 2-like |
| TRINITY_DN12870_c0_g1_i1.p1 | -1.3 | 7.1E-07 | -1.4 | 3.6E-06 | MDIS1-interacting receptor like kinase 2-like |
| TRINITY_DN17919_c0_g1_i10.p1 | -4.9 | 3.7E-12 | -3.9 | 8.1E-06 | MDIS1-interacting receptor like kinase 2-like |
| TRINITY_DN20551_c3_g2_i3.p1 | -3.0 | 4.3E-06 | -2.6 | 1.6E-03 | MDIS1-interacting receptor like kinase 2-like |
| TRINITY_DN19216_c0_g1_i9.p1 | -3.0 | 1.1E-08 | -2.0 | 5.6E-03 | MDIS1-interacting receptor like kinase 2-like |
| TRINITY_DN12194_c0_g1_i2.p1 | -1.9 | 7.6E-05 | -1.4 | 7.1E-03 | membrane protein PM19L |
| TRINITY_DN20538_c0_g2_i2.p1 | -1.1 | 8.3E-06 | -2.3 | 4.4E-22 | metal transporter nramp5-like |
| TRINITY_DN10369_c0_g1_i1.p1 | -1.5 | 2.0E-04 | -1.7 | 1.0E-04 | metalloendoproteinase 2-MMP |
| TRINITY_DN10198_c0_g1_i1.p1 | -1.9 | 6.5E-09 | -1.7 | 3.5E-07 | methionine *γ*-lyase |
| TRINITY_DN15548_c0_g2_i5.p1 | -2.3 | 1.6E-11 | -1.7 | 6.6E-05 | microtubule-associated protein 70-5 |
| TRINITY_DN16444_c3_g1_i10.p1 | -2.3 | 1.9E-17 | -1.4 | 5.2E-04 | MLO-like protein 3 |
| TRINITY_DN19841_c1_g4_i2.p1 | -2.6 | 8.0E-19 | -2.5 | 1.7E-15 | ---NA***--- |
| TRINITY_DN16034_c0_g10_i1.p1 | -1.8 | 3.1E-10 | -1.7 | 5.9E-12 | ---NA--- |
| TRINITY_DN20227_c2_g4_i1.p1 | -2.1 | 1.7E-10 | -2.3 | 1.2E-11 | ---NA--- |
| TRINITY_DN12547_c0_g1_i1.p1 | -3.9 | 2.3E-46 | -1.7 | 1.1E-07 | ---NA--- |
| TRINITY_DN12547_c0_g1_i2.p1 | -4.3 | 1.3E-55 | -1.9 | 6.7E-07 | ---NA--- |
| TRINITY_DN19539_c0_g4_i1.p1 | -2.9 | 4.2E-10 | -2.5 | 2.1E-06 | ---NA--- |
| TRINITY_DN14235_c0_g1_i2.p3 | -1.5 | 6.8E-05 | -1.5 | 5.2E-06 | ---NA--- |
| TRINITY_DN19876_c1_g1_i1.p3 | -5.4 | 1.7E-39 | -3.0 | 4.2E-05 | ---NA--- |
| TRINITY_DN16284_c0_g2_i5.p1 | -2.2 | 8.4E-24 | -1.2 | 5.2E-05 | ---NA--- |
| TRINITY_DN8442_c0_g1_i1.p1 | -3.5 | 3.3E-04 | -3.3 | 7.1E-03 | ---NA--- |
| TRINITY_DN10424_c0_g1_i1.p1 | -2.1 | 9.9E-03 | -2.3 | 8.3E-03 | NAC domain-containing protein 73-like |
| TRINITY_DN19843_c1_g4_i1.p1 | -1.4 | 5.2E-07 | -1.3 | 3.4E-04 | NDR1/HIN1-like protein 13 |
| TRINITY_DN17078_c0_g2_i2.p1 | -6.1 | 1.6E-90 | -3.8 | 7.1E-11 | non-specific lipid-transfer protein 1-like |
| TRINITY_DN17699_c2_g3_i10.p1 | -3.1 | 4.9E-06 | -3.0 | 6.1E-15 | *O*-acyltransferase WSD1-like |
| TRINITY_DN21195_c0_g1_i13.p1 | -2.2 | 3.0E-12 | -3.1 | 2.5E-20 | patatin-like protein 2 |
| TRINITY_DN21195_c0_g2_i20.p1 | -2.7 | 7.4E-06 | -2.7 | 4.9E-07 | patatin-like protein 2 |
| TRINITY_DN42377_c0_g1_i1.p1 | -2.0 | 2.3E-03 | -3.9 | 1.9E-18 | pathogenesis-related protein 1 |
| TRINITY_DN14072_c0_g1_i1.p1 | -5.4 | 3.0E-40 | -3.8 | 1.4E-10 | pathogenesis-related protein 1-like |
| TRINITY_DN19638_c1_g1_i6.p1 | -1.8 | 8.0E-15 | -1.3 | 2.0E-07 | peptide-N4-(N-acetyl-beta-glucosaminyl)asparagine amidase A-like |
| TRINITY_DN13697_c1_g3_i2.p1 | -4.1 | 3.6E-08 | -5.0 | 6.2E-14 | peroxidase 3-like |
| TRINITY_DN19173_c0_g1_i3.p1 | -2.0 | 5.2E-11 | -3.1 | 2.4E-25 | peroxidase 64-like |
| TRINITY_DN16705_c0_g3_i3.p1 | -1.3 | 2.1E-04 | -2.1 | 2.2E-09 | peroxidase 64-like |
| TRINITY_DN15229_c1_g6_i1.p1 | -5.4 | 3.3E-68 | -2.4 | 2.4E-04 | peroxidase 72-like |
| TRINITY_DN14448_c0_g1_i1.p1 | -1.3 | 8.8E-08 | -1.1 | 5.3E-04 | peroxidase 72-like |
| TRINITY_DN13997_c0_g1_i3.p1 | -9.3 | 6.1E-23 | -5.8 | 2.5E-03 | phosphate transporter PHO1 homolog 1 |
| TRINITY_DN14346_c1_g1_i3.p1 | -1.7 | 3.1E-22 | -1.0 | 3.9E-06 | phosphoinositide phospholipase C 2-like |
| TRINITY_DN18816_c1_g1_i1.p1 | -1.1 | 4.2E-03 | -1.4 | 3.2E-04 | phospholipase A1-I*γ*3, chloroplastic |
| TRINITY_DN14837_c0_g1_i3.p1 | -5.0 | 4.2E-18 | -2.9 | 2.2E-03 | phospholipase D *α* 4 |
| TRINITY_DN11359_c0_g1_i1.p1 | -2.3 | 3.1E-03 | -3.2 | 1.2E-03 | PLATZ transcription factor family protein |
| TRINITY_DN16920_c1_g7_i1.p1 | -3.9 | 1.1E-18 | -3.0 | 9.5E-07 | pollen Ole e 1 allergen/extensin |
| TRINITY_DN13252_c0_g1_i3.p1 | -4.5 | 1.8E-19 | -3.2 | 7.7E-09 | polygalacturonase 1 *β*-like protein 3 |
| TRINITY_DN13725_c0_g1_i7.p1 | -2.2 | 4.4E-07 | -1.6 | 1.4E-03 | potassium channel AKT1-like |
| TRINITY_DN19111_c0_g2_i2.p1 | -1.8 | 1.0E-03 | -1.8 | 3.0E-03 | predicted protein |
| TRINITY_DN18839_c3_g3_i1.p1 | -1.4 | 3.0E-07 | -1.0 | 3.8E-03 | probable *β*-1,3-galactosyltransferase 2 isoform X1 |
| TRINITY_DN9878_c0_g1_i1.p1 | -1.7 | 4.6E-05 | -2.1 | 5.1E-07 | probable *β*-1,4-xylosyltransferase IRX9 |
| TRINITY_DN11699_c0_g1_i1.p1 | -2.1 | 1.2E-06 | -1.0 | 9.3E-04 | probable CCR4-associated factor 1 homolog 11 |
| TRINITY_DN14486_c0_g1_i3.p1 | -1.3 | 1.9E-06 | -1.1 | 2.4E-05 | probable E3 ubiquitin-protein ligase RHY1A |
| TRINITY_DN12044_c0_g1_i2.p1 | -1.9 | 2.1E-08 | -2.1 | 6.0E-10 | probable galacturonosyltransferase 12 |
| TRINITY_DN16656_c2_g1_i15.p1 | -2.3 | 1.0E-08 | -1.7 | 1.9E-04 | probable glycosyltransferase At3g07620 |
| TRINITY_DN19241_c1_g3_i1.p1 | -2.4 | 1.7E-05 | -2.2 | 4.8E-04 | probable glycosyltransferase At5g03795 |
| TRINITY_DN14363_c0_g1_i5.p1 | -1.2 | 1.3E-03 | -1.1 | 1.3E-03 | probable inactive receptor-like protein kinase At3g56050 |
| TRINITY_DN2231_c0_g1_i1.p1 | -4.2 | 2.9E-04 | -3.9 | 7.1E-03 | probable leucine-rich repeat receptor-like protein kinase At1g68400 |
| TRINITY_DN15987_c2_g6_i1.p1 | -3.1 | 2.1E-25 | -2.2 | 4.6E-12 | probable leucine-rich repeat receptor-like protein kinase At5g49770 |
| TRINITY_DN15095_c0_g1_i1.p1 | -1.8 | 9.9E-13 | -1.3 | 3.6E-08 | probable LRR receptor-like serine/threonine-protein kinase At1g14390 |
| TRINITY_DN21620_c1_g6_i2.p1 | -1.7 | 2.0E-08 | -1.3 | 8.0E-05 | probable mannitol dehydrogenase |
| TRINITY_DN18048_c1_g5_i2.p1 | -1.5 | 5.6E-11 | -1.0 | 9.5E-03 | probable methyltransferase PMT23 |
| TRINITY_DN10753_c0_g1_i2.p1 | -4.6 | 5.3E-21 | -3.1 | 1.3E-09 | probable pectate lyase 5 |
| TRINITY_DN21263_c0_g3_i4.p1 | -3.1 | 2.8E-24 | -2.4 | 1.2E-19 | probable pectate lyase 8 |
| TRINITY_DN16419_c1_g5_i1.p1 | -3.3 | 1.2E-11 | -2.9 | 6.4E-07 | probable pectinesterase 8 |
| TRINITY_DN10850_c0_g1_i1.p1 | -2.9 | 3.8E-08 | -2.6 | 1.7E-06 | probable pectinesterase 8 |
| TRINITY_DN16133_c1_g3_i3.p1 | -1.8 | 8.5E-05 | -2.2 | 7.6E-07 | probable polygalacturonase At1g80170 |
| TRINITY_DN17291_c0_g1_i2.p1 | -1.4 | 7.6E-05 | -2.8 | 3.9E-20 | probable receptor-like serine/threonine-protein kinase At5g57670 |
| TRINITY_DN17920_c1_g1_i13.p1 | -2.1 | 5.6E-09 | -1.5 | 1.9E-05 | probable trehalose-phosphate phosphatase F |
| TRINITY_DN16890_c1_g1_i12.p1 | -4.0 | 1.3E-36 | -2.5 | 3.9E-07 | probable WRKY transcription factor 50 |
| TRINITY_DN12468_c0_g1_i2.p1 | -1.9 | 2.0E-15 | -1.9 | 1.2E-11 | probable xyloglucan endotransglucosylase/hydrolase protein 28 |
| TRINITY_DN17577_c1_g6_i2.p1 | -4.1 | 7.3E-103 | -2.5 | 3.0E-25 | probable xyloglucan endotransglucosylase/hydrolase protein 6 |
| TRINITY_DN21346_c1_g1_i3.p1 | -1.2 | 5.3E-07 | -1.1 | 5.9E-03 | probable xyloglucan endotransglucosylase/hydrolase protein 8 |
| TRINITY_DN15929_c0_g1_i1.p1 | -4.2 | 5.9E-46 | -2.3 | 8.3E-08 | probably inactive leucine-rich repeat receptor-like protein kinase At2g25790 |
| TRINITY_DN10706_c0_g1_i1.p1 | -2.7 | 2.9E-07 | -2.7 | 1.8E-06 | proline-rich receptor-like protein kinase PERK2 |
| TRINITY_DN16469_c3_g1_i4.p1 | -3.0 | 1.1E-06 | -3.1 | 1.0E-12 | protein ASPARTIC PROTEASE IN GUARD CELL 1 |
| TRINITY_DN16469_c3_g1_i1.p1 | -2.5 | 8.3E-06 | -3.0 | 6.8E-10 | protein ASPARTIC PROTEASE IN GUARD CELL 1 |
| TRINITY_DN12924_c0_g1_i1.p1 | -1.2 | 2.0E-05 | -1.6 | 1.1E-04 | protein AUXIN-REGULATED GENE INVOLVED IN ORGAN SIZE-like |
| TRINITY_DN20101_c0_g2_i1.p2 | -5.3 | 1.3E-48 | -2.8 | 3.3E-06 | protein CASPARIAN STRIP INTEGRITY FACTOR 1-like |
| TRINITY_DN10782_c0_g1_i3.p1 | -2.8 | 5.2E-12 | -2.2 | 2.6E-05 | protein DETOXIFICATION 27-like |
| TRINITY_DN17186_c0_g3_i19.p1 | -1.4 | 2.4E-04 | -1.4 | 1.4E-03 | protein DETOXIFICATION 40-like |
| TRINITY_DN20974_c4_g1_i1.p1 | -2.2 | 2.4E-05 | -2.0 | 2.6E-03 | protein DMP2-like |
| TRINITY_DN10336_c0_g1_i1.p1 | -2.1 | 1.9E-04 | -2.2 | 7.7E-04 | protein DMP4-like |
| TRINITY_DN11217_c0_g1_i1.p1 | -2.2 | 1.5E-09 | -1.9 | 1.0E-05 | protein ESKIMO 1-like |
| TRINITY_DN21895_c4_g3_i2.p1 | -2.2 | 4.0E-15 | -1.3 | 8.5E-05 | protein GLUTAMINE DUMPER 2-like |
| TRINITY_DN14686_c0_g2_i1.p1 | -3.4 | 2.2E-09 | -3.5 | 2.4E-09 | protein IQ-DOMAIN 1 |
| TRINITY_DN11817_c0_g1_i4.p1 | -3.7 | 7.4E-27 | -3.2 | 2.8E-09 | protein IQ-DOMAIN 14-like |
| TRINITY_DN15391_c0_g2_i1.p1 | -1.3 | 8.5E-04 | -1.5 | 2.1E-05 | protein IQ-DOMAIN 14-like |
| TRINITY_DN11064_c0_g1_i1.p1 | -1.8 | 3.0E-06 | -2.6 | 2.8E-11 | protein IRX15-LIKE-like |
| TRINITY_DN17403_c0_g3_i1.p1 | -1.9 | 3.7E-07 | -2.2 | 1.0E-06 | protein LAZY 1-like |
| TRINITY_DN14703_c0_g1_i1.p1 | -1.2 | 2.3E-06 | -1.2 | 4.2E-03 | protein LURP-one-related 8-like |
| TRINITY_DN12062_c0_g1_i1.p1 | -1.7 | 1.4E-03 | -1.8 | 4.8E-03 | protein LYK2 |
| TRINITY_DN14027_c0_g1_i3.p1 | -2.9 | 3.8E-19 | -2.1 | 1.3E-06 | protein NRT1/ PTR FAMILY 5.1 |
| TRINITY_DN13027_c0_g1_i1.p1 | -1.6 | 1.4E-08 | -1.0 | 4.1E-03 | protein SMAX1-LIKE 4-like |
| TRINITY_DN19594_c2_g4_i1.p1 | -3.0 | 9.2E-10 | -3.0 | 9.3E-11 | protein trichome birefringence-like 3 |
| TRINITY_DN13131_c0_g1_i1.p1 | -2.1 | 1.3E-07 | -1.8 | 1.3E-04 | proteoglycan 4-like |
| TRINITY_DN15430_c0_g1_i1.p1 | -5.8 | 6.3E-11 | -4.8 | 3.5E-06 | purine permease 3-like |
| TRINITY_DN20118_c0_g2_i7.p1 | -3.3 | 1.8E-10 | -3.0 | 3.1E-06 | putative calcium-transporting ATPase 13, plasma membrane-type |
| TRINITY_DN22087_c2_g1_i8.p1 | -1.5 | 1.4E-09 | -1.1 | 1.9E-03 | putative disease resistance protein At1g50180 |
| TRINITY_DN19183_c0_g8_i1.p1 | -1.7 | 1.7E-03 | -1.5 | 9.5E-04 | putative disease resistance protein RGA3 |
| TRINITY_DN16483_c2_g3_i4.p1 | -6.0 | 2.0E-144 | -5.1 | 5.0E-39 | putative galactose-binding domain-containing protein |
| TRINITY_DN16013_c2_g8_i2.p1 | -3.1 | 7.0E-07 | -4.9 | 1.9E-24 | putative germin-like protein 2-1 |
| TRINITY_DN20479_c1_g1_i5.p1 | -8.3 | 1.2E-24 | -6.6 | 2.5E-11 | putative inactive cadmium/zinc-transporting ATPase HMA3 |
| TRINITY_DN20811_c0_g4_i1.p1 | -4.3 | 3.2E-22 | -2.5 | 8.1E-10 | putative inactive cadmium/zinc-transporting ATPase HMA3 |
| TRINITY_DN20479_c1_g1_i1.p1 | -3.6 | 9.9E-14 | -1.5 | 3.5E-03 | putative inactive cadmium/zinc-transporting ATPase HMA3 |
| TRINITY_DN21285_c1_g1_i2.p1 | -1.5 | 8.5E-07 | -1.2 | 8.8E-05 | putative late blight resistance protein homolog R1A-10 |
| TRINITY_DN16217_c1_g1_i1.p2 | -4.6 | 8.6E-42 | -2.3 | 3.7E-03 | putative lipid-transfer protein DIR1 |
| TRINITY_DN39373_c0_g1_i1.p1 | -2.1 | 3.0E-03 | -2.1 | 5.9E-04 | putative peroxidase superfamily protein |
| TRINITY_DN18564_c0_g4_i2.p1 | -1.1 | 6.9E-04 | -1.6 | 9.0E-07 | putative phospholipid-transporting ATPase 9 |
| TRINITY_DN17443_c1_g8_i2.p1 | -1.9 | 5.6E-08 | -2.0 | 5.4E-08 | putative wall-associated receptor kinase-like 16 |
| TRINITY_DN20843_c0_g2_i4.p1 | -3.0 | 4.4E-08 | -2.6 | 1.2E-04 | receptor-like protein kinase HSL1 |
| TRINITY_DN11679_c0_g1_i1.p1 | -1.9 | 4.3E-10 | -1.5 | 1.4E-04 | remorin-like |
| TRINITY_DN20445_c1_g1_i15.p1 | -3.3 | 1.4E-10 | -2.5 | 6.4E-04 | replication protein A 70 kDa DNA-binding subunit B-like |
| TRINITY_DN18242_c1_g1_i6.p1 | -4.6 | 6.2E-13 | -3.8 | 2.5E-03 | ribonuclease H-like superfamily protein |
| TRINITY_DN10647_c0_g1_i1.p1 | -1.8 | 1.8E-04 | -1.9 | 5.4E-05 | RING-H2 finger protein ATL52-like |
| TRINITY_DN11040_c0_g1_i1.p1 | -2.7 | 3.8E-12 | -3.0 | 7.0E-15 | serine carboxypeptidase-like |
| TRINITY_DN21954_c0_g2_i2.p1 | -8.3 | 6.5E-14 | -6.4 | 4.5E-05 | serine carboxypeptidase-like 31 |
| TRINITY_DN18446_c1_g1_i1.p1 | -2.1 | 4.6E-11 | -1.8 | 9.5E-06 | serine/threonine-protein kinase UCNL |
| TRINITY_DN17789_c0_g2_i1.p1 | -2.1 | 5.8E-03 | -2.8 | 1.0E-10 | serine/threonine-protein kinase WAG1-like |
| TRINITY_DN19686_c1_g2_i6.p1 | -2.0 | 1.2E-03 | -2.3 | 7.7E-04 | sigma factor binding protein 1, chloroplastic-like |
| TRINITY_DN18930_c0_g1_i1.p1 | -3.2 | 6.7E-06 | -2.5 | 9.2E-08 | subtilisin-like protease SBT1.7 |
| TRINITY_DN21798_c0_g2_i11.p1 | -2.6 | 1.1E-03 | -2.8 | 2.6E-03 | subtilisin-like protease SBT1.7 |
| TRINITY_DN21938_c2_g1_i4.p1 | -1.4 | 4.1E-05 | -1.3 | 2.4E-03 | subtilisin-like protease SBT1.9 |
| TRINITY_DN19793_c0_g1_i2.p1 | -2.7 | 9.5E-12 | -2.2 | 6.4E-07 | subtilisin-like protease SBT4.14 |
| TRINITY_DN21943_c0_g1_i1.p1 | -2.1 | 1.0E-09 | -1.3 | 6.1E-03 | sucrose synthase 7-like |
| TRINITY_DN20692_c0_g2_i3.p1 | -1.1 | 5.2E-09 | -2.1 | 1.1E-14 | sugar carrier protein C-like |
| TRINITY_DN9996_c0_g1_i1.p1 | -1.2 | 1.0E-03 | -1.6 | 2.1E-03 | suppressor protein SRP40-like |
| TRINITY_DN17978_c1_g1_i6.p1 | -1.1 | 4.7E-07 | -1.1 | 8.5E-04 | synaptotagmin-5 isoform X1 |
| TRINITY_DN11714_c0_g1_i1.p1 | -4.1 | 6.7E-27 | -2.6 | 3.5E-06 | tetratricopeptide repeat protein 1 |
| TRINITY_DN17609_c1_g1_i1.p1 | -2.1 | 3.3E-11 | -2.3 | 2.6E-12 | thaumatin-like protein |
| TRINITY_DN31798_c0_g1_i1.p1 | -3.4 | 6.9E-08 | -3.6 | 1.6E-08 | thaumatin-like protein |
| TRINITY_DN17527_c1_g2_i1.p1 | -1.9 | 5.1E-17 | -2.6 | 2.3E-17 | thermospermine synthase ACAULIS5 |
| TRINITY_DN20717_c1_g4_i1.p1 | -3.0 | 8.7E-40 | -1.3 | 7.8E-06 | thioredoxin superfamily protein |
| TRINITY_DN18899_c0_g2_i2.p1 | -4.0 | 2.7E-20 | -3.0 | 4.4E-08 | transcription factor BEE 3-like |
| TRINITY_DN20719_c0_g1_i11.p1 | -1.8 | 2.7E-04 | -3.2 | 2.9E-24 | transcription factor bHLH30-like |
| TRINITY_DN19021_c0_g1_i3.p1 | -1.2 | 7.3E-03 | -3.3 | 2.0E-18 | transcription factor bHLH30-like |
| TRINITY_DN16263_c0_g3_i8.p1 | -1.5 | 5.3E-07 | -1.3 | 8.7E-04 | transcription factor bHLH68-like |
| TRINITY_DN13773_c0_g1_i5.p1 | -3.8 | 5.0E-13 | -2.7 | 3.5E-04 | transcription factor bHLH93-like |
| TRINITY_DN20291_c0_g3_i1.p1 | -1.2 | 3.7E-05 | -1.2 | 6.7E-04 | transcription factor DIVARICATA-like |
| TRINITY_DN19719_c3_g5_i1.p1 | -2.6 | 4.5E-29 | -1.2 | 9.3E-04 | transcription factor MYB1R1 |
| TRINITY_DN18680_c0_g5_i3.p1 | -1.3 | 1.4E-08 | -1.2 | 5.1E-04 | transcription factor MYB61-like |
| TRINITY_DN14235_c0_g1_i1.p2 | -1.9 | 5.4E-12 | -1.8 | 1.5E-13 | transmembrane protein |
| TRINITY_DN11810_c0_g1_i1.p1 | -1.9 | 5.9E-17 | -1.4 | 3.9E-05 | transmembrane protein |
| TRINITY_DN13062_c0_g1_i1.p1 | -2.3 | 4.5E-06 | -1.8 | 6.9E-03 | transmembrane protein |
| TRINITY_DN14830_c0_g1_i1.p1 | -2.6 | 1.7E-06 | -2.0 | 7.6E-03 | transmembrane protein |
| TRINITY_DN17456_c4_g1_i1.p1 | -6.4 | 1.8E-06 | -6.2 | 4.7E-05 | truncated transcription factor CAULIFLOWER A-like |
| TRINITY_DN14891_c1_g1_i1.p1 | -2.7 | 4.2E-03 | -2.2 | 8.4E-03 | uncharacterised protein |
| TRINITY_DN13396_c0_g1_i1.p1 | -2.1 | 3.2E-06 | -2.3 | 8.0E-07 | uncharacterized membrane protein At1g06890-like |
| TRINITY_DN17327_c0_g1_i1.p1 | -1.7 | 2.7E-04 | -1.9 | 9.6E-06 | uncharacterized *N*-acetyltransferase p20-like |
| TRINITY_DN5744_c0_g1_i1.p1 | -7.6 | 7.5E-21 | -4.5 | 2.9E-03 | vacuolar iron transporter homolog 4-like |
| TRINITY_DN20351_c0_g4_i3.p1 | -4.5 | 8.1E-59 | -1.9 | 4.2E-05 | vinorine synthase-like |
| TRINITY_DN21879_c0_g3_i3.p1 | -1.2 | 3.8E-03 | -1.6 | 3.5E-04 | wall-associated receptor kinase-like 1 |
| TRINITY_DN21879_c0_g3_i2.p1 | -2.1 | 1.7E-03 | -2.3 | 1.5E-03 | wall-associated receptor kinase-like 1 |
| TRINITY_DN19232_c0_g1_i2.p1 | -7.1 | 5.9E-46 | -4.4 | 1.7E-06 | WAT1-related protein At1g43650 |
| TRINITY_DN18734_c0_g1_i5.p1 | -4.6 | 5.9E-10 | -2.9 | 6.6E-03 | WAT1-related protein At2g37460-like |
| TRINITY_DN20897_c3_g1_i2.p1 | -8.5 | 3.9E-12 | -6.7 | 3.3E-06 | WAT1-related protein At5g07050-like |
| TRINITY_DN13270_c0_g1_i2.p1 | -2.6 | 8.8E-07 | -2.1 | 9.4E-04 | WEB family protein At1g75720-like |
| TRINITY_DN13176_c0_g1_i1.p1 | -1.4 | 4.3E-05 | -1.4 | 5.4E-03 | zinc finger protein GIS3-like |
| TRINITY_DN18217_c0_g5_i1.p1 | -3.5 | 1.8E-12 | -1.8 | 1.2E-05 | zinc finger protein ZAT10-like |

*FC: fold change, **FDR: false discovery rate, ***NA: not available

| **Table S4** Genes upregulated in basal region of internodal segments. | | | | | |
| --- | --- | --- | --- | --- | --- |
|  | 1b/0b | | 1b/1a | |  |
|  | logFC* | FDR** | logFC* | FDR** | description |
| TRINITY_DN16704_c0_g2_i3.p1 | 2.4 | 9.4E-18 | 1.3 | 5.6E-05 | (+)-neomenthol dehydrogenase-like |
| TRINITY_DN19246_c2_g1_i1.p1 | 10.6 | 4.0E-47 | 3.3 | 1.9E-05 | (*R*,*S*)-reticuline 7-O-methyltransferase-like |
| TRINITY_DN19246_c2_g3_i2.p1 | 11.9 | 3.0E-15 | 5.1 | 6.4E-04 | *(R*,*S*)-reticuline 7-O-methyltransferase-like |
| TRINITY_DN11465_c0_g1_i1.p1 | 3.3 | 1.3E-18 | 1.6 | 3.2E-05 | [Protein-PII] uridylyltransferase |
| TRINITY_DN20403_c0_g1_i4.p1 | 5.6 | 2.0E-58 | 3.0 | 1.1E-13 | 1,4-Dihydroxy-2-naphthoyl-CoA synthase |
| TRINITY_DN9698_c0_g1_i1.p1 | 5.0 | 1.0E-16 | 3.6 | 1.1E-09 | 1,4-dihydroxy-2-naphthoyl-CoA thioesterase 1-like |
| TRINITY_DN11827_c0_g1_i7.p1 | 2.7 | 2.0E-09 | 2.3 | 3.5E-15 | 1-aminocyclopropane-1-carboxylate oxidase |
| TRINITY_DN22122_c1_g1_i3.p1 | 5.0 | 2.2E-39 | 1.9 | 4.2E-05 | 1-aminocyclopropane-1-carboxylate oxidase 1 |
| TRINITY_DN16674_c0_g1_i1.p1 | 9.0 | 1.5E-23 | 1.9 | 2.1E-05 | 1-aminocyclopropane-1-carboxylate oxidase 5-like |
| TRINITY_DN15436_c0_g2_i1.p1 | 3.7 | 1.4E-08 | 2.8 | 2.8E-06 | 1-aminocyclopropane-1-carboxylate oxidase homolog 1-like |
| TRINITY_DN11942_c0_g1_i2.p1 | 5.8 | 5.6E-13 | 3.1 | 2.0E-05 | 1-aminocyclopropane-1-carboxylate synthase |
| TRINITY_DN18976_c3_g2_i1.p2 | 4.6 | 2.5E-38 | 2.9 | 8.3E-08 | 2-alkenal reductase (NADP(+)-dependent)-like |
| TRINITY_DN16583_c2_g2_i1.p1 | 4.8 | 2.8E-38 | 2.9 | 1.5E-07 | 2-alkenal reductase (NADP(+)-dependent)-like |
| TRINITY_DN17363_c4_g2_i1.p1 | 4.7 | 4.6E-38 | 2.8 | 3.9E-07 | 2-alkenal reductase (NADP(+)-dependent)-like |
| TRINITY_DN19564_c1_g1_i3.p1 | 1.8 | 2.1E-14 | 1.9 | 4.1E-16 | 2-hydroxyisoflavanone dehydratase-like |
| TRINITY_DN19177_c0_g2_i1.p1 | 2.0 | 3.1E-07 | 1.8 | 4.6E-05 | 2-hydroxyisoflavanone dehydratase-like |
| TRINITY_DN11154_c0_g1_i1.p1 | 3.2 | 1.3E-13 | 1.5 | 2.0E-04 | 2-methylene-furan-3-one reductase-like |
| TRINITY_DN19527_c1_g2_i1.p1 | 4.2 | 2.0E-11 | 4.9 | 1.5E-09 | 2-oxoglutarate-dependent dioxygenase DAO-like |
| TRINITY_DN16738_c3_g5_i1.p1 | 6.3 | 2.2E-27 | 4.4 | 9.6E-05 | 2-succinylbenzoate-CoA ligase, chloroplastic/peroxisomal |
| TRINITY_DN12569_c0_g1_i1.p1 | 5.3 | 3.2E-31 | 3.5 | 1.7E-07 | 3,9-dihydroxypterocarpan 6A-monooxygenase |
| TRINITY_DN12193_c0_g1_i1.p1 | 1.9 | 4.7E-11 | 1.5 | 3.0E-06 | 3-phosphoserine/phosphohydroxythreonine transaminase |
| TRINITY_DN16587_c1_g4_i2.p1 | 1.4 | 1.5E-12 | 1.0 | 1.0E-03 | 4-hydroxy-3-methylbut-2-en-1-yl diphosphate synthase (ferredoxin), chloroplastic |
| TRINITY_DN19912_c0_g3_i2.p1 | 13.1 | 4.9E-56 | 4.2 | 6.9E-04 | 4-hydroxybenzoate polyprenyltransferase, mitochondrial |
| TRINITY_DN21792_c0_g1_i10.p3 | 4.8 | 1.8E-04 | 4.2 | 5.0E-04 | 60S ribosomal protein L9 |
| TRINITY_DN18166_c0_g2_i2.p1 | 4.3 | 3.9E-05 | 6.1 | 9.3E-07 | 7-deoxyloganetin glucosyltransferase-like |
| TRINITY_DN17973_c2_g3_i1.p1 | 7.4 | 1.4E-31 | 1.9 | 1.6E-05 | 7-deoxyloganetin glucosyltransferase-like |
| TRINITY_DN17973_c2_g6_i1.p1 | 10.0 | 2.7E-31 | 1.5 | 1.5E-03 | 7-deoxyloganetin glucosyltransferase-like |
| TRINITY_DN19402_c2_g2_i4.p1 | 1.5 | 2.9E-08 | 2.0 | 8.7E-14 | AAA-ATPase ASD, mitochondrial-like |
| TRINITY_DN19787_c0_g5_i1.p1 | 1.5 | 4.2E-09 | 1.1 | 1.6E-04 | AAA-ATPase At2g18193-like |
| TRINITY_DN12070_c0_g2_i1.p1 | 2.6 | 1.2E-08 | 1.7 | 3.3E-04 | AAA-ATPase At2g46620-like |
| TRINITY_DN13485_c0_g2_i1.p1 | 2.9 | 2.3E-11 | 1.3 | 8.3E-03 | ABC transporter B family member 9-like |
| TRINITY_DN19861_c1_g2_i3.p1 | 4.6 | 5.5E-37 | 2.9 | 1.9E-15 | ABC transporter G family member 35-like |
| TRINITY_DN19777_c0_g9_i1.p1 | 4.7 | 6.7E-11 | 3.1 | 1.3E-05 | acidic endochitinase-like |
| TRINITY_DN11259_c0_g1_i1.p1 | 9.6 | 4.0E-40 | 2.7 | 9.8E-07 | acidic mammalian chitinase-like |
| TRINITY_DN9320_c0_g1_i1.p1 | 2.9 | 7.8E-03 | 4.5 | 7.1E-04 | Actin cross-linking |
| TRINITY_DN13618_c0_g1_i2.p1 | 1.7 | 1.2E-09 | 1.2 | 9.9E-05 | actin-depolymerizing factor |
| TRINITY_DN15938_c2_g1_i1.p1 | 1.6 | 5.6E-04 | 2.3 | 4.9E-07 | acyl-protein thioesterase 2-like |
| TRINITY_DN20274_c1_g2_i1.p1 | 5.2 | 2.2E-26 | 3.1 | 6.9E-22 | adenine/guanine permease AZG2 |
| TRINITY_DN10280_c0_g1_i1.p1 | 5.3 | 1.5E-14 | 1.7 | 3.6E-04 | adenylate-forming reductase 03009-like |
| TRINITY_DN10827_c0_g1_i1.p1 | 1.2 | 9.6E-06 | 1.3 | 8.9E-07 | alcohol dehydrogenase-like 4 |
| TRINITY_DN17364_c3_g6_i1.p1 | 1.8 | 1.8E-08 | 1.1 | 4.4E-03 | aldehyde dehydrogenase family 2 member B7, mitochondrial-like |
| TRINITY_DN19661_c2_g1_i1.p1 | 2.1 | 8.9E-15 | 2.9 | 9.3E-22 | aldose 1-epimerase-like |
| TRINITY_DN11900_c0_g1_i2.p1 | 1.9 | 8.5E-05 | 1.6 | 3.1E-03 | allene oxide cyclase, chloroplastic-like |
| TRINITY_DN12929_c0_g2_i1.p1 | 4.8 | 4.4E-08 | 3.9 | 5.5E-06 | *α* carbonic anhydrase 7-like |
| TRINITY_DN9919_c0_g1_i1.p1 | 7.0 | 1.5E-21 | 2.0 | 4.2E-06 | *α*/*β* fold hydrolase |
| TRINITY_DN16050_c1_g3_i1.p1 | 1.8 | 3.3E-12 | 1.0 | 3.7E-04 | *α*/*β* hydrolase |
| TRINITY_DN17113_c3_g1_i3.p1 | 8.3 | 2.6E-33 | 3.4 | 7.2E-04 | *α*-dioxygenase 1 |
| TRINITY_DN12269_c0_g1_i2.p1 | 2.4 | 6.0E-11 | 1.7 | 2.9E-04 | amino acid permease 6-like |
| TRINITY_DN19371_c1_g6_i2.p1 | 4.0 | 1.8E-12 | 1.8 | 5.1E-03 | amino acid transporter AVT1I-like |
| TRINITY_DN36271_c0_g1_i1.p1 | 3.6 | 1.4E-05 | 3.2 | 1.7E-04 | amino-acid permease BAT1 homolog |
| TRINITY_DN21981_c3_g6_i11.p1 | 1.3 | 1.4E-04 | 1.3 | 1.5E-03 | aminodeoxychorismate synthase, chloroplastic |
| TRINITY_DN13326_c0_g2_i2.p1 | 6.4 | 4.4E-14 | 3.5 | 5.7E-06 | ammonium transporter 2 |
| TRINITY_DN11362_c0_g1_i1.p1 | 6.4 | 2.2E-14 | 2.8 | 3.8E-04 | ammonium transporter 3 member 1-like |
| TRINITY_DN20320_c0_g2_i1.p1 | 7.4 | 1.8E-33 | 2.7 | 2.5E-07 | AMP-binding protein |
| TRINITY_DN15764_c0_g1_i2.p1 | 1.5 | 1.4E-03 | 1.8 | 8.6E-05 | ankyrin repeat-containing protein At5g02620-like isoform X1 |
| TRINITY_DN12709_c0_g1_i1.p1 | 7.4 | 1.0E-33 | 3.2 | 2.6E-17 | ankyrin repeat-containing protein BDA1-like |
| TRINITY_DN19039_c0_g1_i1.p1 | 4.9 | 3.6E-13 | 3.2 | 1.4E-06 | anthocyanidin 3-*O*-glucosyltransferase 2-like |
| TRINITY_DN17538_c2_g1_i1.p1 | 3.3 | 2.3E-04 | 4.9 | 1.6E-05 | anthocyanidin 3-*O*-glucosyltransferase 2-like |
| TRINITY_DN21896_c0_g2_i1.p1 | 2.2 | 1.2E-06 | 1.9 | 6.6E-05 | anthocyanidin 3-*O*-glucosyltransferase 2-like |
| TRINITY_DN10617_c0_g1_i5.p1 | 2.7 | 2.6E-10 | 1.6 | 1.0E-03 | apoptosis-inducing factor 2-like |
| TRINITY_DN18445_c6_g4_i1.p2 | 3.5 | 2.2E-10 | 1.8 | 8.8E-04 | aquaporin PIP2-1-like |
| TRINITY_DN17107_c1_g1_i3.p1 | 1.3 | 1.0E-06 | 1.7 | 5.8E-10 | arogenate dehydratase/prephenate dehydratase 6, chloroplastic-like |
| TRINITY_DN14442_c0_g2_i1.p1 | 2.6 | 8.6E-16 | 1.6 | 3.0E-07 | arogenate dehydrogenase 1, chloroplastic |
| TRINITY_DN12424_c0_g1_i2.p1 | 3.8 | 1.9E-12 | 1.9 | 1.5E-04 | ASC1-like protein |
| TRINITY_DN15972_c1_g1_i6.p1 | 1.7 | 2.3E-11 | 1.1 | 3.3E-06 | aspartyl protease family protein 1-like |
| TRINITY_DN9926_c0_g1_i1.p1 | 2.6 | 3.3E-06 | 1.7 | 4.5E-05 | aspartyl protease family protein 2-like |
| TRINITY_DN15760_c2_g1_i1.p1 | 1.8 | 2.7E-04 | 2.0 | 1.4E-08 | auxin-induced protein 22D-like |
| TRINITY_DN10483_c0_g1_i1.p1 | 3.0 | 1.9E-03 | 4.8 | 3.5E-06 | auxin-induced protein AUX22-like |
| TRINITY_DN16772_c3_g1_i3.p1 | 2.6 | 2.3E-06 | 3.0 | 2.6E-12 | auxin-responsive protein IAA29-like |
| TRINITY_DN17586_c0_g5_i1.p1 | 4.6 | 9.4E-11 | 3.7 | 8.0E-07 | BAG family molecular chaperone regulator 7 |
| TRINITY_DN21228_c2_g8_i1.p1 | 5.0 | 2.6E-39 | 1.3 | 1.6E-06 | basic 7S globulin-like |
| TRINITY_DN35653_c0_g1_i1.p1 | 1.3 | 1.9E-03 | 1.9 | 6.1E-06 | basic 7S globulin-like |
| TRINITY_DN11639_c0_g1_i1.p1 | 6.0 | 2.0E-63 | 3.1 | 1.9E-09 | basic secretory protease |
| TRINITY_DN22537_c0_g1_i1.p1 | 3.6 | 1.4E-03 | 3.5 | 4.0E-03 | basic secretory protease |
| TRINITY_DN20828_c1_g1_i2.p1 | 2.5 | 5.5E-15 | 2.1 | 3.3E-10 | berberine bridge enzyme-like 18 |
| TRINITY_DN18293_c0_g5_i1.p1 | 1.9 | 1.5E-13 | 1.3 | 3.3E-05 | berberine bridge enzyme-like 18 |
| TRINITY_DN16962_c2_g1_i2.p1 | 9.1 | 7.6E-14 | 2.6 | 4.6E-04 | berberine bridge enzyme-like 18 |
| TRINITY_DN21124_c0_g1_i1.p1 | 7.3 | 7.9E-09 | 2.8 | 4.8E-04 | berberine bridge enzyme-like 18 |
| TRINITY_DN18293_c0_g3_i1.p1 | 1.1 | 6.9E-03 | 1.5 | 1.1E-03 | berberine bridge enzyme-like 18 |
| TRINITY_DN17766_c0_g1_i1.p1 | 5.9 | 2.1E-07 | 3.4 | 1.6E-03 | berberine bridge enzyme-like 18 |
| TRINITY_DN17766_c0_g1_i2.p1 | 1.3 | 5.3E-06 | 1.0 | 5.0E-03 | berberine bridge enzyme-like 18 |
| TRINITY_DN15986_c0_g9_i2.p1 | 7.5 | 4.1E-24 | 2.7 | 2.9E-06 | berberine bridge enzyme-like 21 |
| TRINITY_DN20828_c1_g2_i2.p1 | 2.7 | 3.6E-07 | 3.4 | 1.0E-15 | berberine bridge enzyme-like 8 |
| TRINITY_DN17300_c4_g3_i1.p1 | 11.0 | 1.4E-72 | 1.8 | 3.6E-05 | berberine bridge enzyme-like 8 |
| TRINITY_DN15201_c0_g4_i1.p1 | 6.7 | 9.6E-43 | 2.0 | 1.3E-07 | *β*-amylase 3, chloroplastic |
| TRINITY_DN12949_c0_g2_i2.p1 | 2.4 | 2.7E-14 | 2.2 | 3.0E-11 | *β*-amyrin 28-oxidase-like |
| TRINITY_DN12651_c0_g2_i1.p1 | 1.9 | 1.7E-05 | 1.9 | 7.5E-09 | *β*-amyrin 28-oxidase-like |
| TRINITY_DN14564_c0_g2_i3.p1 | 1.5 | 2.4E-03 | 1.4 | 6.1E-06 | *β*-amyrin 28-oxidase-like |
| TRINITY_DN14275_c0_g1_i1.p1 | 3.4 | 1.6E-18 | 1.0 | 1.7E-03 | *β*-amyrin 28-oxidase-like |
| TRINITY_DN20707_c0_g2_i8.p1 | 4.2 | 1.6E-61 | 1.9 | 6.9E-15 | *β*-amyrin synthase |
| TRINITY_DN20798_c0_g1_i2.p1 | 5.0 | 3.3E-14 | 3.3 | 2.8E-09 | *β*-amyrin synthase |
| TRINITY_DN9801_c0_g1_i1.p1 | 3.7 | 5.2E-21 | 1.1 | 4.1E-03 | *β*-D-glucosyl crocetin beta-1,6-glucosyltransferase-like |
| TRINITY_DN21358_c0_g1_i16.p1 | 3.1 | 8.4E-03 | 3.4 | 6.8E-03 | *β*-glucosidase |
| TRINITY_DN20045_c0_g3_i3.p1 | 8.7 | 5.2E-54 | 3.1 | 1.5E-08 | *β*-glucosidase 12-like |
| TRINITY_DN16376_c0_g1_i2.p1 | 2.2 | 6.8E-16 | 1.4 | 2.0E-06 | *β*-glucosidase 12-like |
| TRINITY_DN20361_c2_g2_i4.p1 | 9.2 | 6.1E-34 | 4.7 | 1.8E-05 | *β*-glucosidase 12-like |
| TRINITY_DN21358_c0_g1_i28.p1 | 1.9 | 1.5E-07 | 1.4 | 3.6E-05 | *β*-glucosidase 12-like |
| TRINITY_DN21358_c0_g1_i5.p1 | 1.9 | 4.0E-11 | 1.3 | 1.1E-04 | *β*-glucosidase 12-like |
| TRINITY_DN20361_c2_g1_i1.p1 | 7.9 | 2.1E-04 | 7.8 | 5.1E-04 | *β*-glucosidase 12-like |
| TRINITY_DN19964_c0_g1_i5.p1 | 1.8 | 5.2E-07 | 1.9 | 1.6E-07 | *β*-glucuronosyltransferase GlcAT14B-like |
| TRINITY_DN20080_c1_g3_i2.p1 | 1.7 | 1.8E-10 | 2.0 | 3.8E-12 | bifunctional epoxide hydrolase 2-like |
| TRINITY_DN15469_c1_g1_i5.p1 | 3.6 | 4.7E-31 | 1.6 | 4.1E-08 | bifunctional epoxide hydrolase 2-like |
| TRINITY_DN17406_c0_g3_i1.p1 | 3.4 | 8.0E-29 | 1.4 | 1.1E-04 | bifunctional epoxide hydrolase 2-like |
| TRINITY_DN14600_c0_g1_i1.p1 | 2.2 | 1.6E-18 | 2.0 | 5.5E-15 | bifunctional inhibitor/plant lipid transfer protein/seed storage helical domain-containing protein |
| TRINITY_DN15299_c0_g1_i3.p1 | 1.6 | 4.1E-19 | 1.1 | 1.0E-07 | bifunctional L-3-cyanoalanine synthase/cysteine synthase 1, mitochondrial |
| TRINITY_DN21105_c1_g2_i4.p1 | 1.8 | 9.3E-07 | 1.5 | 6.4E-05 | bifunctional riboflavin biosynthesis protein RIBA 1, chloroplastic-like |
| TRINITY_DN21768_c0_g1_i1.p1 | 7.2 | 8.0E-08 | 3.0 | 1.6E-03 | big root biomass |
| TRINITY_DN16372_c0_g1_i1.p1 | 1.1 | 9.4E-03 | 1.4 | 5.1E-04 | BTB/POZ domain-containing protein At5g41330 |
| TRINITY_DN19997_c0_g2_i6.p1 | 1.9 | 8.8E-12 | 1.2 | 2.8E-03 | caffeic acid 3-*O*-methyltransferase |
| TRINITY_DN16179_c0_g9_i1.p1 | 9.0 | 2.4E-79 | 3.2 | 5.3E-12 | caffeic acid 3-*O*-methyltransferase-like |
| TRINITY_DN16179_c0_g4_i4.p1 | 5.8 | 1.8E-18 | 4.1 | 3.6E-11 | caffeic acid 3-*O*-methyltransferase-like |
| TRINITY_DN11694_c0_g2_i1.p1 | 5.5 | 1.5E-39 | 3.1 | 2.4E-09 | caffeic acid 3-*O*-methyltransferase-like |
| TRINITY_DN16179_c0_g4_i6.p1 | 10.0 | 7.4E-10 | 9.1 | 8.1E-09 | caffeic acid 3-*O*-methyltransferase-like |
| TRINITY_DN16894_c0_g1_i1.p1 | 10.1 | 5.3E-08 | 10.0 | 2.5E-07 | caffeic acid 3-*O*-methyltransferase-like |
| TRINITY_DN16179_c0_g4_i10.p1 | 2.8 | 1.8E-03 | 2.8 | 2.2E-03 | caffeic acid 3-*O*-methyltransferase-like |
| TRINITY_DN15361_c0_g1_i1.p1 | 5.4 | 7.6E-16 | 3.5 | 7.7E-09 | calcium-binding protein CP1 |
| TRINITY_DN11344_c0_g1_i1.p2 | 1.6 | 1.3E-05 | 1.5 | 2.1E-05 | calcium-binding protein KIC |
| TRINITY_DN20862_c1_g3_i1.p1 | 4.1 | 5.4E-12 | 1.7 | 6.9E-04 | calmodulin |
| TRINITY_DN21141_c2_g1_i9.p1 | 2.2 | 5.8E-05 | 1.6 | 3.9E-06 | calmodulin-binding protein 60 B-like |
| TRINITY_DN15008_c0_g1_i5.p1 | 4.1 | 3.3E-10 | 1.8 | 3.6E-03 | calmodulin-binding protein 60 B-like |
| TRINITY_DN17954_c0_g3_i1.p1 | 6.2 | 1.6E-24 | 2.9 | 5.7E-09 | calmodulin-like protein 11 |
| TRINITY_DN11113_c0_g1_i1.p1 | 2.1 | 2.0E-06 | 4.0 | 3.6E-11 | carboxylesterase 1-like |
| TRINITY_DN13068_c0_g1_i1.p1 | 3.8 | 5.9E-26 | 2.2 | 6.4E-09 | carboxylesterase 1-like |
| TRINITY_DN11380_c0_g1_i1.p1 | 2.2 | 2.6E-09 | 1.2 | 2.7E-03 | carboxylesterase 1-like |
| TRINITY_DN21591_c1_g3_i7.p1 | 4.5 | 5.5E-45 | 1.5 | 3.1E-08 | carotenoid 9,10(9',10')-cleavage dioxygenase 1-like |
| TRINITY_DN10971_c0_g1_i1.p1 | 2.7 | 1.7E-05 | 2.1 | 2.1E-03 | CASP-like protein 1E1 |
| TRINITY_DN12008_c0_g1_i3.p1 | 5.5 | 5.5E-13 | 1.7 | 5.7E-03 | CASP-like protein 4D1 |
| TRINITY_DN11961_c0_g1_i1.p1 | 5.5 | 2.6E-34 | 4.6 | 2.4E-30 | cationic peroxidase 1-like |
| TRINITY_DN20901_c1_g5_i1.p1 | 7.9 | 1.2E-08 | 2.7 | 8.5E-03 | CDP-diacylglycerol-glycerol-3-phosphate 3-phosphatidyltransferase |
| TRINITY_DN20882_c0_g2_i2.p1 | 5.0 | 4.2E-11 | 4.7 | 1.2E-09 | cellulose synthase-like protein G2 |
| TRINITY_DN19581_c5_g1_i23.p1 | 2.5 | 9.4E-15 | 1.3 | 1.6E-03 | cellulose synthase-like protein G3 |
| TRINITY_DN19215_c2_g6_i1.p1 | 8.4 | 1.0E-11 | 2.3 | 4.7E-03 | chemoreceptor A |
| TRINITY_DN17064_c0_g2_i1.p1 | 8.4 | 1.7E-95 | 3.3 | 3.3E-10 | chitinase |
| TRINITY_DN17064_c0_g2_i8.p1 | 5.4 | 2.2E-34 | 2.2 | 1.2E-08 | chitinase |
| TRINITY_DN16843_c2_g1_i2.p1 | 9.1 | 6.5E-70 | 3.1 | 2.0E-07 | chitinase |
| TRINITY_DN16843_c2_g4_i2.p1 | 7.6 | 3.8E-33 | 3.6 | 2.2E-06 | chitinase |
| TRINITY_DN16843_c2_g8_i1.p1 | 2.3 | 7.7E-11 | 1.5 | 4.1E-06 | chitinase |
| TRINITY_DN16843_c2_g1_i1.p1 | 7.1 | 2.0E-62 | 1.2 | 1.6E-04 | chitinase |
| TRINITY_DN18898_c1_g9_i1.p1 | 5.9 | 6.8E-14 | 3.3 | 6.8E-09 | chlorophyllase-2, chloroplastic |
| TRINITY_DN7001_c0_g1_i1.p1 | 3.1 | 8.9E-04 | 2.3 | 7.0E-03 | chromodomain-helicase-DNA-binding protein 9-like |
| TRINITY_DN20908_c0_g5_i1.p1 | 3.4 | 3.1E-11 | 1.9 | 4.4E-04 | cinnamoyl-CoA reductase 1-like |
| TRINITY_DN20486_c0_g1_i2.p1 | 5.7 | 1.1E-18 | 1.6 | 7.6E-04 | citrate-binding protein-like |
| TRINITY_DN18900_c2_g4_i2.p1 | 3.9 | 1.7E-10 | 3.0 | 9.8E-08 | COPII coat assembly protein |
| TRINITY_DN18964_c0_g7_i1.p1 | 3.3 | 1.6E-16 | 1.2 | 7.3E-04 | COPII coat assembly protein |
| TRINITY_DN12196_c0_g1_i1.p1 | 1.2 | 6.1E-04 | 1.5 | 7.4E-04 | copper transporter 6-like |
| TRINITY_DN11978_c0_g1_i1.p1 | 9.1 | 2.1E-87 | 3.0 | 3.1E-19 | cucumber peeling cupredoxin-like |
| TRINITY_DN19946_c0_g5_i2.p1 | 6.2 | 3.2E-16 | 3.3 | 2.3E-06 | cucumber peeling cupredoxin-like |
| TRINITY_DN18660_c2_g2_i1.p1 | 1.9 | 2.7E-10 | 1.0 | 1.5E-03 | cucumber peeling cupredoxin-like |
| TRINITY_DN18471_c1_g4_i1.p1 | 2.1 | 2.2E-12 | 1.2 | 6.8E-04 | cupin domain-containing protein |
| TRINITY_DN17062_c0_g1_i2.p1 | 1.0 | 1.3E-05 | 1.5 | 6.1E-07 | cyclic nucleotide-gated ion channel 1 |
| TRINITY_DN19207_c0_g2_i11.p1 | 1.1 | 6.5E-03 | 1.8 | 6.8E-04 | cyclopropane-fatty-acyl-phospholipid synthase |
| TRINITY_DN21821_c3_g1_i17.p1 | 3.9 | 1.2E-07 | 1.9 | 1.8E-03 | cyclopropane-fatty-acyl-phospholipid synthase |
| TRINITY_DN19207_c0_g2_i1.p1 | 4.0 | 1.3E-23 | 1.3 | 8.0E-03 | cyclopropane-fatty-acyl-phospholipid synthase |
| TRINITY_DN11753_c0_g1_i1.p1 | 4.6 | 8.5E-21 | 1.5 | 1.6E-04 | cysteine/histidine-rich C1 domain family protein |
| TRINITY_DN12425_c0_g1_i1.p1 | 2.0 | 1.4E-09 | 1.3 | 2.4E-04 | cysteine/histidine-rich C1 domain family protein |
| TRINITY_DN14640_c0_g2_i5.p1 | 2.1 | 2.4E-18 | 1.1 | 3.0E-04 | cysteine-rich and transmembrane domain-containing protein WIH1-like |
| TRINITY_DN21004_c1_g3_i1.p1 | 2.3 | 1.4E-15 | 1.5 | 2.4E-06 | cysteine-rich receptor-like protein kinase 2 |
| TRINITY_DN21879_c0_g2_i1.p1 | 1.9 | 4.6E-08 | 1.3 | 1.2E-03 | cysteine-rich receptor-like protein kinase 2 |
| TRINITY_DN14848_c0_g1_i3.p1 | 1.8 | 3.9E-10 | 1.8 | 6.4E-08 | cysteine-rich receptor-like protein kinase 42 |
| TRINITY_DN11536_c0_g1_i1.p1 | 6.4 | 2.2E-56 | 1.7 | 1.1E-07 | cysteine-rich repeat secretory protein 55-like |
| TRINITY_DN4840_c0_g1_i1.p1 | 3.0 | 6.3E-09 | 2.3 | 2.8E-06 | cysteine-rich repeat secretory protein 55-like |
| TRINITY_DN7451_c0_g1_i1.p1 | 8.4 | 3.2E-08 | 5.2 | 3.1E-05 | cysteine-rich repeat secretory protein 55-like |
| TRINITY_DN20199_c4_g3_i1.p1 | 2.2 | 2.3E-10 | 1.5 | 4.0E-06 | cytochrome b561 and DOMON domain-containing protein At5g47530-like |
| TRINITY_DN16961_c0_g1_i1.p1 | 6.3 | 9.7E-50 | 3.4 | 1.4E-12 | cytochrome P450 714C2-like |
| TRINITY_DN15805_c1_g3_i2.p1 | 2.6 | 4.5E-23 | 2.4 | 1.5E-11 | cytochrome P450 714C2-like |
| TRINITY_DN16252_c0_g2_i2.p1 | 7.4 | 4.7E-49 | 2.5 | 2.1E-06 | cytochrome P450 71A1-like |
| TRINITY_DN21782_c1_g2_i2.p1 | 9.9 | 6.0E-25 | 3.7 | 4.9E-11 | cytochrome P450 71D10-like |
| TRINITY_DN19628_c0_g1_i2.p1 | 7.9 | 1.8E-49 | 3.7 | 1.4E-10 | cytochrome P450 71D10-like |
| TRINITY_DN18332_c0_g8_i1.p1 | 5.5 | 1.1E-13 | 4.3 | 1.5E-09 | cytochrome P450 71D10-like |
| TRINITY_DN18895_c0_g1_i1.p1 | 10.1 | 2.4E-27 | 3.4 | 8.9E-08 | cytochrome P450 71D10-like |
| TRINITY_DN18257_c1_g2_i2.p1 | 2.0 | 2.5E-06 | 1.5 | 6.0E-05 | cytochrome P450 71D10-like |
| TRINITY_DN21782_c0_g1_i1.p1 | 7.7 | 4.7E-37 | 3.4 | 1.7E-08 | cytochrome P450 71D9-like |
| TRINITY_DN19628_c0_g11_i1.p1 | 7.1 | 7.6E-47 | 1.7 | 3.4E-04 | cytochrome P450 71D9-like |
| TRINITY_DN9721_c0_g1_i3.p1 | 7.2 | 2.4E-29 | 6.3 | 1.4E-27 | cytochrome P450 724B1 |
| TRINITY_DN10690_c0_g1_i1.p1 | 3.7 | 1.1E-18 | 9.1 | 5.1E-33 | cytochrome P450 78A5-like |
| TRINITY_DN18284_c0_g2_i2.p1 | 7.0 | 1.6E-30 | 2.4 | 1.9E-06 | cytochrome P450 81E8-like |
| TRINITY_DN13315_c0_g2_i2.p1 | 10.2 | 3.9E-67 | 4.0 | 1.2E-04 | cytochrome P450 81E8-like |
| TRINITY_DN16200_c0_g1_i3.p1 | 5.1 | 9.5E-35 | 1.8 | 6.8E-07 | cytochrome P450 83B1-like |
| TRINITY_DN35970_c0_g1_i1.p1 | 6.0 | 1.3E-14 | 1.9 | 7.2E-04 | cytochrome P450 87A3-like |
| TRINITY_DN20991_c2_g4_i6.p1 | 2.3 | 2.7E-18 | 1.8 | 8.0E-10 | cytochrome P450 90B1 |
| TRINITY_DN16387_c0_g1_i3.p1 | 6.5 | 1.3E-05 | 6.4 | 4.4E-05 | cytochrome P450 CYP72A219-like |
| TRINITY_DN21778_c1_g2_i8.p1 | 5.5 | 6.1E-21 | 2.1 | 4.5E-05 | cytochrome P450 CYP72A219-like |
| TRINITY_DN21778_c1_g2_i2.p1 | 1.4 | 7.7E-03 | 1.9 | 1.2E-03 | cytochrome P450 CYP72A219-like |
| TRINITY_DN19930_c7_g1_i1.p1 | 4.6 | 1.2E-03 | 4.5 | 3.3E-03 | cytochrome P450 CYP72A219-like |
| TRINITY_DN18370_c0_g1_i10.p1 | 7.6 | 7.2E-25 | 2.7 | 7.6E-04 | cytochrome P450 CYP82D47-like |
| TRINITY_DN19575_c1_g1_i1.p1 | 11.1 | 2.4E-72 | 7.2 | 3.0E-62 | cytokinin dehydrogenase 3-like |
| TRINITY_DN5916_c0_g1_i1.p1 | 7.9 | 4.5E-08 | 3.7 | 2.2E-03 | cytokinin hydroxylase-like |
| TRINITY_DN21486_c0_g2_i1.p1 | 8.6 | 1.0E-12 | 7.1 | 2.2E-08 | desiccation-related protein PCC13-62-like |
| TRINITY_DN12997_c0_g1_i1.p1 | 1.6 | 1.2E-15 | 1.8 | 5.4E-17 | dirigent protein 22-like |
| TRINITY_DN9400_c0_g1_i1.p1 | 8.4 | 3.9E-10 | 4.8 | 1.3E-06 | dirigent protein 22-like |
| TRINITY_DN39755_c0_g1_i1.p1 | 7.4 | 1.3E-07 | 5.5 | 3.9E-06 | dirigent protein 22-like |
| TRINITY_DN16673_c0_g3_i3.p1 | 6.7 | 7.4E-54 | 1.0 | 9.0E-04 | dirigent protein 22-like |
| TRINITY_DN21452_c1_g1_i1.p1 | 1.7 | 3.4E-03 | 2.1 | 4.6E-04 | disease resistance protein RPM1-like |
| TRINITY_DN21166_c0_g6_i1.p1 | 2.1 | 6.9E-10 | 1.5 | 8.3E-12 | disease resistance protein RPP13-like |
| TRINITY_DN21430_c0_g3_i4.p1 | 2.7 | 2.7E-04 | 3.7 | 1.4E-05 | DNA-directed DNA polymerase |
| TRINITY_DN17869_c0_g2_i4.p1 | 4.2 | 7.9E-22 | 1.4 | 5.2E-03 | DUF1262 family protein |
| TRINITY_DN16527_c0_g1_i2.p2 | 8.4 | 2.7E-15 | 3.9 | 2.2E-08 | DUF3741 domain-containing protein/DUF4378 domain-containing protein |
| TRINITY_DN16527_c0_g1_i1.p1 | 8.2 | 5.7E-11 | 2.9 | 5.9E-04 | DUF3741 domain-containing protein/DUF4378 domain-containing protein |
| TRINITY_DN19577_c1_g1_i1.p1 | 1.6 | 7.6E-03 | 2.1 | 2.1E-03 | dynein light chain 1, cytoplasmic-like |
| TRINITY_DN11911_c0_g1_i1.p1 | 5.5 | 1.3E-10 | 2.0 | 2.8E-03 | E3 ubiquitin-protein ligase ATL41-like |
| TRINITY_DN9711_c0_g1_i1.p1 | 1.6 | 3.3E-06 | 1.4 | 1.1E-06 | E3 ubiquitin-protein ligase ATL6-like |
| TRINITY_DN9155_c0_g1_i1.p1 | 6.6 | 5.0E-07 | 2.4 | 8.0E-03 | E3 ubiquitin-protein ligase PUB24-like |
| TRINITY_DN12515_c0_g1_i1.p1 | 3.1 | 5.0E-05 | 2.2 | 6.7E-03 | E3 ubiquitin-protein ligase RGLG2-like |
| TRINITY_DN19398_c0_g2_i1.p1 | 2.7 | 4.5E-16 | 2.0 | 1.7E-09 | early nodulin-like protein 2 |
| TRINITY_DN14254_c0_g1_i1.p1 | 10.8 | 5.4E-24 | 3.4 | 7.0E-05 | EG45-like domain containing protein |
| TRINITY_DN18328_c1_g4_i2.p1 | 12.7 | 1.7E-27 | 3.4 | 1.7E-04 | EG45-like domain containing protein |
| TRINITY_DN18328_c1_g4_i1.p1 | 7.6 | 8.1E-18 | 3.6 | 8.7E-03 | EG45-like domain containing protein |
| TRINITY_DN16566_c1_g2_i5.p1 | 2.5 | 2.8E-06 | 2.7 | 2.5E-04 | elicitor-responsive protein 1-like |
| TRINITY_DN22317_c0_g1_i1.p1 | 7.4 | 4.5E-10 | 2.6 | 1.7E-03 | elongation of fatty acids protein 3-like |
| TRINITY_DN11725_c0_g1_i1.p1 | 8.2 | 7.5E-68 | 2.7 | 1.2E-08 | endochitinase EP3-like |
| TRINITY_DN11684_c0_g1_i2.p1 | 7.8 | 3.4E-85 | 1.8 | 1.6E-14 | endoglucanase 25-like |
| TRINITY_DN15195_c0_g1_i5.p1 | 3.1 | 1.8E-06 | 2.2 | 4.2E-06 | esterase AGAP003155-like |
| TRINITY_DN14827_c0_g1_i1.p1 | 3.8 | 3.4E-07 | 4.4 | 2.8E-11 | ethylene-responsive transcription factor 2-like |
| TRINITY_DN14952_c0_g1_i1.p1 | 1.7 | 1.5E-04 | 1.9 | 5.5E-09 | exocyst complex component EXO70E2 |
| TRINITY_DN10633_c0_g1_i1.p1 | 4.8 | 1.4E-46 | 2.5 | 9.0E-16 | exocyst complex component EXO70H1-like |
| TRINITY_DN9302_c0_g1_i1.p1 | 2.6 | 3.1E-05 | 2.2 | 2.1E-03 | exocyst complex component EXO70H1-like |
| TRINITY_DN17060_c0_g1_i1.p1 | 3.2 | 1.8E-13 | 2.7 | 3.5E-12 | expansin-A1 |
| TRINITY_DN12765_c0_g1_i1.p1 | 7.0 | 8.0E-09 | 7.0 | 3.0E-08 | expansin-like B1 |
| TRINITY_DN19906_c1_g3_i3.p1 | 1.7 | 1.4E-14 | 2.8 | 2.4E-32 | extensin-2-like |
| TRINITY_DN19906_c1_g3_i4.p1 | 1.4 | 3.2E-13 | 2.3 | 5.4E-25 | extensin-2-like |
| TRINITY_DN19906_c1_g3_i2.p1 | 1.3 | 3.9E-09 | 2.3 | 6.4E-24 | extensin-2-like |
| TRINITY_DN19906_c1_g3_i5.p1 | 1.4 | 7.3E-08 | 2.4 | 1.1E-23 | extensin-2-like |
| TRINITY_DN17912_c0_g1_i1.p1 | 2.6 | 8.4E-18 | 1.5 | 1.7E-04 | FAD-dependent oxidoreductase |
| TRINITY_DN21176_c0_g3_i1.p1 | 2.4 | 1.9E-07 | 1.6 | 2.5E-04 | F-box protein At1g22220-like |
| TRINITY_DN12756_c0_g1_i1.p1 | 4.5 | 5.3E-23 | 3.3 | 1.8E-12 | F-box protein At3g07870-like |
| TRINITY_DN11509_c0_g1_i1.p1 | 3.3 | 3.4E-04 | 3.4 | 1.8E-04 | F-box protein At3g07870-like |
| TRINITY_DN18131_c2_g2_i7.p1 | 5.5 | 2.4E-19 | 2.4 | 1.8E-06 | F-box protein PP2-B15-like |
| TRINITY_DN9401_c0_g1_i1.p1 | 3.0 | 5.1E-07 | 1.6 | 8.9E-03 | F-box/kelch-repeat protein At1g23390 |
| TRINITY_DN17759_c0_g2_i1.p1 | 4.4 | 1.7E-05 | 3.3 | 9.5E-04 | F-box/LRR-repeat protein At3g26922-like |
| TRINITY_DN16124_c3_g2_i1.p1 | 2.3 | 2.9E-23 | 1.3 | 2.0E-07 | ferrochelatase-2, chloroplastic |
| TRINITY_DN15868_c0_g1_i2.p1 | 7.3 | 1.1E-17 | 2.4 | 2.6E-05 | feruloyl CoA ortho-hydroxylase 1-like |
| TRINITY_DN17122_c1_g3_i1.p1 | 2.8 | 6.1E-16 | 1.5 | 5.0E-06 | flavin-containing monooxygenase FMO GS-OX-like 4 |
| TRINITY_DN18230_c0_g1_i1.p1 | 1.4 | 1.0E-04 | 1.4 | 1.0E-04 | formin-like protein 4 |
| TRINITY_DN12687_c0_g1_i4.p1 | 2.9 | 4.8E-19 | 1.2 | 3.7E-04 | GABA transporter 1-like |
| TRINITY_DN15860_c0_g1_i1.p1 | 2.4 | 8.2E-06 | 2.0 | 5.3E-06 | galactose-binding domain-like protein |
| TRINITY_DN20276_c0_g1_i1.p1 | 7.9 | 3.3E-07 | 4.0 | 4.3E-04 | galactoside 2-*α*-L-fucosyltransferase-like |
| TRINITY_DN10360_c0_g1_i1.p1 | 8.8 | 4.0E-18 | 2.3 | 7.4E-05 | gallate 1-*β*-glucosyltransferase-like |
| TRINITY_DN11668_c0_g1_i1.p1 | 6.9 | 2.1E-13 | 2.1 | 8.8E-03 | gallate 1-*β*-glucosyltransferase-like |
| TRINITY_DN17356_c0_g2_i5.p1 | 1.3 | 3.7E-05 | 1.6 | 1.2E-08 | GDSL esterase/lipase 1-like |
| TRINITY_DN17356_c0_g2_i1.p1 | 1.5 | 1.5E-10 | 1.5 | 1.9E-05 | GDSL esterase/lipase 1-like |
| TRINITY_DN11185_c0_g1_i1.p1 | 5.5 | 6.7E-13 | 1.7 | 2.1E-03 | GDSL esterase/lipase At5g14450-like |
| TRINITY_DN17739_c0_g3_i1.p1 | 2.6 | 7.0E-40 | 2.1 | 1.9E-16 | germin-like protein 5-1 |
| TRINITY_DN17739_c0_g3_i6.p1 | 1.9 | 3.6E-06 | 2.3 | 2.1E-10 | germin-like protein 5-1 |
| TRINITY_DN16013_c2_g5_i1.p1 | 5.4 | 4.0E-22 | 2.2 | 2.6E-07 | germin-like protein subfamily 1 member 13 |
| TRINITY_DN15259_c0_g1_i1.p1 | 7.9 | 3.7E-74 | 5.0 | 2.2E-17 | germin-like protein subfamily T member 2 |
| TRINITY_DN15259_c0_g1_i5.p1 | 5.9 | 3.0E-15 | 8.1 | 7.2E-17 | germin-like protein subfamily T member 2 |
| TRINITY_DN37_c0_g1_i1.p2 | 3.7 | 1.7E-12 | 2.2 | 1.1E-04 | glu S. griseus protease inhibitor-like |
| TRINITY_DN19286_c0_g1_i1.p2 | 5.6 | 1.3E-19 | 3.9 | 1.1E-03 | glu S. griseus protease inhibitor-like |
| TRINITY_DN18572_c1_g3_i2.p1 | 1.9 | 5.7E-11 | 1.2 | 1.7E-05 | glucan endo-1,3-*β*-glucosidase |
| TRINITY_DN20953_c0_g1_i4.p1 | 2.6 | 2.6E-05 | 3.3 | 1.8E-08 | glucan endo-1,3-*β*-glucosidase 8-like |
| TRINITY_DN21815_c0_g4_i1.p1 | 3.6 | 1.6E-17 | 3.7 | 5.9E-10 | glucan endo-1,3-*β*-glucosidase, basic isoform-like |
| TRINITY_DN19278_c0_g1_i10.p1 | 1.1 | 4.8E-06 | 1.0 | 4.0E-04 | glucose-6-phosphate 1-dehydrogenase, cytoplasmic isoform |
| TRINITY_DN15744_c0_g3_i1.p1 | 7.7 | 4.2E-33 | 3.1 | 2.6E-06 | glutamate decarboxylase 1 |
| TRINITY_DN16173_c0_g1_i5.p1 | 2.7 | 5.0E-08 | 2.8 | 8.1E-09 | glutamate receptor 2.8-like |
| TRINITY_DN17492_c0_g1_i2.p1 | 6.2 | 1.7E-28 | 2.5 | 2.2E-08 | glutamate receptor 2.8-like |
| TRINITY_DN18384_c0_g1_i4.p1 | 3.4 | 1.6E-16 | 1.4 | 1.6E-05 | glutamate receptor 2.8-like |
| TRINITY_DN19056_c0_g1_i1.p1 | 4.4 | 3.3E-30 | 2.0 | 2.2E-05 | glutathione S-transferase L3-like |
| TRINITY_DN19120_c2_g5_i1.p1 | 3.9 | 4.5E-13 | 2.7 | 1.1E-06 | glutathione S-transferase U17-like |
| TRINITY_DN18920_c0_g2_i1.p1 | 3.7 | 6.6E-32 | 1.9 | 1.6E-06 | glutathione S-transferase U17-like |
| TRINITY_DN17587_c1_g6_i1.p1 | 2.0 | 2.1E-07 | 2.2 | 3.3E-06 | glutathione S-transferase U17-like |
| TRINITY_DN18606_c0_g2_i3.p1 | 2.3 | 1.1E-10 | 2.0 | 3.4E-06 | glutathione S-transferase U17-like |
| TRINITY_DN18606_c0_g1_i4.p1 | 1.3 | 1.4E-07 | 1.3 | 3.5E-06 | glutathione S-transferase U17-like |
| TRINITY_DN19120_c2_g3_i1.p1 | 4.2 | 5.5E-18 | 2.2 | 5.8E-06 | glutathione S-transferase U17-like |
| TRINITY_DN18920_c0_g5_i10.p1 | 3.5 | 1.0E-11 | 2.3 | 6.4E-06 | glutathione S-transferase U17-like |
| TRINITY_DN11132_c0_g1_i1.p1 | 6.2 | 8.0E-32 | 2.2 | 2.0E-04 | glutathione S-transferase U17-like |
| TRINITY_DN18920_c0_g7_i3.p1 | 1.2 | 8.7E-05 | 1.4 | 2.4E-04 | glutathione S-transferase U17-like |
| TRINITY_DN13591_c0_g1_i2.p1 | 4.1 | 2.6E-44 | 1.3 | 2.9E-07 | glutathione S-transferase U9-like |
| TRINITY_DN13101_c0_g1_i1.p1 | 8.0 | 1.0E-36 | 4.1 | 1.8E-05 | glutathione S-transferase U9-like |
| TRINITY_DN18596_c2_g3_i1.p1 | 1.4 | 5.3E-08 | 1.1 | 1.1E-04 | glutathionyl-hydroquinone reductase YqjG |
| TRINITY_DN14636_c0_g1_i1.p1 | 4.4 | 1.9E-22 | 1.0 | 6.2E-03 | glyceraldehyde-3-phosphate dehydrogenase, cytosolic |
| TRINITY_DN16162_c3_g2_i1.p1 | 3.7 | 5.7E-09 | 2.8 | 2.3E-04 | glycosyltransferase BC10-like |
| TRINITY_DN18563_c0_g2_i4.p1 | 1.3 | 6.1E-07 | 1.0 | 6.4E-04 | glycosyltransferase family protein 64 protein C5 |
| TRINITY_DN10544_c0_g1_i1.p1 | 3.1 | 9.2E-04 | 3.9 | 2.5E-04 | golgin subfamily A member 4 |
| TRINITY_DN20071_c0_g1_i5.p1 | 5.4 | 2.0E-10 | 3.2 | 5.0E-06 | G-type lectin S-receptor-like serine/threonine-protein kinase At1g11330 |
| TRINITY_DN20071_c0_g1_i6.p1 | 3.3 | 1.3E-05 | 2.2 | 4.4E-03 | G-type lectin S-receptor-like serine/threonine-protein kinase At1g11330 |
| TRINITY_DN18234_c1_g1_i1.p1 | 2.5 | 4.6E-03 | 3.3 | 1.5E-06 | G-type lectin S-receptor-like serine/threonine-protein kinase At1g11410 |
| TRINITY_DN21167_c0_g4_i5.p1 | 3.3 | 4.0E-25 | 3.0 | 2.2E-10 | G-type lectin S-receptor-like serine/threonine-protein kinase LECRK3 |
| TRINITY_DN21167_c0_g4_i2.p1 | 1.3 | 5.3E-05 | 1.0 | 6.0E-03 | G-type lectin S-receptor-like serine/threonine-protein kinase LECRK3 |
| TRINITY_DN18970_c0_g1_i5.p1 | 3.6 | 1.5E-11 | 1.6 | 1.5E-03 | G-type lectin S-receptor-like serine/threonine-protein kinase RKS1 |
| TRINITY_DN19826_c2_g1_i6.p1 | 1.8 | 1.8E-05 | 1.3 | 7.8E-04 | G-type lectin S-receptor-like serine/threonine-protein kinase SD2-5 |
| TRINITY_DN14115_c0_g2_i3.p1 | 2.2 | 1.1E-05 | 1.6 | 1.2E-03 | G-type lectin S-receptor-like serine/threonine-protein kinase SD2-5 |
| TRINITY_DN13638_c0_g1_i1.p1 | 2.9 | 1.9E-14 | 1.5 | 4.8E-05 | haloacid dehalogenase-like hydrolase domain-containing protein Sgpp |
| TRINITY_DN15533_c3_g1_i5.p1 | 2.5 | 3.1E-19 | 1.2 | 9.6E-05 | heat shock factor protein HSF24 |
| TRINITY_DN11661_c0_g1_i1.p1 | 8.6 | 3.1E-15 | 4.7 | 1.1E-09 | heat stress transcription factor B-2a-like |
| TRINITY_DN17503_c0_g5_i2.p1 | 8.0 | 1.4E-41 | 1.6 | 2.3E-05 | heat stress transcription factor B-3 |
| TRINITY_DN19703_c4_g1_i13.p1 | 1.3 | 4.6E-03 | 1.4 | 4.3E-03 | heavy metal-associated isoprenylated plant protein 39 |
| TRINITY_DN17019_c1_g1_i3.p1 | 2.1 | 7.4E-06 | 1.7 | 4.7E-04 | heavy metal-associated isoprenylated plant protein 39-like |
| TRINITY_DN21974_c5_g1_i6.p1 | 1.8 | 7.7E-09 | 1.2 | 6.1E-03 | heavy metal-associated isoprenylated plant protein 3-like |
| TRINITY_DN9433_c0_g1_i1.p1 | 4.2 | 3.0E-08 | 4.1 | 6.3E-08 | heavy metal-associated isoprenylated plant protein 43-like |
| TRINITY_DN20021_c0_g2_i1.p1 | 1.1 | 4.4E-03 | 2.3 | 3.9E-06 | heavy metal-associated isoprenylated plant protein 47-like |
| TRINITY_DN12239_c0_g1_i2.p1 | 1.9 | 9.4E-03 | 3.5 | 1.4E-06 | heme-binding protein 2-like |
| TRINITY_DN12541_c0_g1_i1.p1 | 5.2 | 4.0E-35 | 1.7 | 3.2E-09 | hexose carrier protein HEX6-like |
| TRINITY_DN20826_c7_g7_i1.p1 | 2.7 | 3.1E-08 | 1.9 | 9.0E-05 | histone-lysine *N*-methyltransferase trithorax-like protein |
| TRINITY_DN10797_c0_g1_i1.p1 | 3.3 | 7.3E-05 | 3.4 | 2.0E-04 | homeobox protein 9 |
| TRINITY_DN10393_c0_g1_i1.p1 | 1.8 | 8.8E-03 | 3.0 | 2.1E-04 | homeobox-leucine zipper protein HOX3 |
| TRINITY_DN16447_c0_g6_i1.p1 | 4.0 | 4.7E-20 | 2.8 | 4.3E-10 | hydroquinone glucosyltransferase-like |
| TRINITY_DN16447_c0_g3_i3.p1 | 6.6 | 1.3E-06 | 3.4 | 1.4E-03 | hydroquinone glucosyltransferase-like |
| TRINITY_DN18759_c1_g6_i4.p1 | 1.7 | 2.0E-16 | 1.3 | 1.3E-09 | hydroxymethylglutaryl-CoA synthase |
| TRINITY_DN11760_c0_g1_i2.p1 | 7.6 | 2.7E-14 | 2.7 | 9.4E-07 | hyoscyamine 6-dioxygenase-like |
| TRINITY_DN18394_c1_g1_i1.p1 | 4.2 | 6.7E-16 | 2.1 | 1.4E-05 | hypothetical protein BVC80_9011g59 |
| TRINITY_DN17228_c0_g1_i1.p1 | 5.9 | 1.7E-04 | 4.0 | 3.6E-03 | hypothetical protein C3L33_04213, partial |
| TRINITY_DN18394_c1_g2_i1.p1 | 3.4 | 1.3E-05 | 3.0 | 4.2E-03 | hypothetical protein F0562_033058 |
| TRINITY_DN16115_c1_g1_i4.p1 | 3.4 | 2.7E-16 | 2.2 | 1.7E-08 | IAA-amino acid hydrolase ILR1-like 6 |
| TRINITY_DN14051_c0_g1_i5.p1 | 2.9 | 1.7E-09 | 1.2 | 7.5E-03 | IAA-amino acid hydrolase ILR1-like 6 |
| TRINITY_DN16076_c0_g1_i2.p1 | 2.6 | 1.6E-06 | 1.8 | 6.2E-04 | inactive TPR repeat-containing thioredoxin TTL3-like |
| TRINITY_DN16943_c0_g1_i1.p1 | 11.5 | 6.3E-36 | 1.8 | 2.1E-03 | inhibitor of trypsin and hageman factor-like |
| TRINITY_DN21576_c0_g1_i10.p1 | 2.0 | 2.6E-05 | 2.9 | 1.6E-05 | inosine-uridine preferring nucleoside hydrolase family protein |
| TRINITY_DN19277_c0_g4_i1.p1 | 1.3 | 2.9E-06 | 1.4 | 5.8E-06 | internal alternative NAD(P)H-ubiquinone oxidoreductase A1, mitochondrial-like |
| TRINITY_DN19061_c4_g4_i1.p1 | 2.5 | 8.9E-04 | 3.3 | 5.4E-05 | IQ domain-containing protein IQM2-like |
| TRINITY_DN13740_c0_g1_i4.p1 | 4.3 | 2.1E-13 | 3.8 | 1.4E-08 | isochorismate synthase |
| TRINITY_DN15393_c1_g4_i3.p1 | 2.2 | 5.4E-26 | 1.2 | 1.0E-06 | isopentenyl-diphosphate Δ-isomerase I |
| TRINITY_DN9931_c0_g1_i1.p1 | 5.7 | 3.2E-17 | 4.1 | 7.9E-09 | kunitz trypsin inhibitor 2-like |
| TRINITY_DN9371_c0_g1_i1.p1 | 5.2 | 5.8E-44 | 1.2 | 1.9E-04 | laccase-12-like |
| TRINITY_DN21152_c0_g1_i1.p1 | 7.9 | 1.5E-15 | 7.9 | 7.5E-15 | laccase-15-like |
| TRINITY_DN21152_c0_g2_i7.p1 | 6.8 | 1.3E-10 | 6.2 | 7.5E-09 | laccase-15-like |
| TRINITY_DN19024_c0_g2_i3.p1 | 8.4 | 5.2E-50 | 2.5 | 7.7E-07 | laccase-7-like |
| TRINITY_DN12364_c0_g1_i1.p1 | 2.8 | 1.3E-03 | 2.9 | 1.1E-09 | L-ascorbate oxidase |
| TRINITY_DN17284_c0_g5_i1.p1 | 4.9 | 3.5E-08 | 4.0 | 1.6E-06 | L-ascorbate oxidase homolog |
| TRINITY_DN16929_c0_g1_i3.p1 | 4.6 | 2.7E-24 | 1.1 | 2.0E-03 | L-ascorbate oxidase homolog |
| TRINITY_DN16929_c0_g3_i1.p1 | 1.2 | 5.6E-04 | 1.1 | 3.4E-03 | L-ascorbate oxidase homolog |
| TRINITY_DN15463_c0_g1_i3.p1 | 1.3 | 2.7E-06 | 1.0 | 6.6E-04 | L-aspartate oxidase |
| TRINITY_DN12859_c0_g1_i1.p1 | 2.7 | 7.2E-05 | 2.2 | 9.5E-07 | late embryogenesis abundant protein At1g64065-like |
| TRINITY_DN16294_c1_g4_i1.p1 | 3.3 | 7.3E-30 | 1.2 | 1.3E-06 | late embryogenesis abundant protein At1g64065-like |
| TRINITY_DN10566_c0_g1_i1.p1 | 3.0 | 5.2E-16 | 1.4 | 1.9E-06 | late embryogenesis abundant protein At1g64065-like |
| TRINITY_DN21044_c1_g1_i12.p1 | 3.7 | 2.5E-08 | 3.1 | 2.4E-06 | late embryogenesis abundant protein At1g64065-like |
| TRINITY_DN9586_c0_g1_i1.p1 | 3.2 | 2.3E-06 | 2.4 | 4.3E-04 | late embryogenesis abundant protein At1g64065-like |
| TRINITY_DN21524_c0_g3_i2.p1 | 1.9 | 4.0E-07 | 2.2 | 3.2E-07 | LEAF RUST 10 DISEASE-RESISTANCE LOCUS RECEPTOR-LIKE PROTEIN KINASE-like 1.2 |
| TRINITY_DN20544_c0_g2_i4.p1 | 6.3 | 2.8E-15 | 3.5 | 3.7E-08 | LEAF RUST 10 DISEASE-RESISTANCE LOCUS RECEPTOR-LIKE PROTEIN KINASE-like 2.1 |
| TRINITY_DN17838_c0_g5_i2.p1 | 3.0 | 2.7E-09 | 2.9 | 1.2E-07 | LEAF RUST 10 DISEASE-RESISTANCE LOCUS RECEPTOR-LIKE PROTEIN KINASE-like 2.1 |
| TRINITY_DN14793_c0_g3_i1.p1 | 6.8 | 7.5E-12 | 3.1 | 3.8E-05 | LRR receptor-like serine/threonine-protein kinase IOS1 |
| TRINITY_DN13577_c0_g1_i2.p1 | 2.9 | 4.8E-21 | 1.3 | 2.2E-04 | lysine histidine transporter 1-like |
| TRINITY_DN11253_c0_g1_i2.p1 | 4.6 | 5.2E-30 | 3.1 | 3.0E-13 | lysine histidine transporter-like 8 |
| TRINITY_DN14292_c0_g1_i1.p1 | 2.8 | 3.4E-07 | 2.0 | 5.4E-04 | lysine ketoglutarate reductase trans-splicing protein |
| TRINITY_DN13590_c0_g1_i1.p1 | 3.5 | 2.6E-09 | 2.1 | 3.0E-04 | MACPF domain-containing protein At1g14780 |
| TRINITY_DN16312_c0_g4_i1.p1 | 10.2 | 3.0E-62 | 3.5 | 7.3E-17 | macrophage migration inhibitory factor homolog |
| TRINITY_DN21192_c2_g1_i1.p1 | 6.5 | 1.3E-17 | 3.4 | 1.5E-08 | major allergen Pru ar 1-like |
| TRINITY_DN14812_c0_g1_i2.p1 | 4.8 | 5.4E-27 | 2.4 | 4.7E-07 | major allergen Pru ar 1-like |
| TRINITY_DN14812_c0_g2_i2.p1 | 10.6 | 1.6E-68 | 3.0 | 2.8E-05 | major allergen Pru ar 1-like |
| TRINITY_DN7107_c0_g1_i1.p1 | 5.5 | 1.2E-16 | 2.2 | 1.2E-04 | major allergen Pru ar 1-like |
| TRINITY_DN14812_c0_g1_i1.p1 | 4.1 | 3.8E-18 | 3.4 | 5.3E-04 | major allergen Pru ar 1-like |
| TRINITY_DN14812_c0_g4_i2.p1 | 10.9 | 1.0E-42 | 3.6 | 9.6E-04 | major allergen Pru ar 1-like |
| TRINITY_DN19085_c1_g1_i1.p1 | 1.0 | 1.0E-04 | 1.2 | 4.6E-05 | metal transporter Nramp5-like |
| TRINITY_DN20660_c0_g1_i5.p1 | 2.3 | 1.3E-13 | 1.4 | 4.3E-04 | methylecgonone reductase-like |
| TRINITY_DN20133_c1_g2_i16.p1 | 1.9 | 3.0E-05 | 2.5 | 6.8E-08 | methyltransferase-like protein 7A |
| TRINITY_DN10208_c0_g1_i1.p1 | 6.2 | 2.7E-05 | 3.5 | 2.6E-03 | methyltransferase-related protein |
| TRINITY_DN11489_c0_g2_i1.p1 | 4.6 | 9.7E-07 | 4.3 | 7.9E-07 | miraculin-like |
| TRINITY_DN15365_c0_g1_i1.p1 | 9.2 | 5.5E-12 | 3.6 | 3.3E-05 | mitochondrial phosphate carrier protein 3, mitochondrial-like |
| TRINITY_DN12534_c0_g2_i1.p1 | 1.9 | 1.3E-03 | 2.1 | 3.2E-05 | mitogen-activated protein kinase kinase kinase 17-like |
| TRINITY_DN19109_c1_g1_i1.p1 | 2.8 | 6.1E-20 | 1.6 | 7.0E-07 | MLO-like protein 1 |
| TRINITY_DN15652_c1_g1_i8.p1 | 1.4 | 4.7E-05 | 1.2 | 1.5E-03 | MLO-like protein 6 |
| TRINITY_DN17851_c1_g4_i1.p1 | 4.2 | 1.5E-14 | 2.2 | 1.7E-05 | molybdenum cofactor sulfurase |
| TRINITY_DN14412_c0_g2_i1.p1 | 2.2 | 9.0E-06 | 2.4 | 1.1E-05 | monooxygenase 2 isoform X2 |
| TRINITY_DN17216_c1_g1_i9.p1 | 3.4 | 2.5E-17 | 2.2 | 6.0E-09 | monooxygenase 2-like |
| TRINITY_DN19317_c1_g5_i1.p1 | 4.1 | 1.1E-21 | 2.3 | 8.6E-09 | monooxygenase 2-like |
| TRINITY_DN20896_c1_g1_i3.p1 | 7.1 | 3.3E-14 | 3.9 | 1.5E-08 | monooxygenase 2-like |
| TRINITY_DN20896_c1_g3_i1.p1 | 9.5 | 3.0E-17 | 4.2 | 3.0E-08 | monooxygenase 2-like |
| TRINITY_DN17216_c1_g2_i2.p1 | 6.8 | 1.3E-12 | 4.0 | 1.1E-06 | monooxygenase 2-like |
| TRINITY_DN19317_c1_g2_i1.p1 | 4.6 | 5.5E-24 | 1.9 | 1.4E-06 | monooxygenase 2-like |
| TRINITY_DN20896_c1_g3_i2.p1 | 6.2 | 1.2E-16 | 2.8 | 2.5E-06 | monooxygenase 2-like |
| TRINITY_DN19317_c0_g1_i2.p1 | 4.6 | 1.7E-03 | 7.3 | 2.9E-05 | monooxygenase 2-like |
| TRINITY_DN20896_c1_g2_i2.p1 | 4.4 | 3.7E-17 | 1.8 | 2.0E-03 | monooxygenase 2-like |
| TRINITY_DN17811_c1_g6_i1.p1 | 8.8 | 2.1E-113 | 2.5 | 7.9E-27 | myb-related protein Myb4-like |
| TRINITY_DN17241_c4_g1_i1.p1 | 3.0 | 1.9E-12 | 2.3 | 2.8E-06 | myb-related protein Myb4-like |
| TRINITY_DN20808_c0_g2_i3.p1 | 5.1 | 1.0E-35 | 7.5 | 9.4E-54 | ---NA***--- |
| TRINITY_DN20229_c1_g1_i2.p2 | 1.2 | 1.8E-10 | 2.4 | 8.8E-29 | ---NA--- |
| TRINITY_DN17290_c3_g2_i10.p1 | 3.5 | 2.4E-24 | 3.3 | 4.0E-19 | ---NA--- |
| TRINITY_DN16569_c2_g8_i1.p1 | 7.4 | 6.0E-120 | 1.8 | 1.4E-12 | ---NA--- |
| TRINITY_DN26517_c0_g1_i1.p1 | 5.5 | 2.1E-22 | 3.1 | 5.2E-09 | ---NA--- |
| TRINITY_DN8559_c0_g1_i1.p1 | 2.8 | 1.2E-08 | 3.1 | 1.4E-08 | ---NA--- |
| TRINITY_DN20598_c3_g2_i1.p1 | 7.7 | 4.5E-07 | 7.7 | 1.9E-06 | ---NA--- |
| TRINITY_DN18525_c1_g2_i1.p1 | 2.5 | 1.3E-11 | 1.9 | 5.8E-06 | ---NA--- |
| TRINITY_DN21519_c0_g4_i2.p1 | 3.9 | 2.2E-12 | 2.2 | 1.3E-05 | ---NA--- |
| TRINITY_DN19120_c2_g3_i3.p1 | 5.0 | 1.3E-10 | 3.0 | 1.3E-05 | ---NA--- |
| TRINITY_DN19021_c0_g6_i1.p1 | 3.1 | 8.8E-05 | 3.9 | 1.5E-05 | ---NA--- |
| TRINITY_DN9176_c0_g1_i1.p1 | 5.0 | 4.1E-08 | 3.2 | 1.7E-04 | ---NA--- |
| TRINITY_DN18438_c1_g5_i3.p1 | 2.4 | 3.3E-05 | 2.3 | 1.7E-04 | ---NA--- |
| TRINITY_DN18682_c0_g1_i6.p1 | 3.2 | 3.8E-14 | 1.3 | 2.9E-04 | ---NA--- |
| TRINITY_DN15393_c1_g4_i9.p4 | 2.3 | 1.9E-04 | 2.1 | 8.3E-04 | ---NA--- |
| TRINITY_DN16359_c0_g5_i1.p1 | 6.6 | 2.0E-07 | 3.0 | 8.6E-04 | ---NA--- |
| TRINITY_DN20707_c0_g2_i3.p2 | 7.0 | 1.3E-08 | 2.4 | 1.5E-03 | ---NA--- |
| TRINITY_DN17420_c1_g1_i1.p1 | 6.9 | 2.0E-05 | 4.1 | 1.8E-03 | ---NA--- |
| TRINITY_DN21597_c3_g1_i3.p1 | 3.6 | 9.3E-04 | 3.4 | 2.0E-03 | ---NA--- |
| TRINITY_DN17420_c1_g4_i1.p1 | 3.3 | 2.7E-03 | 3.9 | 3.4E-03 | ---NA--- |
| TRINITY_DN37033_c0_g1_i1.p1 | 4.1 | 1.8E-11 | 1.5 | 6.1E-03 | ---NA--- |
| TRINITY_DN13704_c0_g1_i1.p1 | 3.9 | 3.4E-03 | 3.8 | 9.5E-03 | ---NA--- |
| TRINITY_DN20956_c1_g10_i2.p1 | 2.4 | 1.0E-12 | 2.7 | 5.1E-18 | NAC domain-containing protein 2-like |
| TRINITY_DN20956_c1_g10_i1.p1 | 2.4 | 5.2E-07 | 2.8 | 5.8E-09 | NAC domain-containing protein 2-like |
| TRINITY_DN19333_c0_g1_i1.p1 | 7.0 | 4.1E-43 | 1.6 | 1.2E-07 | NAD/NADP octopine/nopaline dehydrogenase family protein |
| TRINITY_DN14697_c0_g1_i1.p1 | 2.7 | 9.8E-16 | 1.9 | 5.3E-08 | NADP-dependent malic enzyme |
| TRINITY_DN16987_c0_g2_i2.p1 | 2.2 | 8.6E-31 | 1.0 | 1.4E-06 | NADPH:quinone oxidoreductase-like |
| TRINITY_DN18476_c0_g1_i6.p1 | 2.2 | 5.1E-22 | 1.3 | 6.2E-06 | NADPH-cytochrome P450 reductase-like |
| TRINITY_DN36168_c0_g1_i1.p1 | 2.1 | 3.1E-15 | 1.4 | 1.7E-06 | NADPH-dependent aldehyde reductase-like protein, chloroplastic |
| TRINITY_DN12719_c0_g1_i1.p1 | 5.0 | 2.0E-27 | 2.0 | 6.8E-07 | NDR1/HIN1-like protein 6 |
| TRINITY_DN15803_c0_g1_i2.p1 | 2.8 | 1.4E-20 | 1.2 | 2.0E-03 | nitronate monooxygenase |
| TRINITY_DN12598_c1_g1_i4.p1 | 5.8 | 1.2E-09 | 4.4 | 2.3E-07 | nodulation protein H |
| TRINITY_DN16996_c0_g4_i6.p1 | 5.4 | 3.6E-29 | 2.4 | 1.5E-06 | non-functional NADPH-dependent codeinone reductase 2-like |
| TRINITY_DN16503_c3_g1_i2.p1 | 3.4 | 1.1E-14 | 2.0 | 2.0E-05 | non-functional NADPH-dependent codeinone reductase 2-like |
| TRINITY_DN10689_c0_g1_i1.p1 | 6.6 | 2.6E-05 | 3.3 | 4.8E-03 | non-specific lipid-transfer protein 1-like |
| TRINITY_DN10205_c0_g1_i1.p1 | 2.5 | 3.5E-14 | 3.5 | 4.8E-30 | non-specific lipid-transfer protein 2-like |
| TRINITY_DN10353_c0_g1_i1.p1 | 4.3 | 2.9E-15 | 2.1 | 4.5E-06 | non-specific phospholipase C2 |
| TRINITY_DN14771_c0_g1_i2.p1 | 3.2 | 2.2E-03 | 3.6 | 1.2E-05 | nuclear valosin-containing protein |
| TRINITY_DN12348_c0_g1_i1.p1 | 3.7 | 1.2E-18 | 1.1 | 7.6E-03 | NUDIX domain-containing protein |
| TRINITY_DN18504_c0_g1_i10.p1 | 1.8 | 3.2E-11 | 2.4 | 6.1E-15 | *O*-glucosyltransferase rumi homolog |
| TRINITY_DN17194_c1_g1_i1.p1 | 4.4 | 3.2E-13 | 3.1 | 2.8E-06 | *O*-glucosyltransferase rumi homolog |
| TRINITY_DN11505_c0_g1_i1.p1 | 2.3 | 3.7E-04 | 2.4 | 9.7E-09 | PAR1 protein |
| TRINITY_DN19998_c3_g1_i1.p1 | 6.1 | 1.4E-29 | 3.3 | 2.0E-07 | PAR1 protein |
| TRINITY_DN13035_c0_g1_i1.p1 | 1.6 | 2.1E-04 | 1.4 | 5.1E-03 | patatin-like protein 2 |
| TRINITY_DN6017_c0_g1_i1.p1 | 5.9 | 2.2E-03 | 5.9 | 5.1E-03 | patatin-like protein 2 |
| TRINITY_DN12610_c0_g1_i1.p1 | 2.7 | 5.5E-08 | 2.2 | 2.0E-06 | pathogen-associated molecular patterns-induced protein A70 |
| TRINITY_DN17418_c2_g4_i1.p1 | 3.2 | 8.0E-12 | 3.2 | 8.1E-11 | pathogenesis-related genes transcriptional activator PTI5-like |
| TRINITY_DN42377_c0_g1_i1.p1 | 2.2 | 2.1E-07 | 3.9 | 1.9E-18 | pathogenesis-related protein 1 |
| TRINITY_DN15340_c0_g4_i1.p1 | 4.8 | 1.5E-08 | 2.5 | 5.5E-03 | pathogenesis-related protein 1 |
| TRINITY_DN15340_c0_g6_i1.p1 | 5.4 | 5.5E-12 | 3.0 | 5.0E-04 | pathogenesis-related protein 1-like |
| TRINITY_DN19041_c1_g1_i13.p1 | 4.8 | 6.2E-42 | 2.0 | 1.3E-10 | pathogen-related protein-like |
| TRINITY_DN16917_c0_g1_i3.p1 | 2.5 | 4.9E-22 | 1.3 | 4.3E-05 | pathogen-related protein-like |
| TRINITY_DN19041_c1_g1_i10.p1 | 4.3 | 3.1E-16 | 1.9 | 1.3E-04 | pathogen-related protein-like |
| TRINITY_DN18738_c5_g4_i2.p1 | 8.2 | 1.6E-19 | 2.7 | 1.5E-05 | pectinesterase 2-like |
| TRINITY_DN15791_c1_g5_i1.p1 | 11.9 | 2.1E-35 | 3.5 | 1.3E-03 | pectinesterase 2-like |
| TRINITY_DN9929_c0_g1_i1.p1 | 10.8 | 3.0E-22 | 2.5 | 5.6E-03 | pectinesterase 2-like |
| TRINITY_DN21761_c2_g1_i1.p1 | 7.4 | 9.7E-98 | 3.9 | 2.1E-23 | pectinesterase-like |
| TRINITY_DN21761_c2_g6_i1.p1 | 6.3 | 5.4E-15 | 3.1 | 3.5E-07 | pectinesterase-like |
| TRINITY_DN20902_c0_g1_i6.p1 | 4.3 | 7.8E-04 | 4.2 | 2.3E-03 | pentatricopeptide repeat-containing protein |
| TRINITY_DN12658_c0_g1_i1.p1 | 9.0 | 1.4E-81 | 2.6 | 2.6E-18 | peroxidase 24-like |
| TRINITY_DN10333_c0_g1_i2.p1 | 3.3 | 4.9E-25 | 1.8 | 9.1E-10 | peroxidase 4-like |
| TRINITY_DN13697_c1_g1_i1.p1 | 6.5 | 2.1E-45 | 1.4 | 3.5E-04 | peroxidase N1-like |
| TRINITY_DN18233_c0_g1_i1.p1 | 4.0 | 1.1E-39 | 2.1 | 1.8E-22 | peroxidase P7-like |
| TRINITY_DN10489_c0_g1_i1.p1 | 3.7 | 2.9E-27 | 1.1 | 4.4E-04 | peroxidase P7-like |
| TRINITY_DN21062_c3_g1_i9.p1 | 6.9 | 3.5E-29 | 3.6 | 1.7E-11 | peroxisomal membrane protein 2 |
| TRINITY_DN11843_c0_g1_i1.p1 | 3.6 | 3.6E-48 | 1.1 | 2.4E-06 | phenylalanine ammonia-lyase |
| TRINITY_DN20613_c2_g1_i2.p1 | 1.8 | 9.8E-11 | 1.1 | 2.3E-03 | phospho-2-dehydro-3-deoxyheptonate aldolase 2, chloroplastic-like |
| TRINITY_DN15145_c0_g5_i1.p1 | 1.5 | 4.7E-08 | 1.7 | 3.2E-09 | phospholipase A1-IIΔ |
| TRINITY_DN16642_c0_g4_i2.p1 | 1.2 | 3.2E-06 | 1.2 | 3.1E-05 | phospholipid-transporting ATPase 1 |
| TRINITY_DN10577_c0_g1_i1.p1 | 2.1 | 1.5E-11 | 1.4 | 2.2E-05 | plastid movement impaired protein |
| TRINITY_DN12301_c0_g1_i1.p1 | 8.2 | 2.3E-16 | 3.5 | 1.8E-09 | plastocyanin-like protein |
| TRINITY_DN16829_c1_g6_i1.p1 | 8.8 | 1.1E-14 | 5.1 | 1.8E-08 | pleiotropic drug resistance protein 1-like |
| TRINITY_DN17445_c1_g1_i15.p1 | 2.5 | 4.7E-04 | 2.6 | 7.6E-04 | pleiotropic drug resistance protein 1-like |
| TRINITY_DN14521_c0_g2_i2.p1 | 2.7 | 1.5E-24 | 2.0 | 1.1E-13 | pleiotropic drug resistance protein 2-like |
| TRINITY_DN20279_c0_g3_i7.p1 | 1.3 | 2.4E-04 | 1.7 | 5.6E-10 | pleiotropic drug resistance protein 3-like |
| TRINITY_DN10449_c0_g1_i1.p1 | 5.3 | 2.1E-36 | 3.5 | 7.5E-15 | polyadenylate-binding protein 1-B-binding protein |
| TRINITY_DN19774_c0_g5_i1.p1 | 7.2 | 7.8E-50 | 2.6 | 5.7E-07 | polygalacturonase inhibitor-like |
| TRINITY_DN15037_c0_g1_i2.p1 | 5.3 | 1.9E-50 | 2.2 | 8.2E-06 | polygalacturonase inhibitor-like |
| TRINITY_DN18401_c2_g2_i4.p1 | 3.4 | 1.1E-50 | 1.3 | 8.0E-05 | polygalacturonase inhibitor-like |
| TRINITY_DN15037_c0_g1_i1.p1 | 3.4 | 5.3E-43 | 1.3 | 6.2E-04 | polygalacturonase inhibitor-like |
| TRINITY_DN19603_c0_g2_i4.p1 | 8.1 | 2.2E-25 | 6.6 | 6.2E-21 | polyphenol oxidase, chloroplastic-like |
| TRINITY_DN21674_c0_g2_i2.p1 | 5.6 | 1.6E-13 | 8.2 | 2.1E-15 | polyphenol oxidase, chloroplastic-like |
| TRINITY_DN19603_c0_g4_i1.p1 | 2.5 | 5.1E-03 | 3.5 | 1.1E-03 | polyphenol oxidase, chloroplastic-like |
| TRINITY_DN21674_c0_g1_i4.p1 | 1.6 | 3.2E-03 | 1.8 | 6.0E-03 | polyphenol oxidase, chloroplastic-like |
| TRINITY_DN14492_c0_g1_i2.p1 | 7.8 | 7.5E-44 | 3.1 | 1.0E-15 | potassium transporter 5-like |
| TRINITY_DN18960_c1_g1_i4.p1 | 1.1 | 1.8E-03 | 1.3 | 7.2E-04 | PRA1 family protein F4-like |
| TRINITY_DN19628_c0_g7_i1.p1 | 8.1 | 1.1E-25 | 3.6 | 7.2E-09 | premnaspirodiene oxygenase-like |
| TRINITY_DN15091_c0_g1_i1.p1 | 3.3 | 5.7E-08 | 1.8 | 9.7E-04 | probable 1-acyl-sn-glycerol-3-phosphate acyltransferase 5 |
| TRINITY_DN12094_c0_g1_i1.p1 | 3.2 | 3.7E-21 | 2.7 | 8.5E-09 | probable 1-deoxy-D-xylulose-5-phosphate synthase 2, chloroplastic |
| TRINITY_DN20963_c0_g1_i2.p1 | 1.7 | 6.5E-07 | 1.4 | 7.3E-04 | probable 6-phosphogluconolactonase 4, chloroplastic |
| TRINITY_DN11404_c0_g1_i1.p1 | 6.2 | 7.5E-15 | 4.0 | 5.7E-08 | probable acyl-activating enzyme 1, peroxisomal |
| TRINITY_DN11711_c0_g1_i4.p1 | 1.6 | 1.4E-09 | 1.6 | 2.6E-08 | probable aldo-keto reductase 1 |
| TRINITY_DN19629_c1_g1_i1.p1 | 5.2 | 7.2E-62 | 2.3 | 1.4E-21 | probable aldo-keto reductase 2 |
| TRINITY_DN17625_c1_g5_i1.p2 | 4.8 | 7.2E-14 | 2.9 | 3.6E-07 | probable aldo-keto reductase 2 |
| TRINITY_DN19629_c1_g7_i3.p1 | 3.2 | 6.2E-44 | 1.4 | 3.9E-07 | probable aldo-keto reductase 2 |
| TRINITY_DN19662_c1_g3_i3.p1 | 2.2 | 4.4E-18 | 1.1 | 4.4E-05 | probable aldo-keto reductase 2 |
| TRINITY_DN17625_c0_g1_i3.p1 | 1.9 | 2.4E-07 | 1.3 | 1.7E-03 | probable aldo-keto reductase 2 |
| TRINITY_DN17358_c1_g1_i1.p1 | 3.1 | 1.6E-22 | 1.0 | 1.9E-03 | probable aldo-keto reductase 2 |
| TRINITY_DN16771_c1_g3_i2.p1 | 3.4 | 1.5E-20 | 1.5 | 3.2E-07 | probable aquaporin PIP2-5 |
| TRINITY_DN19322_c0_g1_i12.p1 | 3.6 | 7.8E-35 | 2.6 | 4.7E-14 | probable caffeoyl-CoA O-methyltransferase At4g26220 |
| TRINITY_DN19322_c0_g1_i5.p1 | 3.1 | 3.7E-35 | 1.7 | 5.8E-09 | probable caffeoyl-CoA O-methyltransferase At4g26220 |
| TRINITY_DN13064_c0_g1_i1.p1 | 4.0 | 3.2E-25 | 1.8 | 7.5E-09 | probable carboxylesterase 2 |
| TRINITY_DN20644_c2_g1_i1.p1 | 3.2 | 2.7E-31 | 1.8 | 2.7E-08 | probable carotenoid cleavage dioxygenase 4, chloroplastic |
| TRINITY_DN17948_c3_g1_i9.p1 | 3.0 | 2.9E-24 | 1.8 | 2.0E-06 | probable cinnamyl alcohol dehydrogenase 1 |
| TRINITY_DN17948_c3_g1_i11.p1 | 3.2 | 1.0E-08 | 2.0 | 2.8E-04 | probable cinnamyl alcohol dehydrogenase 1 |
| TRINITY_DN11074_c0_g1_i1.p1 | 7.3 | 4.7E-07 | 3.7 | 6.9E-04 | probable flavin-containing monooxygenase 1 |
| TRINITY_DN17179_c0_g1_i1.p1 | 4.7 | 4.4E-12 | 2.1 | 1.5E-04 | probable folate-biopterin transporter 2 |
| TRINITY_DN17179_c0_g1_i3.p1 | 2.1 | 3.2E-03 | 2.6 | 2.3E-03 | probable folate-biopterin transporter 2 |
| TRINITY_DN14259_c0_g1_i5.p1 | 3.6 | 1.4E-29 | 1.4 | 5.8E-06 | probable fructokinase-7 |
| TRINITY_DN10583_c0_g1_i1.p1 | 3.2 | 1.2E-25 | 2.0 | 6.8E-10 | probable galacturonosyltransferase-like 1 |
| TRINITY_DN16812_c0_g7_i1.p1 | 5.3 | 1.9E-45 | 2.4 | 4.2E-17 | probable glucan 1,3-beta-glucosidase A |
| TRINITY_DN16601_c1_g1_i1.p1 | 6.2 | 1.0E-19 | 4.3 | 2.7E-12 | probable glucan 1,3-beta-glucosidase A |
| TRINITY_DN12353_c0_g1_i3.p1 | 4.4 | 7.9E-51 | 3.2 | 3.0E-26 | probable glutathione S-transferase |
| TRINITY_DN15755_c0_g1_i1.p1 | 1.5 | 1.1E-03 | 2.2 | 7.5E-16 | probable glutathione S-transferase |
| TRINITY_DN15723_c0_g3_i2.p1 | 10.3 | 4.9E-90 | 2.9 | 3.0E-08 | probable glutathione S-transferase |
| TRINITY_DN15755_c1_g2_i1.p1 | 2.6 | 2.7E-10 | 2.2 | 9.4E-06 | probable glutathione S-transferase |
| TRINITY_DN15723_c0_g3_i3.p1 | 8.1 | 2.3E-28 | 3.3 | 5.0E-05 | probable glutathione S-transferase |
| TRINITY_DN11060_c0_g1_i1.p1 | 3.2 | 7.8E-12 | 1.5 | 1.9E-03 | probable glutathione S-transferase |
| TRINITY_DN15755_c1_g3_i1.p1 | 2.4 | 8.2E-08 | 1.8 | 4.8E-03 | probable glutathione S-transferase |
| TRINITY_DN15755_c1_g3_i2.p1 | 5.9 | 1.2E-13 | 1.8 | 5.9E-03 | probable glutathione S-transferase |
| TRINITY_DN16952_c0_g1_i1.p1 | 2.0 | 4.3E-03 | 2.5 | 1.8E-03 | probable inactive purple acid phosphatase 27 |
| TRINITY_DN12702_c0_g1_i1.p1 | 6.1 | 3.7E-20 | 4.3 | 5.3E-13 | probable indole-3-acetic acid-amido synthetase GH3.1 |
| TRINITY_DN15215_c0_g2_i1.p1 | 7.9 | 9.7E-12 | 5.2 | 5.2E-09 | probable indole-3-acetic acid-amido synthetase GH3.1 |
| TRINITY_DN17611_c0_g3_i1.p1 | 1.6 | 2.4E-05 | 1.1 | 5.2E-03 | probable LIM domain-containing serine/threonine-protein kinase DDB_G0287001 |
| TRINITY_DN18959_c0_g2_i3.p1 | 4.7 | 4.4E-37 | 2.6 | 8.2E-18 | probable linoleate 9S-lipoxygenase 5 |
| TRINITY_DN18959_c0_g4_i5.p1 | 1.0 | 5.8E-07 | 1.7 | 7.3E-13 | probable linoleate 9S-lipoxygenase 5 |
| TRINITY_DN14767_c0_g1_i1.p1 | 6.1 | 4.3E-16 | 1.6 | 2.9E-03 | probable LRR receptor-like serine/threonine-protein kinase At1g74360 |
| TRINITY_DN21219_c0_g1_i2.p1 | 4.4 | 1.1E-09 | 2.4 | 1.2E-04 | probable LRR receptor-like serine/threonine-protein kinase At2g16250 |
| TRINITY_DN18095_c3_g1_i1.p1 | 2.7 | 1.7E-06 | 1.5 | 2.8E-03 | probable LRR receptor-like serine/threonine-protein kinase At2g16250 |
| TRINITY_DN21219_c0_g1_i9.p1 | 1.5 | 1.2E-07 | 1.4 | 5.6E-03 | probable LRR receptor-like serine/threonine-protein kinase At2g16250 |
| TRINITY_DN17997_c0_g1_i3.p1 | 7.1 | 8.1E-28 | 2.6 | 7.8E-08 | probable LRR receptor-like serine/threonine-protein kinase At3g47570 |
| TRINITY_DN11143_c0_g1_i1.p1 | 5.1 | 4.5E-07 | 5.0 | 2.1E-06 | probable LRR receptor-like serine/threonine-protein kinase At3g47570 |
| TRINITY_DN14687_c0_g4_i4.p1 | 4.9 | 3.6E-11 | 2.9 | 2.5E-06 | probable LRR receptor-like serine/threonine-protein kinase At3g47570 |
| TRINITY_DN8180_c0_g1_i1.p1 | 3.1 | 3.6E-06 | 3.0 | 2.2E-05 | probable LRR receptor-like serine/threonine-protein kinase At3g47570 |
| TRINITY_DN20553_c1_g2_i1.p1 | 8.0 | 7.2E-116 | 2.7 | 1.7E-20 | probable NAD(P)H dehydrogenase (quinone) FQR1-like 1 |
| TRINITY_DN20553_c1_g1_i7.p1 | 7.1 | 1.8E-72 | 2.7 | 3.4E-16 | probable NAD(P)H dehydrogenase (quinone) FQR1-like 1 |
| TRINITY_DN19716_c4_g1_i4.p1 | 9.7 | 2.1E-107 | 2.8 | 1.8E-14 | probable NAD(P)H dehydrogenase (quinone) FQR1-like 1 |
| TRINITY_DN20553_c1_g1_i10.p1 | 4.8 | 8.2E-29 | 2.4 | 4.3E-05 | probable NAD(P)H dehydrogenase (quinone) FQR1-like 1 |
| TRINITY_DN20553_c1_g1_i2.p1 | 9.0 | 9.4E-45 | 3.8 | 1.9E-04 | probable NAD(P)H dehydrogenase (quinone) FQR1-like 1 |
| TRINITY_DN19716_c4_g1_i5.p1 | 9.2 | 5.3E-27 | 2.3 | 1.8E-03 | probable NAD(P)H dehydrogenase (quinone) FQR1-like 1 |
| TRINITY_DN10760_c0_g1_i1.p1 | 4.6 | 1.7E-24 | 1.7 | 1.8E-06 | probable pectate lyase 5 |
| TRINITY_DN18907_c0_g1_i3.p1 | 3.6 | 1.6E-11 | 1.7 | 1.9E-04 | probable pectin methylesterase CGR3 |
| TRINITY_DN11767_c0_g1_i1.p1 | 8.6 | 2.6E-84 | 2.7 | 1.7E-23 | probable pectinesterase/pectinesterase inhibitor 7 |
| TRINITY_DN16931_c1_g3_i1.p1 | 11.1 | 3.0E-78 | 2.4 | 3.2E-08 | probable pectinesterase/pectinesterase inhibitor 7 |
| TRINITY_DN16931_c1_g1_i1.p1 | 6.8 | 1.4E-05 | 4.1 | 1.3E-03 | probable pectinesterase/pectinesterase inhibitor 7 |
| TRINITY_DN16252_c1_g2_i7.p1 | 3.2 | 5.8E-24 | 1.0 | 3.5E-04 | probable phospholipid-transporting ATPase 4 |
| TRINITY_DN11524_c0_g1_i1.p1 | 2.1 | 1.6E-10 | 1.6 | 2.7E-06 | probable pyridoxal 5'-phosphate synthase subunit PDX1 |
| TRINITY_DN12715_c0_g1_i1.p1 | 4.6 | 4.7E-15 | 1.9 | 2.0E-03 | probable receptor-like protein kinase At5g61350 |
| TRINITY_DN17291_c0_g1_i2.p1 | 1.3 | 1.0E-05 | 2.8 | 3.9E-20 | probable receptor-like serine/threonine-protein kinase At5g57670 |
| TRINITY_DN5797_c0_g1_i1.p1 | 6.3 | 1.0E-08 | 3.2 | 4.5E-04 | probable S-adenosylmethionine-dependent methyltransferase At5g38100 |
| TRINITY_DN20314_c0_g2_i42.p1 | 1.5 | 2.9E-04 | 1.2 | 1.3E-03 | probable serine incorporator |
| TRINITY_DN17978_c1_g3_i3.p1 | 3.8 | 4.9E-17 | 1.7 | 3.6E-04 | probable serine/threonine-protein kinase PBL3 |
| TRINITY_DN21493_c0_g1_i5.p1 | 3.6 | 4.4E-11 | 1.5 | 9.7E-03 | probable serine/threonine-protein kinase PBL3 |
| TRINITY_DN10335_c0_g1_i1.p1 | 6.7 | 3.7E-33 | 1.7 | 2.4E-05 | probable serine/threonine-protein kinase PIX13 |
| TRINITY_DN15632_c0_g1_i12.p1 | 1.5 | 4.1E-14 | 1.3 | 9.6E-10 | probable serine/threonine-protein kinase WNK4 isoform X1 |
| TRINITY_DN14957_c0_g1_i2.p1 | 3.1 | 1.8E-22 | 1.4 | 2.8E-06 | probable sulfate transporter 3.5 |
| TRINITY_DN17244_c1_g2_i2.p1 | 1.6 | 2.9E-03 | 2.5 | 1.6E-04 | probable trehalase |
| TRINITY_DN17406_c0_g4_i1.p1 | 3.6 | 1.8E-09 | 1.3 | 5.7E-06 | probable WRKY transcription factor 31 |
| TRINITY_DN16673_c0_g1_i4.p1 | 4.9 | 1.5E-17 | 2.6 | 1.1E-06 | probable WRKY transcription factor 40 |
| TRINITY_DN14729_c0_g1_i3.p1 | 1.7 | 2.2E-03 | 1.8 | 3.0E-05 | probable WRKY transcription factor 40 |
| TRINITY_DN17681_c3_g1_i2.p1 | 3.6 | 9.7E-23 | 2.0 | 9.9E-09 | probable WRKY transcription factor 48 |
| TRINITY_DN14761_c0_g7_i1.p1 | 5.8 | 2.1E-41 | 1.1 | 1.5E-04 | probable WRKY transcription factor 71 |
| TRINITY_DN20922_c1_g1_i2.p1 | 6.5 | 4.6E-37 | 1.6 | 1.7E-04 | probable WRKY transcription factor 75 |
| TRINITY_DN18649_c0_g4_i2.p1 | 2.4 | 7.9E-07 | 1.7 | 1.8E-03 | probably inactive leucine-rich repeat receptor-like protein kinase At5g48380 |
| TRINITY_DN19339_c0_g1_i15.p1 | 4.4 | 7.0E-06 | 3.4 | 2.0E-04 | protein ACCELERATED CELL DEATH 6-like |
| TRINITY_DN22134_c2_g1_i11.p1 | 2.6 | 8.3E-05 | 1.9 | 1.3E-03 | protein argonaute 4-like |
| TRINITY_DN16469_c3_g4_i1.p1 | 1.8 | 1.0E-03 | 3.1 | 1.1E-13 | protein ASPARTIC PROTEASE IN GUARD CELL 1 |
| TRINITY_DN16469_c3_g1_i4.p1 | 1.3 | 5.4E-04 | 3.1 | 1.0E-12 | protein ASPARTIC PROTEASE IN GUARD CELL 1 |
| TRINITY_DN16469_c3_g1_i1.p1 | 1.5 | 3.3E-05 | 3.0 | 6.8E-10 | protein ASPARTIC PROTEASE IN GUARD CELL 1 |
| TRINITY_DN11976_c0_g1_i1.p1 | 2.4 | 1.7E-05 | 1.6 | 8.4E-05 | protein BIG GRAIN 1-like A |
| TRINITY_DN21292_c0_g1_i6.p1 | 1.0 | 1.2E-05 | 1.5 | 5.3E-11 | protein DETOXIFICATION 27-like |
| TRINITY_DN21292_c0_g1_i26.p1 | 1.3 | 1.5E-08 | 1.1 | 3.3E-06 | protein DETOXIFICATION 27-like |
| TRINITY_DN21322_c1_g2_i5.p1 | 1.3 | 1.7E-09 | 1.1 | 1.7E-05 | protein DETOXIFICATION 27-like |
| TRINITY_DN11131_c0_g1_i1.p1 | 6.1 | 7.6E-05 | 3.4 | 6.6E-03 | protein DETOXIFICATION 51-like |
| TRINITY_DN20909_c0_g1_i1.p2 | 6.0 | 6.3E-30 | 3.1 | 4.0E-11 | protein DMR6-LIKE OXYGENASE 2-like |
| TRINITY_DN20909_c0_g1_i6.p1 | 4.1 | 3.6E-27 | 3.0 | 6.8E-10 | protein DMR6-LIKE OXYGENASE 2-like |
| TRINITY_DN35564_c0_g1_i1.p1 | 6.3 | 1.1E-04 | 3.5 | 9.0E-03 | protein DMR6-LIKE OXYGENASE 2-like |
| TRINITY_DN18686_c4_g6_i1.p1 | 1.5 | 6.3E-07 | 1.9 | 5.6E-09 | protein DOWNY MILDEW RESISTANCE 6 |
| TRINITY_DN10907_c0_g1_i1.p1 | 2.6 | 4.8E-16 | 1.0 | 1.4E-03 | protein E6-like |
| TRINITY_DN19938_c1_g3_i1.p1 | 1.5 | 1.3E-11 | 1.1 | 2.9E-05 | protein FATTY ACID EXPORT 5-like |
| TRINITY_DN10192_c0_g1_i1.p1 | 3.5 | 5.4E-04 | 3.1 | 9.5E-04 | protein GLUTAMINE DUMPER 2-like |
| TRINITY_DN5630_c0_g1_i1.p1 | 3.2 | 1.8E-03 | 3.6 | 1.8E-03 | protein MANNAN SYNTHESIS-RELATED 1-like |
| TRINITY_DN19650_c1_g4_i5.p1 | 2.7 | 3.6E-16 | 1.1 | 4.7E-04 | protein NLP7-like |
| TRINITY_DN18892_c0_g3_i4.p1 | 5.8 | 3.3E-34 | 1.1 | 2.0E-03 | protein NLP7-like |
| TRINITY_DN16355_c0_g6_i1.p1 | 8.5 | 3.6E-17 | 3.4 | 3.1E-06 | protein NRT1/ PTR FAMILY 2.11-like |
| TRINITY_DN21097_c1_g4_i2.p1 | 11.3 | 1.6E-30 | 3.5 | 3.9E-06 | protein NRT1/ PTR FAMILY 2.11-like |
| TRINITY_DN21097_c1_g6_i1.p1 | 6.2 | 3.7E-07 | 3.4 | 4.2E-03 | protein NRT1/ PTR FAMILY 2.11-like |
| TRINITY_DN11588_c0_g1_i1.p1 | 8.4 | 1.5E-14 | 1.8 | 6.4E-03 | protein NRT1/ PTR FAMILY 2.7-like |
| TRINITY_DN8712_c0_g1_i1.p1 | 3.5 | 7.8E-19 | 2.3 | 2.7E-13 | protein NRT1/ PTR FAMILY 4.5-like |
| TRINITY_DN18164_c3_g3_i2.p2 | 4.4 | 1.1E-06 | 2.3 | 6.3E-03 | protein NRT1/ PTR FAMILY 5.10-like |
| TRINITY_DN15327_c0_g5_i1.p1 | 5.2 | 1.9E-13 | 3.3 | 2.1E-04 | protein P21-like |
| TRINITY_DN21520_c0_g1_i17.p1 | 2.2 | 7.8E-09 | 2.2 | 6.3E-08 | protein PHYLLO, chloroplastic isoform X1 |
| TRINITY_DN21056_c0_g5_i3.p1 | 3.6 | 9.1E-14 | 2.6 | 3.7E-07 | protein PHYLLO, chloroplastic isoform X1 |
| TRINITY_DN21364_c1_g1_i10.p1 | 1.8 | 1.4E-04 | 1.9 | 6.2E-04 | protein PHYLLO, chloroplastic isoform X1 |
| TRINITY_DN13462_c0_g1_i2.p1 | 6.1 | 3.1E-14 | 2.5 | 9.6E-04 | protein PIN-LIKES 7-like |
| TRINITY_DN20859_c3_g4_i1.p1 | 10.4 | 4.4E-51 | 3.0 | 1.4E-20 | protein PLANT CADMIUM RESISTANCE 2-like |
| TRINITY_DN20859_c3_g1_i3.p1 | 1.7 | 1.5E-03 | 1.8 | 1.8E-05 | protein PLANT CADMIUM RESISTANCE 2-like |
| TRINITY_DN17402_c2_g4_i3.p1 | 7.9 | 1.9E-09 | 2.9 | 4.3E-03 | protein PLANT CADMIUM RESISTANCE 2-like |
| TRINITY_DN10756_c0_g1_i1.p1 | 4.3 | 1.6E-05 | 2.4 | 9.1E-03 | protein PLANT CADMIUM RESISTANCE 2-like |
| TRINITY_DN14127_c0_g1_i13.p1 | 4.1 | 9.9E-14 | 2.7 | 4.5E-07 | protein SAR DEFICIENT 1-like |
| TRINITY_DN13182_c0_g1_i1.p1 | 4.1 | 1.3E-07 | 3.4 | 5.2E-06 | protein SPEAR3-like |
| TRINITY_DN15560_c2_g4_i1.p1 | 10.7 | 2.6E-55 | 3.7 | 3.2E-07 | protein SRG1-like |
| TRINITY_DN10447_c0_g1_i2.p1 | 8.6 | 3.2E-65 | 2.3 | 1.1E-12 | protein STRICTOSIDINE SYNTHASE-LIKE 10-like |
| TRINITY_DN16890_c1_g3_i1.p1 | 2.3 | 9.9E-06 | 2.5 | 1.5E-12 | protein TIFY 10A-like |
| TRINITY_DN21427_c1_g1_i5.p1 | 4.5 | 9.7E-11 | 1.5 | 7.3E-07 | protein TIFY 10A-like |
| TRINITY_DN13037_c0_g2_i1.p1 | 1.4 | 1.0E-05 | 1.1 | 9.6E-03 | protein tolB |
| TRINITY_DN13634_c0_g1_i3.p1 | 2.1 | 1.6E-09 | 1.6 | 3.5E-06 | protein-tyrosine-phosphatase MKP1-like |
| TRINITY_DN13241_c0_g1_i3.p1 | 2.3 | 1.3E-03 | 1.7 | 2.9E-04 | pseudouridine-5'-phosphate glycosidase |
| TRINITY_DN20562_c0_g1_i1.p1 | 2.1 | 7.1E-17 | 2.3 | 4.6E-17 | putative 12-oxophytodienoate reductase 11 |
| TRINITY_DN20562_c0_g1_i3.p1 | 2.7 | 2.0E-25 | 2.3 | 6.0E-17 | putative 12-oxophytodienoate reductase 11 |
| TRINITY_DN20562_c0_g1_i5.p1 | 3.3 | 2.3E-15 | 2.8 | 6.3E-13 | putative 12-oxophytodienoate reductase 11 |
| TRINITY_DN21714_c1_g1_i15.p1 | 5.3 | 5.1E-19 | 3.5 | 1.7E-12 | putative 12-oxophytodienoate reductase 11 |
| TRINITY_DN21714_c1_g1_i2.p1 | 2.7 | 3.3E-07 | 1.7 | 1.1E-03 | putative 12-oxophytodienoate reductase 11 |
| TRINITY_DN19949_c0_g2_i3.p1 | 3.8 | 1.6E-14 | 1.3 | 5.2E-03 | putative aminoacrylate hydrolase RutD |
| TRINITY_DN20118_c0_g5_i1.p1 | 6.2 | 1.5E-58 | 2.4 | 5.8E-18 | putative calcium-transporting ATPase 13, plasma membrane-type |
| TRINITY_DN17156_c0_g1_i1.p1 | 2.6 | 8.6E-04 | 2.1 | 5.7E-03 | putative disease resistance protein RGA3 |
| TRINITY_DN11353_c0_g1_i1.p1 | 7.0 | 1.6E-07 | 4.3 | 2.4E-05 | putative F-box protein At1g32420 |
| TRINITY_DN16013_c2_g10_i1.p1 | 9.7 | 2.7E-22 | 6.5 | 7.1E-18 | putative germin-like protein 2-1 |
| TRINITY_DN7754_c0_g1_i1.p1 | 2.3 | 1.0E-13 | 1.1 | 5.1E-04 | putative inosine-uridine preferring nucleoside hydrolase |
| TRINITY_DN21166_c0_g7_i1.p1 | 1.8 | 8.0E-09 | 1.5 | 9.0E-08 | putative late blight resistance protein homolog R1A-10 |
| TRINITY_DN16629_c4_g5_i1.p1 | 2.3 | 3.6E-07 | 1.4 | 4.3E-03 | putative late blight resistance protein homolog R1A-10 |
| TRINITY_DN15329_c0_g1_i3.p1 | 1.8 | 7.4E-11 | 1.7 | 1.2E-08 | putative lipase ROG1 |
| TRINITY_DN17602_c0_g2_i2.p1 | 1.6 | 4.1E-05 | 1.3 | 4.7E-03 | putative methylesterase 11, chloroplastic |
| TRINITY_DN17729_c1_g2_i6.p1 | 4.4 | 2.9E-30 | 3.1 | 6.3E-11 | putative methyltransferase DDB_G0268948 |
| TRINITY_DN18321_c0_g2_i1.p1 | 3.4 | 1.6E-38 | 1.5 | 7.6E-06 | putative methyltransferase DDB_G0268948 |
| TRINITY_DN19252_c2_g2_i14.p1 | 4.3 | 2.1E-03 | 5.8 | 1.2E-03 | putative neprosin |
| TRINITY_DN18678_c0_g1_i10.p1 | 1.5 | 3.2E-05 | 1.5 | 2.3E-04 | putative Pentatricopeptide repeat superfamily protein |
| TRINITY_DN10019_c0_g1_i1.p1 | 5.7 | 3.9E-03 | 5.6 | 8.6E-03 | putative plant self-incompatibility S1 |
| TRINITY_DN21705_c0_g2_i1.p1 | 5.7 | 6.8E-16 | 2.9 | 3.2E-08 | putative receptor-like protein kinase At4g00960 |
| TRINITY_DN5925_c0_g1_i1.p1 | 2.7 | 3.4E-03 | 2.5 | 8.1E-03 | putative ribonuclease h protein |
| TRINITY_DN20473_c0_g3_i1.p1 | 3.6 | 3.6E-29 | 1.9 | 3.2E-09 | putative RING-H2 finger protein ATL21A |
| TRINITY_DN10402_c0_g1_i1.p1 | 4.6 | 1.3E-23 | 2.3 | 1.9E-08 | putative RING-H2 finger protein ATL21A |
| TRINITY_DN21133_c0_g2_i1.p1 | 2.6 | 1.8E-07 | 1.5 | 6.9E-04 | putative serine/threonine-protein phosphatase 7-like |
| TRINITY_DN19776_c0_g1_i2.p1 | 9.1 | 1.0E-62 | 3.4 | 1.4E-09 | putative stress up-regulated Nod 19 |
| TRINITY_DN19776_c0_g1_i7.p1 | 13.6 | 9.5E-38 | 3.8 | 4.4E-03 | putative stress up-regulated Nod 19 |
| TRINITY_DN18193_c1_g7_i3.p1 | 1.9 | 4.6E-07 | 1.4 | 1.6E-04 | putative Tetratricopeptide repeat (TPR)-like superfamily protein |
| TRINITY_DN2154_c0_g2_i1.p1 | 6.8 | 1.1E-05 | 6.8 | 4.3E-05 | putative transcription factor bHLH041 |
| TRINITY_DN17539_c1_g1_i1.p1 | 3.1 | 5.0E-05 | 1.7 | 5.9E-04 | putative UDP-rhamnose:rhamnosyltransferase 1 |
| TRINITY_DN11258_c0_g1_i1.p1 | 2.5 | 4.3E-16 | 1.0 | 5.2E-03 | putative UDP-rhamnose:rhamnosyltransferase 1 |
| TRINITY_DN18235_c0_g3_i1.p1 | 4.8 | 4.1E-74 | 1.3 | 7.0E-07 | putative UPF0481 protein At3g02645 |
| TRINITY_DN21643_c2_g1_i1.p1 | 1.1 | 4.1E-03 | 1.3 | 1.8E-03 | pyrophosphate-energized vacuolar membrane proton pump |
| TRINITY_DN20495_c2_g1_i2.p1 | 1.3 | 3.3E-03 | 2.0 | 3.2E-05 | random slug protein 5 |
| TRINITY_DN16306_c1_g1_i2.p1 | 1.5 | 3.1E-03 | 2.2 | 2.1E-05 | receptor-like protein 15 |
| TRINITY_DN18498_c5_g1_i1.p1 | 2.7 | 5.0E-04 | 2.2 | 5.2E-03 | receptor-like protein 15 |
| TRINITY_DN16507_c0_g1_i11.p1 | 4.4 | 4.5E-03 | 5.5 | 9.4E-03 | receptor-like protein 15 |
| TRINITY_DN13503_c0_g1_i1.p1 | 1.8 | 7.9E-12 | 1.4 | 1.2E-06 | receptor-like protein 46 |
| TRINITY_DN15987_c2_g9_i1.p1 | 5.1 | 3.6E-25 | 2.2 | 1.2E-06 | receptor-like protein kinase HAIKU2 |
| TRINITY_DN18672_c2_g2_i1.p1 | 3.2 | 1.9E-14 | 2.2 | 5.2E-11 | receptor-like protein kinase HSL1 |
| TRINITY_DN15987_c1_g1_i3.p1 | 2.9 | 5.0E-11 | 1.6 | 7.9E-05 | receptor-like serine/threonine-protein kinase At1g78530 |
| TRINITY_DN20844_c0_g4_i5.p1 | 2.2 | 1.5E-06 | 1.7 | 1.2E-03 | receptor-like serine/threonine-protein kinase SD1-8 |
| TRINITY_DN17441_c1_g5_i1.p1 | 2.2 | 3.2E-11 | 1.4 | 8.4E-05 | respiratory burst oxidase homolog protein C-like |
| TRINITY_DN22221_c1_g1_i2.p1 | 1.5 | 7.4E-04 | 1.3 | 6.9E-03 | retrovirus-related Pol polyprotein from transposon TNT 1-94 |
| TRINITY_DN13815_c0_g1_i1.p1 | 1.2 | 6.8E-03 | 1.7 | 2.5E-04 | rust resistance kinase Lr10-like |
| TRINITY_DN18932_c0_g1_i4.p1 | 3.9 | 1.7E-18 | 2.6 | 5.7E-06 | salicylate carboxymethyltransferase-like |
| TRINITY_DN18932_c0_g1_i3.p1 | 10.1 | 8.4E-34 | 2.4 | 7.8E-04 | salicylate carboxymethyltransferase-like |
| TRINITY_DN11727_c0_g1_i1.p1 | 3.9 | 3.3E-21 | 3.3 | 1.5E-16 | scarecrow-like protein 14 |
| TRINITY_DN20140_c1_g3_i15.p2 | 2.6 | 2.7E-08 | 1.9 | 2.0E-04 | scopoletin glucosyltransferase-like |
| TRINITY_DN19976_c1_g2_i1.p1 | 6.4 | 9.6E-37 | 1.8 | 4.8E-04 | scopoletin glucosyltransferase-like |
| TRINITY_DN19473_c1_g2_i2.p1 | 3.7 | 1.6E-14 | 3.8 | 7.5E-09 | secoisolariciresinol dehydrogenase-like |
| TRINITY_DN19473_c1_g2_i6.p1 | 7.9 | 2.6E-64 | 2.0 | 3.5E-06 | secoisolariciresinol dehydrogenase-like |
| TRINITY_DN18095_c2_g1_i5.p1 | 1.4 | 1.9E-04 | 1.8 | 3.5E-05 | secoisolariciresinol dehydrogenase-like |
| TRINITY_DN12403_c0_g1_i1.p1 | 4.3 | 7.4E-09 | 2.9 | 5.3E-05 | secoisolariciresinol dehydrogenase-like |
| TRINITY_DN19473_c1_g2_i7.p1 | 10.0 | 8.7E-55 | 3.0 | 1.4E-04 | secoisolariciresinol dehydrogenase-like |
| TRINITY_DN31470_c0_g1_i1.p1 | 3.9 | 1.2E-08 | 5.2 | 4.9E-11 | senescence-specific cysteine protease SAG39-like |
| TRINITY_DN15071_c0_g2_i4.p1 | 1.5 | 1.4E-04 | 1.7 | 9.6E-06 | serine carboxypeptidase-like |
| TRINITY_DN19734_c0_g1_i1.p1 | 2.9 | 3.8E-15 | 2.1 | 2.2E-09 | serine carboxypeptidase-like 26 |
| TRINITY_DN15934_c0_g2_i2.p1 | 2.3 | 5.8E-12 | 1.3 | 1.5E-04 | serine carboxypeptidase-like 34 |
| TRINITY_DN20687_c0_g5_i3.p1 | 1.2 | 5.6E-08 | 1.0 | 2.2E-04 | shikimate O-hydroxycinnamoyltransferase-like |
| TRINITY_DN20165_c1_g3_i1.p1 | 4.1 | 1.8E-17 | 1.1 | 7.9E-03 | short-chain dehydrogenase reductase 2a |
| TRINITY_DN14469_c0_g1_i6.p1 | 4.7 | 2.6E-17 | 3.1 | 2.3E-10 | short-chain dehydrogenase TIC 32, chloroplastic |
| TRINITY_DN14469_c0_g1_i2.p1 | 2.1 | 2.2E-03 | 2.6 | 5.7E-03 | short-chain dehydrogenase TIC 32, chloroplastic |
| TRINITY_DN11322_c0_g1_i1.p1 | 7.9 | 6.0E-45 | 2.3 | 1.9E-06 | slit homolog 2 protein-like isoform X2 |
| TRINITY_DN20593_c0_g1_i1.p1 | 6.3 | 2.3E-48 | 1.8 | 2.6E-05 | S-norcoclaurine synthase 1-like |
| TRINITY_DN11787_c0_g1_i1.p1 | 2.6 | 2.3E-13 | 1.7 | 4.0E-07 | S-norcoclaurine synthase 2-like |
| TRINITY_DN15849_c0_g2_i3.p1 | 3.4 | 2.9E-39 | 3.1 | 5.5E-12 | somatic embryogenesis receptor kinase 2 |
| TRINITY_DN11234_c0_g1_i1.p1 | 2.0 | 3.8E-04 | 2.6 | 1.4E-04 | sphinganine C4-monooxygenase 1-like |
| TRINITY_DN16558_c3_g1_i2.p1 | 6.2 | 1.8E-28 | 3.6 | 4.1E-11 | squalene monooxygenase-like |
| TRINITY_DN16558_c4_g1_i6.p1 | 6.7 | 1.0E-39 | 2.2 | 2.6E-07 | squalene monooxygenase-like |
| TRINITY_DN19171_c0_g1_i3.p1 | 3.1 | 3.2E-21 | 1.5 | 4.8E-03 | stearoyl-[acyl-carrier-protein] 9-desaturase, chloroplastic |
| TRINITY_DN11548_c1_g1_i1.p1 | 5.4 | 4.8E-09 | 2.2 | 2.1E-03 | stigma-specific STIG1-like protein 1 |
| TRINITY_DN19776_c0_g1_i6.p1 | 10.0 | 4.7E-34 | 3.6 | 4.2E-03 | stress up-regulated Nod 19 |
| TRINITY_DN19579_c0_g4_i1.p1 | 1.7 | 2.4E-09 | 1.9 | 2.8E-14 | subtilisin-like protease SBT1.7 |
| TRINITY_DN14282_c0_g2_i2.p1 | 2.0 | 4.0E-04 | 3.8 | 6.4E-09 | subtilisin-like protease SBT1.7 |
| TRINITY_DN21938_c2_g6_i1.p1 | 7.2 | 4.3E-18 | 4.5 | 3.0E-10 | subtilisin-like protease SBT1.9 |
| TRINITY_DN21938_c1_g3_i1.p1 | 10.1 | 1.6E-25 | 4.4 | 7.5E-10 | subtilisin-like protease SBT1.9 |
| TRINITY_DN13822_c0_g1_i2.p1 | 3.5 | 4.1E-12 | 2.7 | 8.1E-06 | sugar transport protein 13-like |
| TRINITY_DN16100_c1_g4_i1.p1 | 7.7 | 5.9E-40 | 1.6 | 2.4E-05 | sulfate transporter 1.3-like |
| TRINITY_DN18149_c0_g1_i2.p1 | 2.1 | 1.7E-04 | 1.7 | 8.6E-04 | superoxide dismutase [Cu-Zn] |
| TRINITY_DN12127_c0_g1_i1.p1 | 4.0 | 1.1E-06 | 3.0 | 3.7E-04 | terminal ear1 -like protein |
| TRINITY_DN4613_c0_g1_i1.p1 | 5.1 | 5.8E-09 | 4.1 | 4.3E-07 | terpene synthase 10-like |
| TRINITY_DN15327_c0_g1_i6.p1 | 5.1 | 2.1E-06 | 3.0 | 7.0E-03 | thaumatin-like protein |
| TRINITY_DN13897_c0_g2_i2.p1 | 1.8 | 9.2E-14 | 1.0 | 2.3E-03 | thioredoxin-like protein |
| TRINITY_DN17267_c1_g6_i1.p1 | 6.6 | 1.4E-31 | 2.2 | 3.7E-07 | trans-cinnamate 4-monooxygenase |
| TRINITY_DN10226_c0_g1_i2.p1 | 4.7 | 4.1E-09 | 2.3 | 4.5E-04 | transcription factor bHLH162-like |
| TRINITY_DN18988_c1_g1_i4.p1 | 1.3 | 2.6E-03 | 2.1 | 1.3E-08 | transcription factor bHLH18-like |
| TRINITY_DN18988_c1_g2_i4.p1 | 1.3 | 1.6E-04 | 1.3 | 2.4E-03 | transcription factor bHLH18-like |
| TRINITY_DN20719_c0_g1_i11.p1 | 3.6 | 1.1E-21 | 3.2 | 2.9E-24 | transcription factor bHLH30-like |
| TRINITY_DN19021_c0_g1_i3.p1 | 2.5 | 2.9E-09 | 3.3 | 2.0E-18 | transcription factor bHLH30-like |
| TRINITY_DN10746_c0_g1_i1.p1 | 4.1 | 8.3E-06 | 6.7 | 4.4E-07 | transcription factor JUNGBRUNNEN 1-like |
| TRINITY_DN11686_c0_g1_i2.p1 | 6.1 | 2.2E-15 | 3.2 | 1.5E-03 | transcription factor JUNGBRUNNEN 1-like |
| TRINITY_DN14348_c0_g1_i1.p1 | 2.2 | 4.0E-07 | 1.3 | 2.5E-04 | transcription factor MYB44-like |
| TRINITY_DN18579_c1_g1_i1.p1 | 5.4 | 9.0E-08 | 2.9 | 2.0E-03 | transcription factor RAX2-like |
| TRINITY_DN16782_c0_g1_i16.p1 | 1.4 | 1.4E-05 | 1.1 | 3.2E-03 | transcription factor TGA2.3-like isoform X1 |
| TRINITY_DN10423_c0_g1_i1.p1 | 7.3 | 4.9E-11 | 7.2 | 2.0E-10 | transmembrane protein |
| TRINITY_DN12580_c0_g1_i1.p1 | 4.6 | 1.0E-08 | 3.8 | 1.8E-06 | transmembrane protein |
| TRINITY_DN1026_c0_g1_i1.p1 | 3.1 | 2.3E-03 | 5.6 | 1.8E-05 | transmembrane protein |
| TRINITY_DN13424_c1_g1_i1.p1 | 7.6 | 1.3E-07 | 4.1 | 2.4E-04 | transmembrane protein |
| TRINITY_DN17868_c0_g2_i5.p1 | 1.8 | 2.9E-08 | 1.2 | 1.3E-03 | transmembrane protein 256 homolog |
| TRINITY_DN13446_c0_g1_i1.p1 | 2.7 | 1.4E-05 | 2.7 | 3.5E-04 | transmembrane protein 45B-like |
| TRINITY_DN14077_c0_g2_i2.p1 | 1.7 | 1.3E-06 | 1.6 | 1.5E-04 | triacylglycerol lipase 2-like |
| TRINITY_DN13667_c0_g1_i2.p1 | 2.5 | 5.5E-11 | 1.2 | 3.5E-03 | ubiquitin-like domain-containing CTD phosphatase 1 isoform X1 |
| TRINITY_DN11756_c0_g1_i1.p1 | 1.8 | 2.4E-10 | 1.4 | 3.2E-05 | U-box domain-containing protein 35 |
| TRINITY_DN11106_c0_g1_i1.p1 | 3.7 | 2.3E-16 | 1.9 | 1.2E-10 | uclacyanin 1-like |
| TRINITY_DN19082_c0_g2_i6.p1 | 3.3 | 7.1E-15 | 1.1 | 5.8E-03 | UDP-galactose/UDP-glucose transporter 2-like |
| TRINITY_DN12635_c0_g1_i7.p1 | 1.7 | 5.2E-07 | 2.4 | 3.6E-11 | UDP-glucose 6-dehydrogenase 1 |
| TRINITY_DN7675_c0_g1_i1.p1 | 2.1 | 7.2E-03 | 3.2 | 2.7E-04 | UDP-glucose iridoid glucosyltransferase-like |
| TRINITY_DN16353_c3_g1_i1.p1 | 1.5 | 1.5E-05 | 1.3 | 5.9E-04 | UDP-glucuronic acid decarboxylase 2-like |
| TRINITY_DN11487_c0_g1_i1.p1 | 7.1 | 3.4E-20 | 4.5 | 8.0E-11 | UDP-glycosyltransferase 73D1-like |
| TRINITY_DN14573_c0_g1_i1.p1 | 5.7 | 1.0E-11 | 3.8 | 5.2E-07 | UDP-glycosyltransferase 87A1-like |
| TRINITY_DN9890_c0_g1_i2.p1 | 1.3 | 5.0E-05 | 1.6 | 5.7E-07 | UDP-glycosyltransferase 89B2-like |
| TRINITY_DN15199_c1_g3_i2.p1 | 3.1 | 8.7E-24 | 2.7 | 1.8E-16 | uncharacterized acetyltransferase At3g50280-like |
| TRINITY_DN15199_c1_g3_i1.p1 | 7.5 | 2.9E-19 | 2.6 | 1.1E-04 | uncharacterized acetyltransferase At3g50280-like |
| TRINITY_DN17580_c1_g2_i9.p1 | 2.3 | 4.9E-13 | 2.1 | 3.2E-06 | uncharacterized isomerase BH0283-like |
| TRINITY_DN19454_c0_g1_i20.p1 | 3.0 | 1.4E-29 | 1.2 | 1.2E-04 | uncharacterized isomerase BH0283-like |
| TRINITY_DN11342_c0_g1_i1.p1 | 6.2 | 6.1E-16 | 2.7 | 4.3E-05 | uncharacterized protein LOC113703525 isoform X2 |
| TRINITY_DN12545_c0_g1_i1.p1 | 4.1 | 1.1E-04 | 4.8 | 5.5E-05 | universal stress protein PHOS32 |
| TRINITY_DN18233_c0_g1_i2.p2 | 4.3 | 1.5E-43 | 2.2 | 8.4E-19 | UPF0339 protein BP0521 |
| TRINITY_DN15418_c0_g1_i3.p1 | 1.2 | 7.2E-03 | 1.2 | 3.4E-03 | UPF0481 protein At3g47200-like |
| TRINITY_DN14831_c0_g2_i1.p1 | 1.4 | 4.2E-08 | 1.1 | 1.5E-04 | vacuolar-sorting receptor 6-like |
| TRINITY_DN18603_c2_g1_i3.p1 | 2.3 | 8.0E-16 | 2.3 | 5.9E-19 | valine--tRNA ligase |
| TRINITY_DN18603_c2_g5_i1.p1 | 7.8 | 4.0E-12 | 2.9 | 2.3E-05 | valine--tRNA ligase |
| TRINITY_DN15181_c3_g1_i1.p1 | 3.5 | 6.9E-16 | 1.6 | 1.7E-04 | vegetative cell wall protein gp1-like isoform X1 |
| TRINITY_DN13949_c1_g2_i1.p1 | 8.3 | 1.1E-11 | 3.9 | 4.9E-06 | vestitone reductase-like |
| TRINITY_DN11540_c0_g1_i1.p1 | 7.4 | 9.8E-12 | 3.2 | 1.1E-06 | vicilin-like seed storage protein At2g28490 |
| TRINITY_DN376_c0_g1_i1.p1 | 4.5 | 8.6E-13 | 2.4 | 8.3E-07 | vinorine synthase-like |
| TRINITY_DN11262_c0_g1_i1.p1 | 1.7 | 1.5E-04 | 1.7 | 5.3E-07 | VPS10 2 like |
| TRINITY_DN9644_c0_g1_i1.p1 | 2.5 | 1.0E-03 | 2.5 | 1.7E-03 | VQ motif containing protein |
| TRINITY_DN21522_c1_g1_i3.p1 | 2.1 | 1.8E-06 | 2.0 | 2.3E-05 | VQ motif-containing protein |
| TRINITY_DN21522_c1_g1_i1.p1 | 3.5 | 5.3E-06 | 1.9 | 6.4E-03 | VQ motif-containing protein |
| TRINITY_DN10370_c0_g1_i1.p1 | 5.4 | 3.4E-04 | 4.1 | 7.3E-03 | VQ motif-containing protein 1-like |
| TRINITY_DN11954_c0_g1_i1.p1 | 5.1 | 1.8E-06 | 3.4 | 5.1E-04 | VQ motif-containing protein 22 |
| TRINITY_DN17831_c3_g3_i1.p1 | 7.7 | 1.3E-26 | 3.7 | 6.7E-12 | VQ motif-containing protein 22-like |
| TRINITY_DN17424_c0_g3_i3.p1 | 2.7 | 1.8E-08 | 3.6 | 3.4E-11 | wall-associated receptor kinase 2-like |
| TRINITY_DN12773_c0_g1_i1.p1 | 4.7 | 3.7E-09 | 2.7 | 1.4E-04 | WEB family protein At1g12150-like |
| TRINITY_DN12079_c0_g1_i1.p1 | 6.9 | 1.8E-35 | 1.8 | 6.2E-06 | xyloglucan endotransglucosylase/hydrolase 2-like |
| TRINITY_DN12708_c0_g1_i1.p1 | 1.7 | 1.4E-03 | 2.3 | 2.4E-05 | ZCW7 isoform 1 |
| TRINITY_DN17507_c0_g1_i7.p1 | 9.7 | 9.8E-22 | 3.0 | 8.9E-06 | zeatin O-glucosyltransferase-like |
| TRINITY_DN17507_c0_g1_i3.p1 | 4.9 | 3.0E-16 | 2.0 | 4.5E-05 | zeatin O-glucosyltransferase-like |
| TRINITY_DN15452_c0_g1_i1.p1 | 3.6 | 5.9E-10 | 2.2 | 3.0E-04 | zeatin O-glucosyltransferase-like |
| TRINITY_DN11455_c0_g1_i1.p1 | 3.9 | 5.4E-26 | 2.1 | 3.5E-06 | zinc finger protein 5-like |
| TRINITY_DN14537_c0_g1_i2.p1 | 5.0 | 1.1E-18 | 2.6 | 4.0E-09 | zinc finger protein WIP3-like |
| TRINITY_DN10303_c0_g1_i3.p1 | 6.6 | 4.6E-22 | 1.8 | 3.3E-06 | zinc finger, PHD-type |

*FC: fold change, **FDR: false discovery rate, ***NA: not available

| **Table S5** Genes downregulated in basal region of internodal segments. | | | | | |
| --- | --- | --- | --- | --- | --- |
|  | 1b/0b | | 1b/1a | |  |
|  | logFC* | FDR** | logFC* | FDR** | description |
| TRINITY_DN14209_c0_g1_i4.p1 | -2.3 | 4.2E-16 | -1.7 | 3.2E-09 | 11-*β*-hydroxysteroid dehydrogenase 1B-like |
| TRINITY_DN12814_c0_g1_i3.p1 | -1.7 | 2.6E-07 | -1.7 | 1.6E-07 | 1-aminocyclopropane-1-carboxylate oxidase homolog 1-like |
| TRINITY_DN15561_c0_g4_i2.p1 | -1.1 | 1.3E-03 | -1.4 | 1.6E-07 | 3-ketoacyl-CoA synthase 11-like |
| TRINITY_DN13041_c2_g1_i4.p1 | -3.6 | 1.9E-07 | -3.7 | 1.5E-13 | ABC transporter G family member 15-like |
| TRINITY_DN14264_c0_g1_i1.p1 | -1.8 | 7.8E-06 | -2.9 | 1.0E-21 | adenine nucleotide *α* hydrolases-like superfamily protein |
| TRINITY_DN17768_c0_g1_i11.p1 | -1.4 | 7.9E-04 | -2.1 | 3.2E-09 | *α*,*α*-trehalose-phosphate synthase [UDP-forming] 1-like |
| TRINITY_DN19770_c1_g3_i12.p1 | -1.5 | 2.8E-06 | -1.2 | 7.8E-04 | anthranilate phosphoribosyltransferase, chloroplastic |
| TRINITY_DN20124_c0_g4_i1.p1 | -1.7 | 4.4E-07 | -1.5 | 1.8E-05 | anthranilate synthase *α* subunit 1, chloroplastic-like |
| TRINITY_DN20670_c0_g1_i1.p1 | -2.5 | 7.8E-25 | -1.3 | 1.1E-05 | BAG family molecular chaperone regulator 1-like |
| TRINITY_DN21421_c1_g1_i1.p1 | -3.4 | 2.4E-32 | -1.8 | 3.5E-10 | BAHD acyltransferase DCR |
| TRINITY_DN18445_c4_g1_i2.p1 | -1.2 | 1.4E-03 | -1.4 | 2.2E-04 | bifunctional epoxide hydrolase 2-like |
| TRINITY_DN17571_c0_g3_i1.p2 | -1.1 | 8.6E-05 | -1.4 | 2.0E-07 | classical arabinogalactan protein 26 |
| TRINITY_DN13974_c0_g1_i1.p1 | -3.1 | 1.6E-28 | -1.8 | 8.1E-10 | cytochrome P450 77A3 |
| TRINITY_DN18925_c0_g4_i2.p1 | -2.1 | 5.0E-14 | -1.3 | 4.4E-05 | cytochrome P450 86A8 |
| TRINITY_DN18925_c0_g4_i1.p1 | -3.4 | 1.2E-25 | -1.5 | 6.1E-03 | cytochrome P450 86A8 |
| TRINITY_DN12847_c0_g1_i2.p1 | -3.2 | 8.9E-06 | -5.6 | 3.3E-39 | equilibrative nucleotide transporter 1 |
| TRINITY_DN19301_c2_g1_i1.p1 | -3.9 | 8.1E-09 | -2.8 | 3.2E-03 | equilibrative nucleotide transporter 3-like |
| TRINITY_DN21705_c0_g1_i6.p1 | -2.6 | 9.8E-12 | -3.0 | 4.7E-17 | extracellular ribonuclease LE-like |
| TRINITY_DN21705_c1_g2_i1.p1 | -2.4 | 1.5E-07 | -2.8 | 6.5E-12 | extracellular ribonuclease LE-like |
| TRINITY_DN21705_c0_g1_i8.p1 | -1.9 | 1.9E-06 | -1.9 | 1.7E-04 | extracellular ribonuclease LE-like |
| TRINITY_DN15565_c0_g1_i8.p1 | -1.7 | 6.9E-06 | -1.4 | 2.6E-04 | F-box protein At2g26850-like |
| TRINITY_DN13717_c0_g1_i1.p1 | -1.4 | 2.1E-03 | -1.9 | 1.5E-08 | ferric reduction oxidase 2-like |
| TRINITY_DN12927_c0_g2_i2.p1 | -4.4 | 1.2E-06 | -5.0 | 3.2E-11 | gata zinc finger protein |
| TRINITY_DN12927_c0_g1_i2.p1 | -4.1 | 2.0E-03 | -5.1 | 8.9E-07 | GATA zinc finger protein |
| TRINITY_DN9783_c0_g1_i1.p1 | -2.7 | 1.5E-04 | -3.7 | 2.1E-18 | GDSL esterase/lipase At1g71691-like |
| TRINITY_DN12779_c0_g1_i4.p1 | -3.7 | 4.0E-17 | -1.9 | 3.8E-04 | GDSL esterase/lipase At5g33370-like |
| TRINITY_DN19015_c2_g1_i1.p1 | -2.0 | 7.5E-05 | -2.1 | 3.6E-04 | glutamic acid-rich protein-like |
| TRINITY_DN17716_c0_g1_i3.p1 | -3.9 | 1.3E-05 | -3.3 | 8.3E-04 | growth-regulating factor 1 isoform X2 |
| TRINITY_DN16914_c0_g1_i3.p1 | -1.6 | 4.3E-04 | -2.5 | 1.9E-19 | growth-regulating factor 1-like |
| TRINITY_DN17078_c1_g1_i3.p1 | -1.7 | 2.8E-04 | -2.9 | 1.7E-15 | growth-regulating factor 1-like |
| TRINITY_DN13593_c0_g1_i1.p1 | -1.8 | 1.3E-05 | -1.5 | 2.0E-03 | G-type lectin S-receptor-like serine/threonine-protein kinase SD3-1 |
| TRINITY_DN14817_c0_g1_i1.p1 | -1.8 | 1.8E-03 | -2.4 | 1.9E-06 | heavy metal-associated isoprenylated plant protein 35-like |
| TRINITY_DN14110_c0_g1_i1.p1 | -2.1 | 1.6E-13 | -1.1 | 3.0E-03 | homeobox-leucine zipper protein ROC3 |
| TRINITY_DN16623_c0_g1_i1.p1 | -1.1 | 8.9E-05 | -1.1 | 9.8E-05 | interactor of constitutive active ROPs 4-like |
| TRINITY_DN19630_c2_g1_i2.p1 | -1.7 | 4.8E-09 | -1.4 | 8.5E-05 | IRK-interacting protein |
| TRINITY_DN16361_c0_g1_i3.p1 | -2.0 | 3.3E-05 | -2.6 | 3.2E-09 | kirola-like |
| TRINITY_DN21241_c0_g3_i1.p1 | -4.9 | 1.2E-21 | -3.3 | 6.6E-09 | kirola-like |
| TRINITY_DN12367_c0_g1_i1.p1 | -1.2 | 3.3E-05 | -1.5 | 3.8E-09 | leucine-rich repeat receptor protein kinase EMS1 |
| TRINITY_DN12879_c0_g1_i2.p1 | -2.4 | 2.0E-15 | -2.6 | 5.6E-19 | leucine-rich repeat receptor-like serine/threonine-protein kinase BAM1 |
| TRINITY_DN14921_c0_g1_i4.p1 | -1.5 | 2.4E-07 | -1.5 | 4.8E-07 | long chain acyl-CoA synthetase 2 |
| TRINITY_DN16373_c0_g2_i1.p1 | -2.1 | 3.3E-11 | -2.2 | 3.0E-06 | miraculin-like |
| TRINITY_DN15530_c2_g3_i1.p1 | -2.7 | 2.9E-17 | -1.2 | 1.8E-03 | miraculin-like |
| TRINITY_DN10614_c0_g1_i2.p1 | -2.0 | 4.7E-05 | -3.0 | 6.8E-22 | MLP-like protein 423 |
| TRINITY_DN9740_c0_g1_i1.p2 | -4.2 | 5.5E-04 | -6.5 | 1.6E-31 | ---NA***--- |
| TRINITY_DN11103_c0_g1_i2.p1 | -2.3 | 4.8E-11 | -2.7 | 4.9E-23 | ---NA--- |
| TRINITY_DN16869_c1_g1_i2.p1 | -1.8 | 4.0E-10 | -1.6 | 1.7E-07 | ---NA--- |
| TRINITY_DN9772_c0_g1_i1.p1 | -1.3 | 9.1E-04 | -1.4 | 5.2E-04 | non-specific lipid transfer protein GPI-anchored 1 |
| TRINITY_DN17312_c0_g6_i1.p1 | -2.4 | 4.5E-16 | -1.8 | 6.6E-12 | PLAT domain-containing protein 3-like |
| TRINITY_DN15851_c2_g6_i2.p1 | -6.0 | 3.4E-77 | -3.7 | 2.9E-19 | pollen ole e 1 allergen and extensin family protein |
| TRINITY_DN16752_c6_g2_i2.p1 | -2.6 | 1.4E-10 | -2.1 | 5.8E-05 | probable aquaporin NIP5-1 |
| TRINITY_DN16461_c0_g1_i8.p1 | -1.0 | 9.8E-06 | -1.1 | 2.3E-06 | probable cinnamyl alcohol dehydrogenase 6 |
| TRINITY_DN14327_c1_g1_i3.p1 | -2.0 | 7.3E-04 | -2.2 | 1.6E-04 | probable leucine-rich repeat receptor-like protein kinase At5g63930 |
| TRINITY_DN14296_c0_g1_i1.p1 | -2.6 | 1.3E-09 | -1.8 | 5.3E-04 | probable lysophospholipase BODYGUARD 3 |
| TRINITY_DN14362_c0_g1_i2.p1 | -2.0 | 2.5E-06 | -1.9 | 1.5E-05 | probable xyloglucan endotransglucosylase/hydrolase protein 33 |
| TRINITY_DN19915_c1_g4_i1.p1 | -3.8 | 1.6E-04 | -6.0 | 1.3E-31 | protein ECERIFERUM 1-like |
| TRINITY_DN12982_c0_g1_i1.p1 | -2.6 | 1.0E-06 | -2.5 | 4.1E-05 | protein ECERIFERUM 26-like |
| TRINITY_DN18594_c1_g1_i10.p1 | -1.3 | 5.1E-04 | -1.2 | 5.1E-03 | protein ENHANCED DISEASE RESISTANCE like |
| TRINITY_DN16995_c3_g3_i2.p1 | -3.1 | 7.2E-14 | -2.3 | 2.6E-08 | protein IQ-DOMAIN 1-like |
| TRINITY_DN22028_c0_g1_i4.p1 | -1.4 | 9.5E-06 | -1.5 | 7.2E-06 | protein NRT1/ PTR FAMILY 3.1-like |
| TRINITY_DN14213_c0_g1_i4.p1 | -2.3 | 3.5E-11 | -4.3 | 1.4E-41 | protein RICE SALT SENSITIVE 3-like |
| TRINITY_DN20284_c0_g3_i3.p1 | -2.8 | 1.0E-03 | -5.3 | 8.6E-29 | protein STICHEL-like 2 |
| TRINITY_DN16071_c1_g1_i1.p1 | -6.6 | 3.9E-23 | -4.2 | 4.9E-06 | protein STRICTOSIDINE SYNTHASE-LIKE 10-like |
| TRINITY_DN20357_c0_g1_i4.p1 | -3.1 | 2.2E-09 | -2.7 | 7.4E-05 | protein trichome birefringence-like 10 |
| TRINITY_DN14901_c0_g1_i1.p1 | -2.0 | 2.4E-03 | -2.0 | 3.4E-03 | purine-uracil permease NCS1 |
| TRINITY_DN22155_c1_g1_i1.p1 | -1.4 | 8.9E-03 | -1.1 | 9.4E-03 | putative disease resistance protein RGA3 |
| TRINITY_DN15570_c0_g1_i6.p1 | -1.5 | 9.8E-09 | -1.0 | 2.7E-04 | putative E3 ubiquitin-protein ligase LIN-1 |
| TRINITY_DN19975_c0_g2_i8.p1 | -3.0 | 1.3E-12 | -4.0 | 5.6E-24 | putative LuxR transcriptional regulator |
| TRINITY_DN16725_c0_g2_i10.p1 | -1.8 | 3.3E-05 | -1.9 | 9.8E-05 | putative serine/threonine-protein kinase isoform X1 |
| TRINITY_DN20370_c0_g1_i1.p1 | -2.1 | 2.7E-03 | -2.4 | 4.3E-03 | putative *S*-locus lectin protein kinase family protein |
| TRINITY_DN14425_c0_g1_i2.p1 | -2.9 | 3.8E-04 | -5.3 | 2.3E-27 | receptor-like protein kinase 2 |
| TRINITY_DN19035_c2_g6_i1.p1 | -3.7 | 5.3E-04 | -3.7 | 3.0E-05 | retrovirus-related Pol polyprotein LINE-1 |
| TRINITY_DN11651_c0_g1_i2.p1 | -1.4 | 1.2E-04 | -1.6 | 2.0E-06 | serine/threonine-protein kinase-like protein ACR4 |
| TRINITY_DN15255_c0_g1_i1.p2 | -3.6 | 3.2E-15 | -2.6 | 1.8E-23 | snakin-2-like |
| TRINITY_DN17838_c0_g3_i3.p1 | -4.1 | 4.0E-03 | -4.1 | 4.7E-04 | staphylococcal-like nuclease CAN2 |
| TRINITY_DN10494_c0_g1_i1.p1 | -3.8 | 2.4E-19 | -3.7 | 7.6E-19 | subtilisin-like protease SBT1.3 |
| TRINITY_DN20042_c0_g2_i2.p1 | -2.4 | 3.1E-15 | -1.7 | 1.8E-04 | transcription factor bHLH63-like |
| TRINITY_DN20351_c0_g4_i4.p1 | -6.2 | 6.3E-03 | -8.2 | 9.2E-17 | vinorine synthase-like |
| TRINITY_DN15084_c0_g1_i3.p1 | -1.8 | 2.2E-07 | -1.6 | 1.6E-05 | WD repeat-containing protein 44 |
| TRINITY_DN16418_c0_g4_i1.p1 | -1.9 | 4.5E-09 | -1.6 | 2.1E-06 | zinc-finger homeodomain protein 6 |

*FC: fold change, **FDR: false discovery rate, ***NA: not available
